# Path to Clonal Theranostics in Luminal Breast Cancers

**DOI:** 10.3389/fonc.2021.802177

**Published:** 2022-01-13

**Authors:** Nawale Hajjaji, Soulaimane Aboulouard, Tristan Cardon, Delphine Bertin, Yves-Marie Robin, Isabelle Fournier, Michel Salzet

**Affiliations:** ^1^ Univ. Lille, Inserm, CHU Lille, U1192, Laboratoire Protéomique, Réponse Inflammatoire et Spectrométrie de Masse (PRISM), Lille, France; ^2^ Breast Cancer Unit, Oscar Lambret Center, Lille, France; ^3^ Institut universitaire de France, Paris, France

**Keywords:** functional tumor heterogeneity, spatially resolved MALDI mass spectrometry imaging, microproteomics, spatially resolved proteome, luminal breast cancers, clonal theranostics, mutated and alternative proteomes, drug repurposing and drug target discovery

## Abstract

Integrating tumor heterogeneity in the drug discovery process is a key challenge to tackle breast cancer resistance. Identifying protein targets for functionally distinct tumor clones is particularly important to tailor therapy to the heterogeneous tumor subpopulations and achieve clonal theranostics. For this purpose, we performed an unsupervised, label-free, spatially resolved shotgun proteomics guided by MALDI mass spectrometry imaging (MSI) on 124 selected tumor clonal areas from early luminal breast cancers, tumor stroma, and breast cancer metastases. 2868 proteins were identified. The main protein classes found in the clonal proteome dataset were enzymes, cytoskeletal proteins, membrane-traffic, translational or scaffold proteins, or transporters. As a comparison, gene-specific transcriptional regulators, chromatin related proteins or transmembrane signal receptor were more abundant in the TCGA dataset. Moreover, 26 mutated proteins have been identified. Similarly, expanding the search to alternative proteins databases retrieved 126 alternative proteins in the clonal proteome dataset. Most of these alternative proteins were coded mainly from non-coding RNA. To fully understand the molecular information brought by our approach and its relevance to drug target discovery, the clonal proteomic dataset was further compared to the TCGA breast cancer database and two transcriptomic panels, BC360 (nanoString^®^) and CDx (Foundation One^®^). We retrieved 139 pathways in the clonal proteome dataset. Only 55% of these pathways were also present in the TCGA dataset, 68% in BC360 and 50% in CDx. Seven of these pathways have been suggested as candidate for drug targeting, 22 have been associated with breast cancer in experimental or clinical reports, the remaining 19 pathways have been understudied in breast cancer. Among the anticancer drugs, 35 drugs matched uniquely with the clonal proteome dataset, with only 7 of them already approved in breast cancer. The number of target and drug interactions with non-anticancer drugs (such as agents targeting the cardiovascular system, metabolism, the musculoskeletal or the nervous systems) was higher in the clonal proteome dataset (540 interactions) compared to TCGA (83 interactions), BC360 (419 interactions), or CDx (172 interactions). Many of the protein targets identified and drugs screened were clinically relevant to breast cancer and are in clinical trials. Thus, we described the non-redundant knowledge brought by this clone-tailored approach compared to TCGA or transcriptomic panels, the targetable proteins identified in the clonal proteome dataset, and the potential of this approach for drug discovery and repurposing through drug interactions with antineoplastic agents and non-anticancer drugs.

## Highlights

- Spatially resolved mass spectrometry guided by MALDI mass spectrometry imaging allows an in-depth proteomic screening for drug targets in luminal breast cancers.
- This unsupervised and unlabeled technology performed on intact tumors provides a multidimensional analysis of the clonal proteome including conventional proteins, mutated proteins, and alternative proteins.
- The rich clonal proteomic information generated was not redundant with TCGA or transcriptomic panels, and showed pathways exclusively found in the proteomic analysis.
- A large proportion of the proteins in the clonal proteome dataset were druggable with both antineoplastic agents and non-anticancer drugs, showing the potential application to drug repurposing.
- A significant number of the proteins detected had partially or not yet known drug interactions, showing the potential for discovery.
- Many of the protein targets identified and drugs screened were clinically relevant to breast cancer.

## Introduction

Breast cancer remains the most frequent cancer and the leading cause of cancer-related death among women in Europe (globocan iarc). The rational development of targeted drugs based on molecular knowledge of cancer is a major therapeutic progress that brought substantial hope to improving patients’ outcome. However, the complex biological features of this disease, especially the existence of multiple heterogeneous tumor subclones (1), have prevented its eradication, driven drug resistance, including to targeted therapies (2), and has been identified as a marker of poor prognosis in breast cancer patients (3, 4). Integrating tumor heterogeneity in the target discovery process to tailor therapies to the clones present within the tumor is a paradigm shift to reach clonal theranostics. However, technological limitations and breast tumors molecular features have prevented this breakthrough. In fact, this implies the ability to isolate and screen tumor clones separately to understand their biology, find vulnerabilities and identify potential druggable targets.

Historically, sequencing methods revealed genomic alterations driving the emergence of clonal cancer cell subpopulations (5, 6). Beside this genomic heterogeneity, non-genetic mechanisms, such as dynamic transcriptional, translational and metabolic adaptations also contribute to tumor heterogeneity and drug resistance or tolerance (7, 8). Thus, beside the technologies used to detect gene mutations or single nucleotide polymorphisms, technics exploring transcript expression (9), proteins (10), or metabolites (11) also showed significant tumor heterogeneity, demonstrating that heterogeneity is constantly present from the structural to the functional levels of the tumor. Therefore, approaches complementary to genomics are necessary to comprehensively analyze tumor heterogeneity.

Yielding large molecular information on tumor clones from small samples for biomarker or drug target discovery represents a technical challenge despite the advent of single cell technologies (12). Current single-cell sequencing methods require suspensions of cells for isolation, whereas in routine clinical practice the majority of tumors after surgery or biopsy are fixed in formalin and embedded in paraffin blocks. Moreover, analyzing isolated cells does not capture cell-cell interaction in the microenvironment. Spatial transcriptomics represent a powerful tool to access *in situ* functional information about tumor subpopulations (13), and offers the possibility to be multiplexed to fluorescence *in situ* hybridization (14, 15). However, some limitations include the poor prediction of protein expression from RNA expression (16) or transcriptional errors (17) that may hamper drug target inference. Moreover, transcriptome measurements may not necessarily capture adaptive responses that involve post-transcriptional mechanisms such as translation or metabolic reprogramming (18–20). Focusing on tumor proteomic landscape has the advantage of recapitulating both the expressed genomic landscape and the non-genetic processes. This could be of particular interest in tumors with a relatively low mutational burden such as breast cancers (21). Besides, given that the vast majority of drug targets are proteins (22), a proteomic approach allows direct target detection. Technologies specifically dedicated to study the spatial proteomic heterogeneity of tumors, combined or not with transcriptomics are scarce. Most rely on selected and labeled markers, limited in number, for instance with multiplexed pathology methods (23–25), which is not suited for discovery.

We asked whether matrix-assisted laser desorption/ionization (MALDI) mass spectrometry imaging (MSI) combined with microproteomics could screen for relevant druggable protein targets from breast cancer clones to guide clonal theranostics. MALDI MSI enables the spatially resolved label-free imaging of different molecular classes, including proteins, in their histological context (26–28), thus revealing functionally heterogeneous tumor subpopulations in solid tumors (29, 30). The selected subclones are further extracted *in situ* using a semi-automated standardized microproteomic technology to perform a full proteomic profiling with LC-MS/MS (31) comprising identification of referenced proteins but also proteins presenting mutations or alternative proteins issued from the non-coding parts of RNA or non-coding RNA. This approach constitutes a unique tool to characterize the proteomic profile of functionally distinct tumor subpopulations, which we denoted the clonal proteome. Our aims were (i) to map and characterize luminal breast cancers’ functional clones using MALDI MSI combined with microproteomics, and (ii) determine the potential of this approach to identify clinically relevant druggable protein targets in luminal tumors.

## Methods

### Patient Samples and Consent

We carried out a retrospective single center study at Centre Oscar Lambret (Lille, France) to analyze the spatial heterogeneity of primary breast tumors and breast cancer metastases. Eligible patients were women with early breast cancer or metastatic luminal breast cancer with available FFPE tumor tissue after a surgical procedure or a fine needle biopsy. Our pathologists selected 52 primary tumors and 24 metastases from 51 and 12 patients respectively. All patients still alive gave their informed consent. This retrospective study was approved by the local institutional clinical research committee. The clinico-pathological data of both patient series were listed in **Table 1** **(** **Supplementary Table 1** **)**.

**Table 1:** Clinico-pathological parameters for the breast cancer patients’ series.

	Early stage BC	Advanced BC
	n=51 pts	n=12 pts
Age (median, range)	55 (29-80)	64 (47-82)
Initial Tumor size		
T1	18	7
T2	29	3
T3	4	1
unknown	_	1
Histology		
ductal	40	7
lobular	7	3
other	4	2
Tumor grade		
1	5	2
2	44	5
3	2	3
unknown	_	2
Initial nodal involvement		
node positive	26	6
node negative	25	6
Metastases		
yes	0	12
no	51	0
Hormone receptors positive	50	10
unknown	1	2
HER2 expression negative	50	9
unknown	1	3
Metastatic sites at diagnosis of metastases		
node	_	2
liver	_	3
bone	_	6
skin	_	4
lung/pleura	_	4

BC, breast cancer; pts, patients.

### MALDI Mass Spectrometry Imaging

For each tumor sample, 2 consecutive sections of 8 micrometers were cut of off the block. The first section was used to perform the MALDI MSI analysis (27, 32–34). The tumor tissue section was deposited on ITO-coated glass slides (LaserBio Labs, Valbonne, France) and vacuum-dried during 15 min. Protein demasking was performed with washing with NH4HCO3 10mM for 5 min twice, then TRIS HCl 20mM pH9 for 30 min at 95°C. Tryptic digestion was performed (40μg/mL, dissolved in NH4HCO3 50mM) by micro-spraying trypsin on the section surface using an HTX TM sprayer (HTX technologies, LLC), and incubation overnight at 56°C. The slide was dried in a dessicator prior to deposition of a solid ionic matrix HCCA-aniline using an HTX TM sprayer (HTX technologies, LLC). Briefly, 36 μL of aniline were added to 5 mL of a solution of 10 mg/mL HCCA dissolved in ACN/0.1% TFA aqueous (7:3, v/v). A real-time control of the deposition was performed by monitoring scattered light to obtain a uniform layer of matrix. The MALDI mass spectrometry images were performed on a RapifleX Tissuetyper MALDI TOF/TOF instrument (Bruker Daltonics, Germany) equipped with a smartbeam 3D laser. The MSI mass spectra were acquired in the positive delayed extraction reflectron mode using the 500-3000 m/z range, and averaged from 200 laser shots per pixel, using a 70μm spatial resolution raster.

### MALDI MSI Data Processing and Analysis

The MALDI-MSI data were analyzed using SCiLS Lab software (SCiLS Lab 2019, SCiLS GmbH). Common processing methods for MALDI MSI were applied with a baseline removal using a convolution method and data were normalized using Total Ion Count (TIC) method (35, 36). Then, the resulting pre-processing data were clustered to obtain a spatial segmentation using the bisecting k means algorithm (37). Different spatial segmentations were performed. First, an individual segmentation was applied to each tissue separately. Then, the data from all tissues were clustered together to obtain a global segmentation. Briefly, the spatial segmentation consists of grouping all spectra according to their similarity using a clustering algorithm that apply a color code to all pixels of a same cluster. Colors are arbitrarily assigned to clusters; several disconnected regions can have the same color if they share the same molecular content. To limit the pixel-to-pixel variability, edge-preserving image denoising was applied. The segmentation results were represented on a dendrogram resulting from a hierarchical clustering. The branches of the dendrogram were defined based on a distance calculation between each cluster. The manual selection of different branches of the dendrogram allows further segmentation of selected clusters to visualize more regions with distinct molecular composition. Each color-coded region identified a proteomic tumor clone. The regions/clones of interest were then subjected to on-tissue microproteomics, i.e. microdigestion and microextraction, to perform nanoLC-MS & MS/MS analysis of the extract for in-depth protein identification.

### Microproteomic Analysis

Superimposing the molecular image with the immunochemistry image allowed selection of the subclonal areas to be submitted to microproteomics using the second consecutive 8 μm tumor sections. The tissue sections were deposited on polylysine glass slides, and microdigested with a trypsin solution deposited with a microspotter. On-tissue trypsin digestion was performed using a Chemical Inkjet Printer (CHIP-100, Shimadzu, Kyoto, Japan). The trypsin solution (40µg/mL, 50mM NH4HCO3 buffer) was deposited on a region defined to 1mm² for 2h. During this time, the trypsin was changed every half-hour. With 350 cycles and 450pl per spot, a total of 6.3µg was deposited. After microdigestion, the spot content was micropextracted by liquid microjunction using the TriVersa Nanomate (Advion Biosciences Inc., Ithaca, NY, USA) using Liquid Extraction and Surface Analysis (LESA) settings. With 3 different solvent mixtures composed of 0.1% TFA, ACN/0.1% TFA (8:2, v/v), and MeOH/0.1% TFA (7:3, v/v). A complete LESA sequence run 2 cycles for each mixture composed of an aspiration (2µL), a mixing onto the tissue, and a dispensing into low-binding tubes. For each tumor area of interest, 2 microextraction sequences were run and pooled (38).

### NanoLC-MS and MS/MS Analysis

After liquid extraction, samples were freeze-dried in a SpeedVac concentrator (SPD131DPA, ThermoScientific, Waltham, Massachusetts, USA), reconstituted with 10µL 0.1% TFA and subjected to solid-phase extraction to remove salts and concentrate the peptides. This was done using a C-18 Ziptip (Millipore, Saint-Quentin-en-Yvelines, France), eluted with ACN/0.1% TFA (8:2, v/v) and then the samples were dried for storage. Before analysis, samples were suspended in 20µL ACN/0.1% FA (2:98, v/v), deposited in vials and 10µL were injected for analysis. The separation prior to the MS used online reversed-phase chromatography coupled with a Proxeon Easy-nLC-1000 system (Thermo Scientific) equipped with an Acclaim PepMap trap column (75 μm ID x 2 cm, Thermo Scientific) and C18 packed tip Acclaim PepMap RSLC column (75 μm ID x 50 cm, Thermo Scientific). Peptides were separated using an increasing amount of acetonitrile (5%-40% over 140 minutes) and a flow rate of 300 nL/min. The LC eluent was electrosprayed directly from the analytical column and a voltage of 2 kV was applied *via* the liquid junction of the nanospray source. The chromatography system was coupled to a Thermo Scientific Q-Exactive mass spectrometer. The mass spectrometer was programmed to acquire in a data-dependent mode. The survey scans were acquired in the Orbitrap mass analyzer operated at 70,000 (FWHM) resolving power. A mass range of 200 to 2000 m/z and a target of 3E6 ions were used for the survey scans. Precursors observed with an intensity over 500 counts were selected “on the fly” for ion trap collision-induced dissociation (CID) fragmentation with an isolation window of 4 amu and a normalized collision energy of 30%. A target of 5000 ions and a maximum injection time of 120 ms were used for CID MS2 spectra. The method was set to analyze the top 10 most intense ions from the survey scan and a dynamic exclusion was enabled for 20 s. Extracts were sequenced randomly to avoid batch effect.

### Data Analysis

All MS data were processed with MaxQuant (39, 40) (Version 1.5.6.5) using the Andromeda (41) search engine. The proteins were identified by searching MS and MS/MS data against the Decoy version of the complete proteome for Homo sapiens in the UniProt database (Release March 2017, 70941 entries) combined with 262 commonly detected contaminants. Trypsin specificity was used for digestion mode, with N-terminal acetylation and methionine oxidation selected as a variable. We allowed up to two missed cleavages. Initial mass accuracy of 6 ppm was selected for MS spectra, and the MS/MS tolerance was set to 20 ppm for the HCD data. False discovery rate (FDR) at the peptide spectrum matches (PSM) and protein level was set to 1%. Relative, label-free quantification of the proteins was conducted into MaxQuant using the MaxLFQ algorithm (42) with default parameters. Analysis of the identified proteins was performed using Perseus software (http://www.perseus-framework.org/) (version 1.6.12.0). The file containing the information from the identification was filtered to remove hits from the reverse database, proteins with only modified peptides and potential contaminants. The LFQ intensity was logarithmized (log2[x]). Categorical annotation of the rows was used to define the different groups. Principal component analysis (PCA) was done to compare the protein content of each sample. Multiple-sample tests were performed using ANOVA with a p-value of 1%. Normalization was achieved using a Z-score with matrix access by rows. Only proteins that were significant by ANOVA were retained. The hierarchical clustering and profile plots of the statistically significant proteins were performed and visualized with Perseus. Functional annotation and characterization of the identified proteins were performed using FunRich software (version 3) and STRING (version 9.1, http://stringdb.org) (43). Pearson’s correlation coefficient and matrix representation were generated in R software using corrplot package. Gene Set Enrichment Analysis (GSEA) and Cytoscape software (version 3.6.1) were used for the biological process analysis of the clusters selected from the heatmap. The data sets were deposited at the ProteomeXchange Consortium (http://proteomecentral.proteomexchange.org) *via* the PRIDE partner repository (44) with Data available *via* ProteomeXchange with identifier PXD024134.

### Subnetwork Enrichment Pathway Analyses and Statistical Testing

The Elsevier’s Pathway Studio version 10.0 (Ariadne Genomics/Elsevier) was used to deduce relationships among differentially expressed proteomics protein candidates using the Ariadne ResNet database (45, 46). “Subnetwork Enrichment Analysis” (SNEA) algorithm was selected to extract statistically significant altered biological and functional pathways pertaining to each identified set of protein hits among the different groups. SNEA utilizes Fisher’s statistical test set to determine if there are nonrandom associations between two categorical variables organized by specific relationships. Integrated Venn diagram analysis was performed using “the InteractiVenn”: a web-based tool for the analysis of complex data sets (47). Annotation analysis of gene ontology terms for the identified proteins was performed using PANTHER Classification System (version 15.0, http://www.pantherdb.org) (48). Interaction network analyses were performed with Cytoscape (version 3.7.2) and the Cluego application (version 2.5.5) to interpret the lists of genes and proteins by selecting representative Gene Ontology terms and pathways from multiple ontologies and visualize them into functionally organized networks (49). The ontologies used included GO_BiologicalProcess-EBI-UniProt-GOA_27.02.2019, GO_CellularComponent-EBI-UniProt-GOA_27.02.2019, GO_ImmuneSystemProcess-EBI-UniProt-GOA_27.02.2019, GO_MolecularFunction-EBI-UniProt-GOA_27.02.2019, KEGG_27.02.2019, REACTOME_Pathways_27.02.2019, REACTOME_Reactions_27.02.2019, and WikiPathways_27.02.2019. The GO level range was 3 to 8, and groups with more than 50% overlap were merged. The statistical test used was enrichment/depletion (two-sided hypergeometric test) with a Bonferroni step down correction method.

### Mutated Protein Identification

Protein identification was also performed using the mutation-specific database (50). XMan v2 database contains 2 539 031 mutated peptide sequences from 17 599 Homo sapiens proteins (2 377 103 are missense and 161 928 are nonsense mutations). The interrogation was performed by Proteome Discoverer 2.3 software and Sequest HT package, using an iterative method. The precursor mass tolerance was set to 15 ppm and the fragment mass tolerance was set to 0.02 Da. For high confidence result, the FDR values were specified to 1%. A filter with a minimum Xcorr of 2 was applied. The generated result file was filtered using a Python script to remove unmutated peptides. All mutations were then manually checked based on MS/MS spectra profile.

### Alternative Proteins Identification

RAW data obtained by nanoLC-MS/MS analysis were processed using Proteome Discoverer V2.3 (Thermo Scientific) with the following parameters: Trypsin as an enzyme, 2 missed cleavages, methionine oxidation as a variable modification, Precursor Mass Tolerance: 10 ppm and Fragment mass tolerance: 0.6 Da. The validation was performed using Percolator with an FDR set to 0.001%. A consensus workflow was then applied for the statistical arrangement, using the high confidence protein identification. The protein database was uploaded from Openprot (https://openprot.org/) and included reference proteins, novel isoforms, and alternative proteins predicted from both Ensembl and RefSeq annotations (GRCh38.83, GRCh38.p7) (51).

### TCGA, BC360, CDx Datasets

To compare our proteomic data with genomic and transcriptomic datasets used in breast cancer, the reference genes of the proteins were contrasted to publically available TCGA, BC360 (nanoString^®^) and CDx (Foundation One^®^) datasets. Breast cancer genomic alterations were collected from the TCGA web portal using “breast cancer” as keyword in the search engine. The TCGA gene list is in **Supplementary Material 1**. The gene list of the BC360 and CDx panels were obtained from nanoString and Foundation One websites. The gene lists are in **Supplementary Materials 2** and **3** respectively.

### Druggable Genome Database

DrugCentral (http://drugcentral.org) is an online drug information resource created and maintained by the Division of Translational Informatics at University of New Mexico in collaboration with the IDG Illuminating the Druggable Genome (IDG) (https://druggablegenome.net/index) (52). DrugCentral provides information on active ingredients, chemical entities, pharmaceutical products, the mode of action of drugs, indications, and pharmacologic action. Data is monitored on FDA, EMA, and PMDA for new drug approval on regular basis. Supported target search terms are HUGO gene symbols, Uniprot accessions and target names, and Swissprot identifiers. We used the WHO anatomical therapeutic chemical (ATC) classification to categorize drugs.

### Druggability Level of the Targets

The druggability level of the targets was classified using the definition of the Illuminating the Druggable Genome Knowledge Management Center (IDG-KMC) based on four target development levels (TDLs) categorized as follows: (i) Tclin: targets with activities in DrugCentral (i.e., approved drugs) and known mechanism of action, (ii) Tchem: targets with activities in ChEMBL or DrugCentral that satisfy the activity thresholds detailed in https://druggablegenome.net/ProteinFam, (iii) Tbio: targets with no known drug or small molecule activities that satisfy the activity thresholds and criteria (detailed in https://druggablegenome.net/ProteinFam), (iv) Tdark: targets with virtually no known drug or small molecule activities that satisfy the criteria defined by IDG-KMC.

### Clinical Trial Database

ClinicalTrials.gov is a web-based resource that provides information on publicly and privately supported clinical studies on a wide range of diseases and conditions (https://clinicaltrials.gov/ct2/home). The web site is maintained by the National Library of Medicine at the National Institutes of Health (US). Information on ClinicalTrials.gov is provided and updated by the sponsor or principal investigator of the clinical study. 6159 studies corresponding to interventional clinical trials conducted in breast cancer patients were retrieved using “breast cancer” as the medical condition and “drug” as other condition. Results were also filtered to select “Adult” and “Older Adult”, and “Interventional Studies”.

### Statistical Analyses

Descriptive analyses used frequency of distribution, median, quartiles and extremes. Survival analyses were performed using the breast cancer Kaplan-Meier plotter online tool (https://kmplot.com; n=3955). The database sources included GEO, EGA, and TCGA (53). A multiple gene testing was run using available cohorts of patients with estrogen receptor positive and HER2 negative disease to analyze the reference genes association with distant metastases free survival (DMFS) and overall survival (OS). A logrank p<0.05 was considered significant.

## Results

### Workflow for Breast Cancer Clonal Proteome Analysis

**Figure 1:**
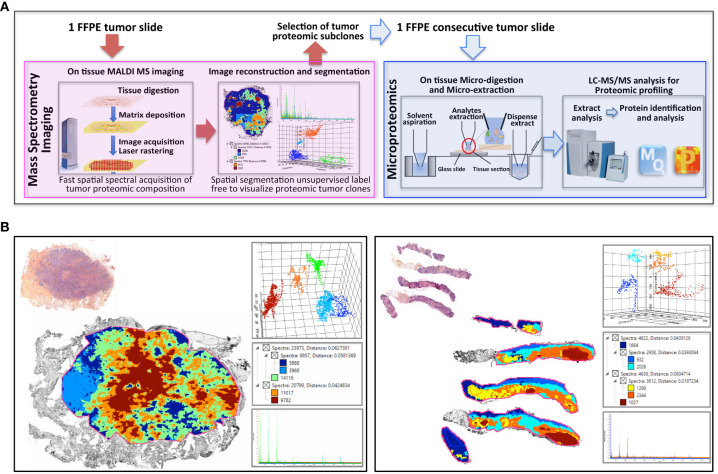
Clonal proteome analysis in breast cancer. **(A)** Workflow for on tissue analysis of tumor proteomic heterogeneity using spatially resolved microproteomics guided by MALDI MSI **(B)** The presence of tumor proteomic clones revealed by MSI was illustrated in a primary tumor (left) from a surgical resection (case 42) and a metastatic sample (right) collected with a fine needle biopsy (case 22). In each sample vignette, the MALDI MS imaging is displayed with the histological HPS picture (upper left), the principal component analysis of the proteomic clones (upper right), the segmentation tree (middle right), and the spectra of the clones (bottom right).

**Figure 2:**
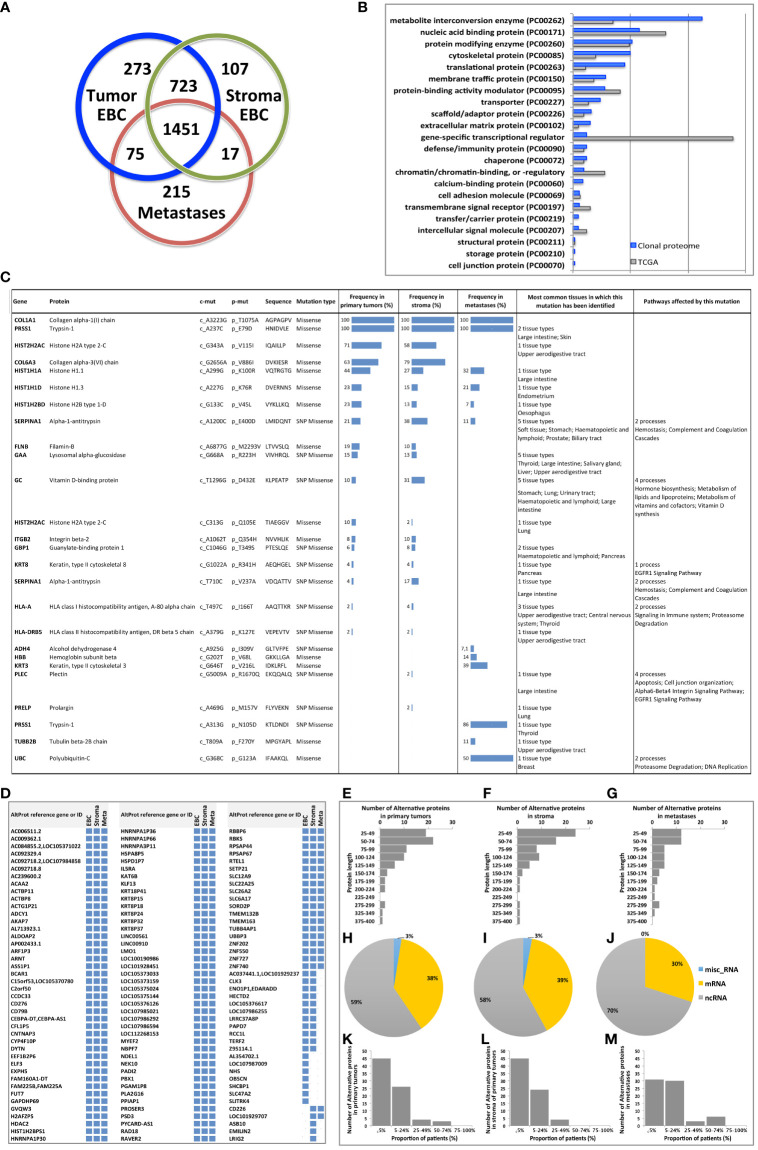
**(A)** The Venn diagram showing the number of proteins specific or shared among primary tumors (blue), stroma (green) and metastases (red). **(B)** Distribution of protein classes (in %) in the clonal proteome dataset (in blue) compared with TCGA dataset (in grey). **(C)** Mutated proteins identified using mass spectrometry, with their frequency in primary tumors, stroma and metastases, and the tissues in which they have been reported, along with the processes they affected. **(D)** 126 Alternative proteins identified by mass spectrometry; their length is indicated in **(E)** primary tumor samples, **(F)** stroma samples and **(G)** metastases. Their coding RNA distribution is shown in **(H–J)**, respectively. The frequency of alternative proteins among patients is shown in **(K)** primary tumors, **(L)** stroma and **(M)** metastases. AltProt, alternative proteins; EBC, early breast cancer; ID, identification; Meta, metastases; SNP, single nucleotide polymorphism.

The workflow described in **Figure 1A** was applied to two FFPE tumor slides to provide a spatially resolved unsupervised and unlabeled visualization of breast cancer spatial heterogeneity, and an in-depth proteomic profiling. The MALDI MSI on-tissue spatial analysis mapped high molecular weight peptide composition on the first tumor slide. The spectral data obtained were clustered by the bisecting k-means method, which attributed color-coded groups to tumors areas according to the similarity of their proteomic signature. Manual group splitting (group segmentation) was limited to 3 in order to map only main functional differences between tumor subpopulations. Imaging revealed distinct proteomic clones, as illustrated in **Figure 1B** showing representative MALDI MS images of a primary tumor and a metastasis sample among the 76 luminal tumors analyzed (52 primary breast tumors and 24 metastases in (**Supplementary Materials 4**, **5** **)**. From the MSI data, 124 MSI clonal areas were retrieved corresponding to 52 areas of primary tumors, 48 areas of primary tumor stroma and 24 areas of metastases. Each of these 124 MSI clones were individually analyzed by spatially resolved shotgun proteomic. MaxLFQ algorithm was used to perform label-free quantification of proteins and resulted in a total of 2868 proteins from the 124 clonal areas (**Figure 2A**). The number of proteins identified did not significantly differ between tissue types **(** **Supplementary Material 6A** **)**, or according to the sampling method, *i.e.* mastectomy, surgical biopsy or fine needle core biopsy **(** **Supplementary Material 6B** **)**. Panther analysis showed that the main protein classes found in the clonal proteome dataset were enzymes, cytoskeletal proteins, membrane-traffic, translational or scaffold proteins, or transporters (**Figure 2B**). As a comparison, gene-specific transcriptional regulators, chromatin related proteins or transmembrane signal receptor were more abundant in the TCGA dataset (**Figure 2B**). Differences between primary tumors and stroma, or between primary tumors and metastases were mild among the main protein classes (**Supplementary Material 7**). We also detected modified proteins, specifically mutated proteins and alternative proteins. Mutations-missense at a protein-level were identified by expanding the search of the raw mass spectrometry files of our proteomic dataset against a mutated peptide database. The search identified 26 mutated proteins, 18 in primary tumors, 20 in stroma and 12 in metastases, with various frequencies (**Figure 2C**). Similarly, expanding the search to alternative proteins databases retrieved 126 alternative proteins in the clonal proteome dataset (**Figure 2D** **)**: 79 were identified in primary tumors, 69 in stroma and 50 in metastases. The majority of these alternative proteins had a length ranging from 29 to 150 amino acids (**Figures 2E–G** **)**. They were coded mainly from non-coding RNA (**Figures 2H–J**). These proteins were infrequent and found mostly in less than 25% of the patients (**Figures 2K–M**).

### Luminal Tumors Clonal Proteome Landscape Among Classic and Modified Proteins

**Figure 3:**
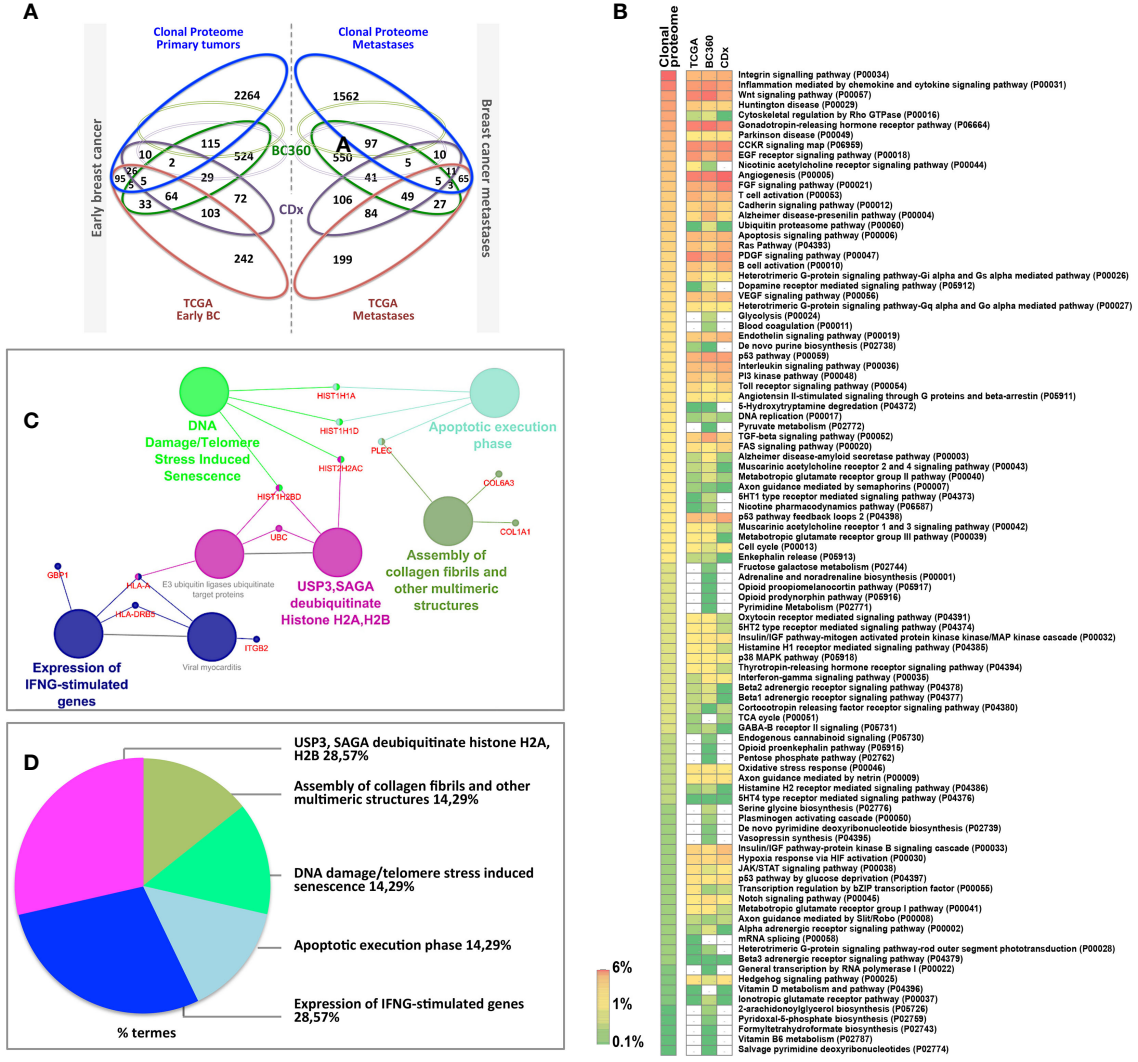
**(A)** Venn diagram comparing the clonal proteome dataset with TCGA, BC360 and CDx panels, in primary breast cancer (left) or metastases (right). **(B)** Panther pathways heatmap showing gene distribution between the datasets. Pathways over-represented are colored in orange and those underrepresented in green. **(C)** Mutated protein networks and **(D)** biological processes distribution analyzed using Cytoscape and Cluego.

To fully understand the molecular information brought by our approach and its relevance to drug target discovery, the clonal proteomic dataset was further compared to the TCGA breast cancer database and two transcriptomic panels, BC360 (nanoString^®^) and CDx (Foundation One^®^). The Venn diagram in **Figure 3A** showed that only few proteins of the clonal proteomic dataset (identified by their reference gene) were shared with TCGA, BC360 or CDx panels, both in primary tumors and metastases. 2264 and 1562 proteins were exclusively found in the clonal proteome dataset of primary tumors and metastases respectively (**Supplementary Material 8**). Enrichment analysis using Panther software identified 139 pathways in the clonal proteome dataset. Only 55% of these pathways were also present in the TCGA dataset, 68% in BC360 and 50% in CDx. The pathways and processes identified were differentially distributed across the datasets as depicted in the heatmap in **Figure 3B**. Pathways over-represented in the clonal proteome dataset were integrin or inflammation mediated by chemokine and cytokine signaling pathways, cytoskeletal regulation by Rho GTPase, the ubiquitin proteasome pathway, glycolysis, *de novo* purine biosynthesis or DNA replication. Under-represented processes were mainly signaling pathways or the oxidative stress response. 41 pathways were exclusive to the clonal proteome (**Table 2**), mainly metabolic pathways involved in amino acid, lipid or nucleic acid synthesis. Seven of these pathways have been suggested as candidate for drug targeting, 22 have been associated with breast cancer in experimental or clinical reports, the remaining 19 pathways have been understudied in breast cancer (**Table 2**) (54–86). The mutated proteins identified have been reported in a variety of human tissues; only mutated polyubiquitin-C has been reported in breast tissue with an impact on proteasome degradation and DNA replication. The other mutated proteins may affect hemostasis, complement and coagulation cascades, hormone biosynthesis, metabolism, EGFR1 signaling, signaling in the immune system, apoptosis, cell junction organization, or integrin signaling (**Figure 2C**). Enrichment analyses performed on the identified mutated proteins using their reference genes showed three main biological processes: expression of interferon gamma genes, apoptosis, and senescence (**Figures 3C, D**). Only 4 of the reference genes (COL6A3, COL1A1, HBB, HLA-A) were also found in TCGA or BC360 datasets (none in CDx). The functions of the alternative proteins identified are not known yet. Among their known reference genes, only 8 (ARNT, CD79B, KAT6B, LMO1, PBX1, CD276, ELF3, HDAC2) were also present in TCGA, BC360 or CDx panels.

**Table 2:** Pathways exclusive to the clonal proteomic dataset.

Pathway exclusive to the clonal proteomic dataset	Panther pathway ID	Proteins (gene ID)	Involvement in BC and potentially druggable	Reference
5HT3 type receptor mediated signaling pathway	P04375	SNAP23, VAT1	_	
Acetate utilization	P02722	ACSS3	Nutrient	(54)
Adenine and hypoxanthine salvage pathway	P02723	HPRT1, ADA	_	(55)
Alanine biosynthesis	P02724	BCAT2	Alters cell migration and proliferation; sensitivity to doxorubicin	(56)
Aminobutyrate degradation	P02726	ALDH5A1, ABAT	Connection to p53/apoptosis pathway; chemotherapeutic efficacy of doxorubicin	(57)
Androgen/estrogen/progesterone biosynthesis	P02727	ACAT1, ACAT2, HSD17B6	Tumor growth*	(58)
Arginine biosynthesis	P02728	ASL, CPS1, CAD, ASS1	Metabolic starvation therapy; estrogen signaling connection*	(59)
Asparagine and aspartate biosynthesis	P02730	GOT2, GOT1	_	
ATP synthesis	P02721	CYC1, HAO1	oncosphere formation; regulation of cancer driver genes	(60)
Cholesterol biosynthesis	P00014	HMGCS1	Cancer stem cell propagation; mechanism of resistance to endocrine therapy*	(61–63)
Coenzyme A biosynthesis	P02736	PPCS, PANK4	_	
Cysteine biosynthesis	P02737	CBS	_	
*De novo* pyrimidine ribonucleotides biosynthesis	P02740	CPS1, CTPS2, CTPS1, NME2, CAD	Metabolic reprogramming; synthetic lethality with DNA damaging chemotherapy	(64)
Gamma-aminobutyric acid synthesis	P04384	ALDH5A1, ABAT	Hormonal regulation and BC pathogenesis	(65)
General transcription regulation	P00023		Inflammatory BC	(66)
Glutamine glutamate conversion	P02745	GLUD1	Cell growth; mTOR connection; stress response pathway*	(67)
Heme biosynthesis	P02746	EPRS, FECH, CPOX, HMBS, ALAD, QARS	Cancer stem cells mammosphere formation	(68)
Histidine biosynthesis	P02747	TAT	_	
Isoleucine biosynthesis	P02748	BCAT2, ILVBL	_	
Leucine biosynthesis	P02749	IDH3B, BCAT2	_	
Mannose metabolism	P02752	GMPPB, PMM2, GMDS, GMPPA	_	
Methionine biosynthesis	P02753	CTH	Altered methylation	(69)
Methylcitrate cycle	P02754	ACSS3, ACO1	_	
Methylmalonyl pathway	P02755	PCCB, MCCC2	_	
N-acetylglucosamine metabolism	P02756	GNPDA2, GNPDA1, GFPT1	DNA repair regulation; tumorigenesis; metabolic reprograming; survival stress signaling; epigenetics*	(70–78)
Nicotine degradation	P05914	FMO3, CYP2A6, UGT2B7	_	
O-antigen biosynthesis	P02757	GFPT1, MAT2B	_	
Ornithine degradation	P02758	ALDH16A1	Synthetic lethality*	(79)
Phenylethylamine degradation	P02766	ALDH16A1, AOC3	_	
Proline biosynthesis	P02768	PYCR1	Proline biosynthesis activated in ER negative tumors*	(80)
Purine metabolism	P02769	MTAP	BC cell lines differentiation; pathway genetic interactions	(81, 82)
Pyridoxal phosphate salvage pathway	P02770	PNPO	_	
S-adenosylmethionine biosynthesis	P02773	MAT2A, MAT1A	Cancer stem cells*	(83)
Salvage pyrimidine ribonucleotides	P02775	NME2	_	
Succinate to proprionate conversion	P02777	PCCB, ECHDC1, ECHS1, MCCC2	_	
Sulfate assimilation	P02778	PAPSS2, PAPSS1	_	
Synaptic vesicle trafficking	P05734	UNC13D, NSF	Intercellular communication	(84)
Threonine biosynthesis	P02781		BC cell lines differentiation	(82)
Tyrosine biosynthesis	P02784	TAT	Differentially expressed in BC cancer	(85)
Valine biosynthesis	P02785	BCAT2, ILVBL	Associated with BC subtypes	(86)
Xanthine and guanine salvage pathway	P02788	HPRT1	_	

*Pathways potentially druggable.

### Clonal Proteome Druggability and Interactions With Approved Drugs

**Figure 4:**
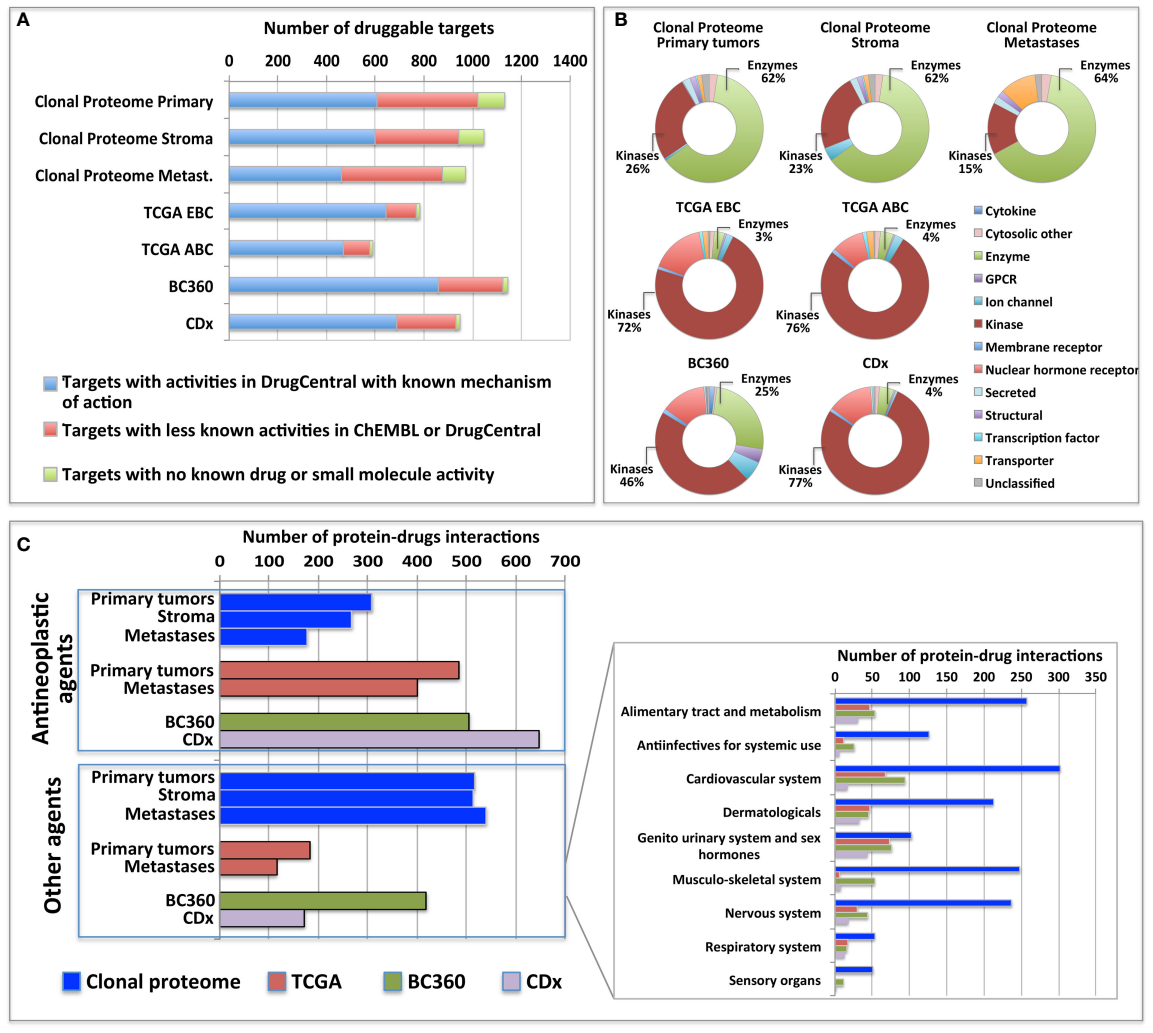
Druggable targets identified **(A)** in the clonal proteome, TCGA, CDx, and BC360 datasets using DrugCentral, and the druggability level as defined by IDG-KMC (https://druggablegenome.net/ProteinFam). Known targets (Tclin) are in blue, less known targets are in orange (Tchem) and targets with no known drug are in red (Tbio). **(B)** Target class distribution among the datasets, and **(C)** matching drugs, both approved antineoplastic drugs or other drugs (non-anticancer drugs) described using the ATC classification. ABC, advanced breast cancer; EBC, early breast cancer; Metas, metastases.

The clonal proteome dataset was reviewed against DrugCentral database to determine the number of proteins targetable, their level of druggability and their interaction with approved drugs. Among the proteins identified in the clonal proteome dataset, 1495 proteins were targetable with a level of druggability high for 52% of them (known mechanism of action and drug interaction, Tclin), while 39% had a lower level of knowledge (Tchem), and 9% had no known drug or small molecule interaction (Tbio) (**Figure 4A**). The highest number of druggable targets was observed in the clonal proteome compared to the genomic and transcriptomic datasets. The proportion of less known targets was also greater in the clonal proteome dataset (47%) compared to TCGA (18%), BC360 (23%) or CDx (25%) (**Figure 4A**). The main target classes in the clonal proteome dataset were enzymes (60%), kinases (23%) and transporters (7%), whereas kinases were dominant in the other datasets (46% to 77%) (**Figure 4B**). The number of protein and drug interactions with antineoplastic and immunomodulating agents were up to 309 in the clonal proteome dataset, 485 in TCGA, 506 in BC360, and 647 in CDx (**Figure 4C** and **Supplementary Material 9**). Among the anticancer drugs, 35 drugs matched uniquely with the clonal proteome dataset, with only 7 of them already approved in breast cancer. The number of target and drug interactions with non-anticancer drugs (such as agents targeting the cardiovascular system, metabolism, the musculoskeletal or the nervous systems) was higher in the clonal proteome dataset (540 interactions) compared to TCGA (83 interactions), BC360 (419 interactions), or CDx (172 interactions) (**Figure 4C**).

### Proteins and Processes of Interest in the Clonal Proteome Dataset for Target Discovery

**Figure 5:**
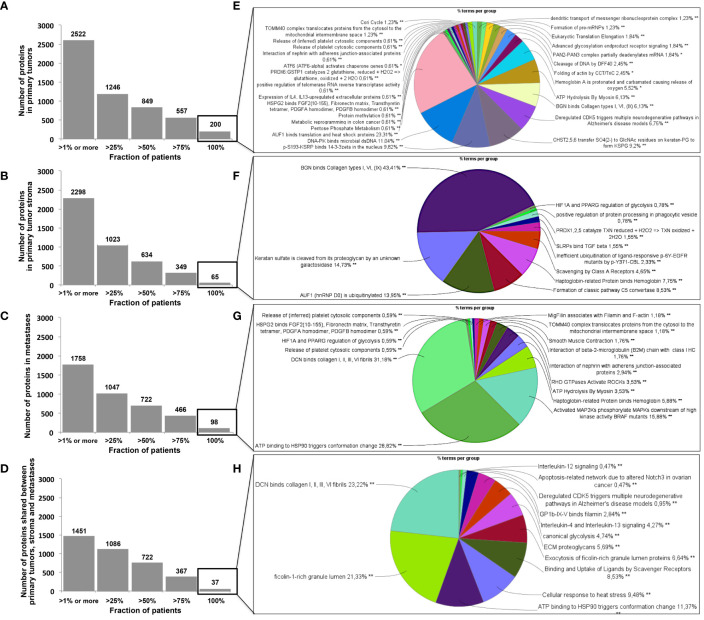
Distribution of proteins among patients in **(A)** primary tumors, **(B)** in stroma, **(C)** in metastases, and **(D)** shared in all samples. Biological processes enriched (in %) from proteins shared by all patients in **(A–D)** are represented as pie charts in **(E–H)**, respectively. Analyses were performed with Cytoscape and ClueGo. *p < 0.05, **p < 0.01, ***p < 0.001.

**Figure 6:**
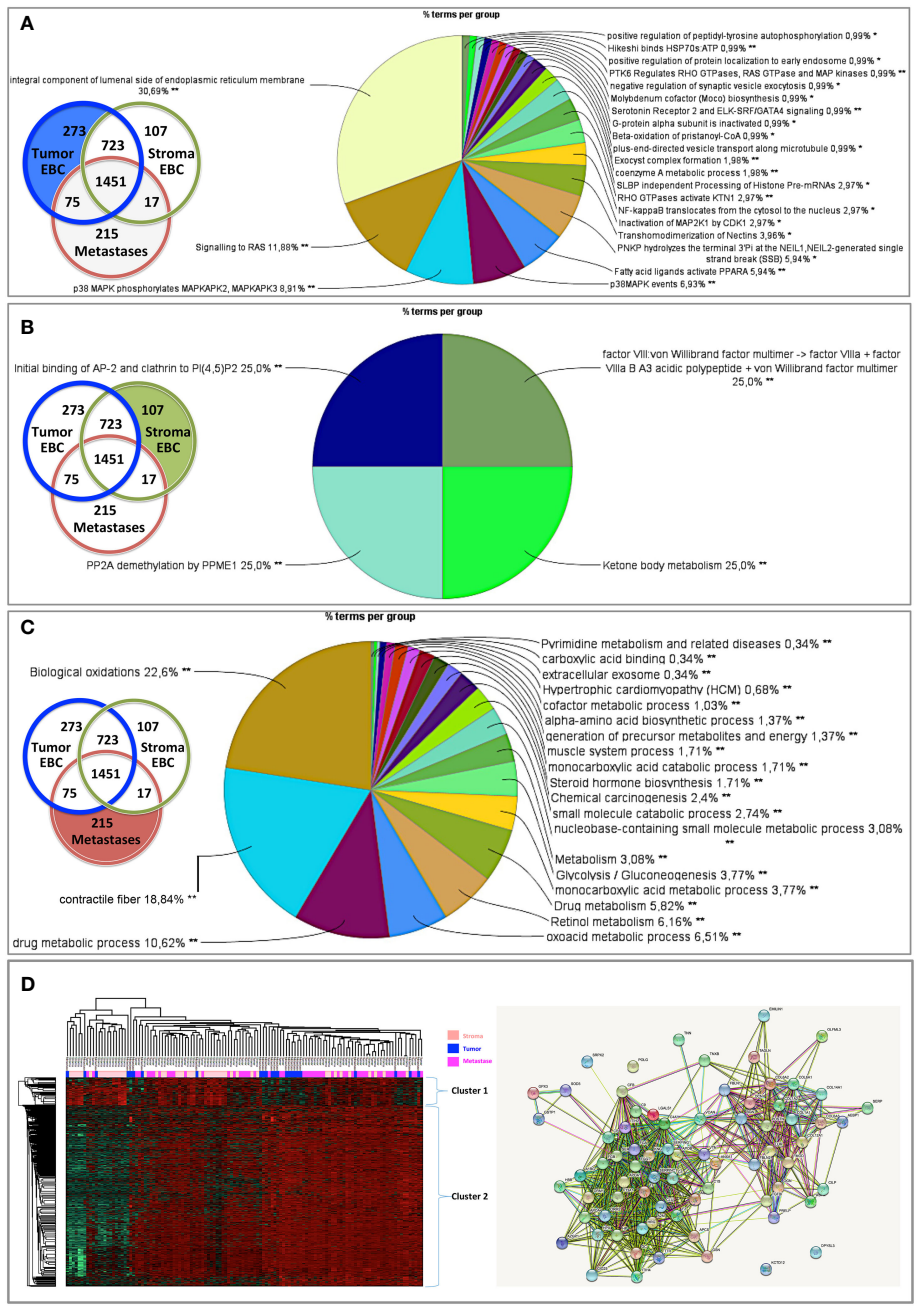
Biological processes enriched from proteins specifically found in **(A)** primary tumors, **(B)** stroma, or **(C)** metastases. Proteins differentially expressed in **(D)** primary tumors, stroma and metastases were analyzed using a multiple sample test ANOVA with a p<0.01 and represented in a heat map (on the left) identifying 2 clusters separating the stroma (Cluster 1) from the primary tumor and the metastases (Cluster 2). The String networks of the clusters are shown on the bottom right Analyses were performed with Cytoscape, ClueGo and String; proportions of processes in %. *p<0.05; **p<0.01.

In the clonal proteome dataset, proteins shared among samples or specific to primary tumors, stroma or metastases, or differentially expressed may associate with biological processes intrinsic to breast cancer stage, tumor microenvironment, or progression. These proteins may therefore be of interest, especially if they also associate with breast cancer survival. Protein distribution among patients showed that 200 proteins were found in all primary tumor samples (**Figure 5A**), 65 proteins were shared among all the stromal samples (**Figure 5B**), 98 proteins were found in all the metastases samples **(** **Figure 5C** **)**, and 37 proteins were present in all the 124 clonal samples (**Figure 5D**). Enrichment analyses for specific pathways showed as main biological processes in primary tumors AUF1, DNA-PK, S193-KSRP, or CDK5 related activities (**Figure 5E**), in stroma BGN activity, keratin sulfate cleavage, AUF1 ubiquitinoylation, and C5 pathway activity (**Figure 5F**), and in metastases DCN, HSP90, and MAP2Ks related activities (**Figure 5G**). In addition, ficolin-rich granule exocytosis and cellular response to heat stress were among the main processes found in all samples (**Figure 5H**). Enrichment analyses of proteins specific to primary tumors (n=273), stroma (n=107) or metastases (n=215) showed biological processes related to membrane components of the endoplasmic reticulum, RAS or MAPK signaling in primary tumors (**Figure 6A**), AP-2, clathrin, or PP2A activities or ketone body metabolism in stroma (**Figure 6B**), oxidation, contractile fibers processes, and drug metabolism in metastases (**Figure 6C**).

**Figure 7:**
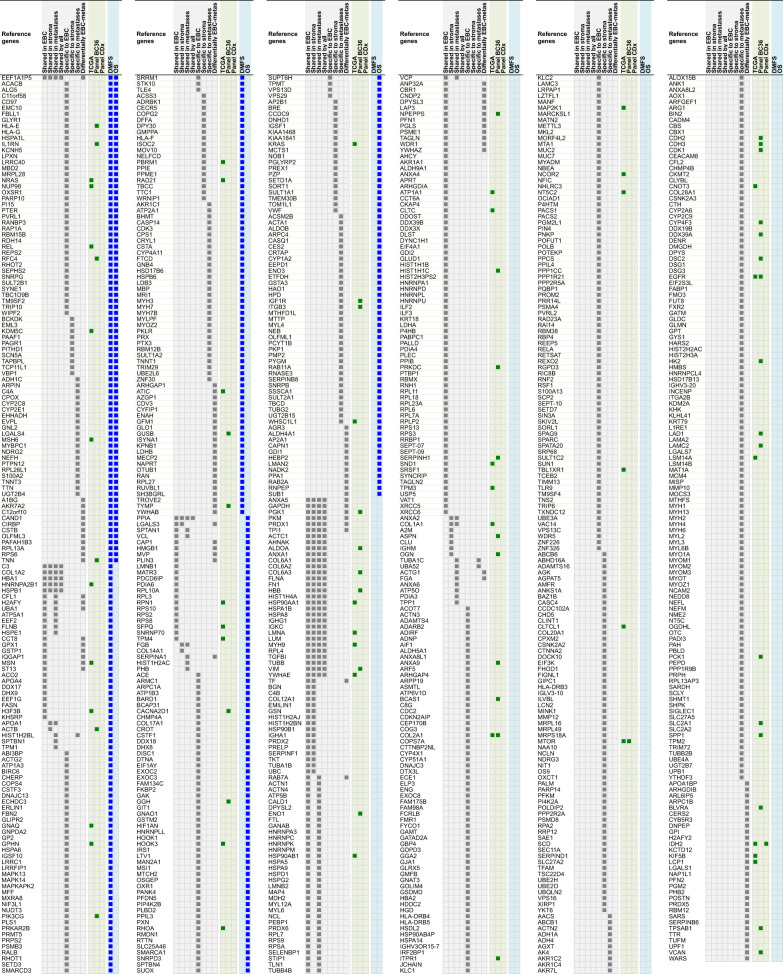
Proteins shared, specific or differentially expressed in the clonal proteome dataset (in grey). 892 proteins are indicated with their reference gene. Proteins also found in TCGA, BC360 or CDx datasets are indicated in green. Genes associated with DMFS or OS using publically available databases are shown in blue. EBC, early breast cancer; DMFS, distant metastases free survival; OS, overall survival.

**Figure 8:**
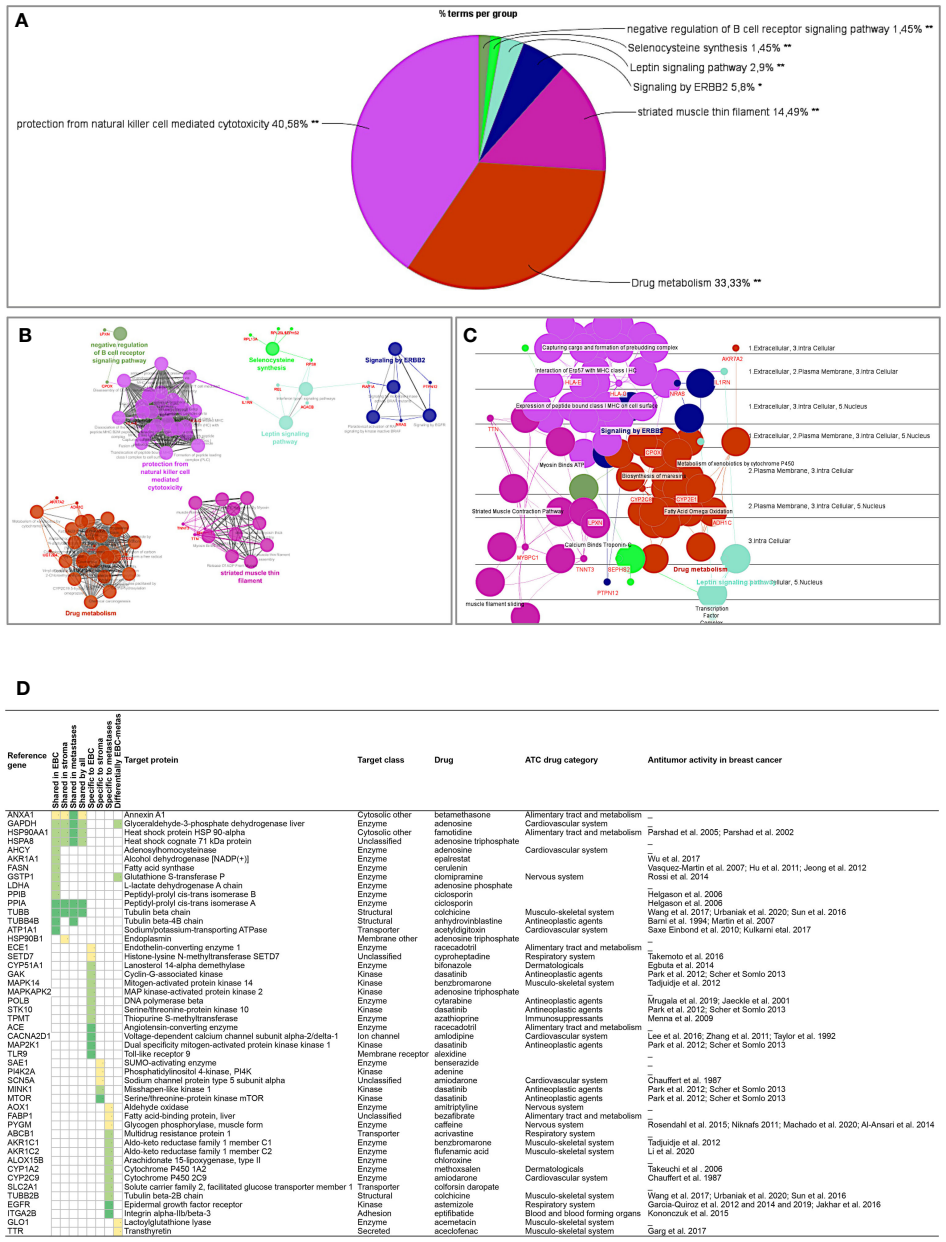
Analyses of the proteins from the clonal proteome associated both with DMFS and OS, showing **(A)** enrichment, **(B)** networks analysis, **(C)** cerebral layout of cellular distribution, and **(D)** their druggability. In the table, targets with a known drug-target interaction are in dark green, those with a less known interaction are in green, and those with limited data are in yellow. The target class, matching drug name, ATC classification and reported antitumor activity in breast cancer are indicated. *p < 0.05; **p < 0.01.

**Figure 9:**
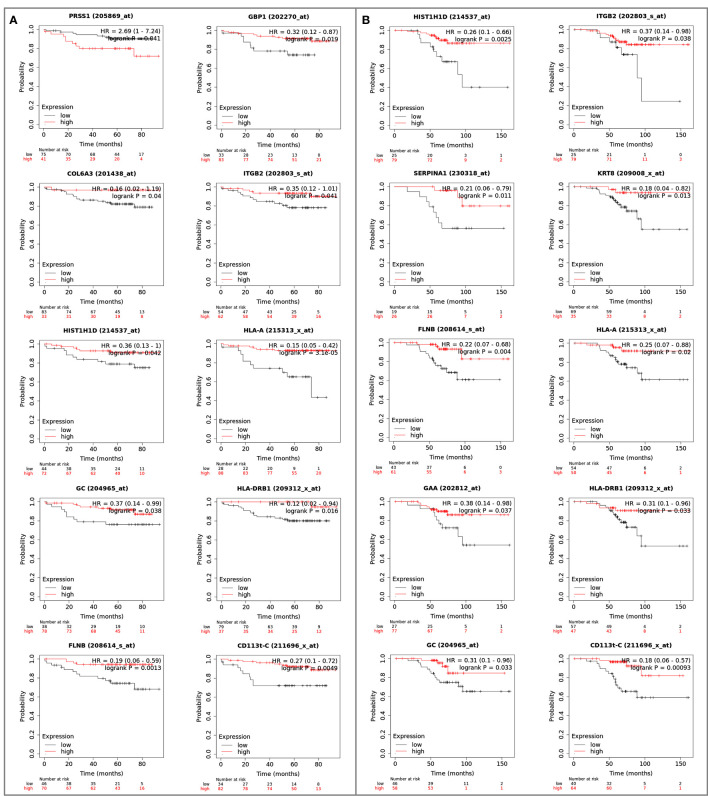
Reference genes of the mutated proteins identified in the clonal proteome dataset associated with breast cancer **(A)** DMFS or **(B)** OS. The breast cancer Kaplan-Meier plotter tool was used to run multiple reference gene testing in publically available estrogen receptor positive and HER2 negative cohorts. A logrank p<0.05 was considered significant. DMFS, distant metastases free survival; HR, hazard ratio; OS, overall survival.

Proteins differentially expressed among primary tumors, stroma and metastases were identified using a multiple sample test ANOVA with a p<0.01. A total of 662 proteins showed a significant difference in expression among the 3 groups. Two clusters have been identified separating the stroma (Cluster 1) from the primary tumor and the metastases (Cluster 2) (**Figure 6D**). String analysis of Cluster 1 revealed two separated networks linked by VCAN *i.e.* one centered on immune response inhibition and one on collagen proteins and protein in interaction with the extracellular matrix (**Supplementary Material 10**). In Cluster 2, gene ontology reflects the presence of paraspeckles, VCP-NSFL1C complex, cytosolic small ribosomal subunit, cytosolic and polysomal ribosome, SNP and RNP complexes networks. KEGG Pathways identify as major networks the ones related to metabolism (lipids, glycosgelysis, pyruvate, proteins, carbon, butanoate, amino acid residues), antigen processing and presentation (**Supplementary Material 10**). The relationship between the proteins of interest in the clonal proteome (shared, specific or differentially expressed) with TCGA, BC360 or CDx datasets on the one hand, and with survival on the other hand was detailed in **Figure 7**. Less than 5% of these proteins of interest were shared with the genomic/transcriptomic datasets, and 25% were associated with distant metastases free survival (DMFS) (n=222) or overall survival (OS) (n=227). Enrichment analyses of genes associated with both breast cancer DMFS and OS showed their involvement in natural killer cell mediated cytotoxicity, drug metabolism, muscle filament, ERBB2 and leptin signaling, aminoacid synthesis and B cell receptor signaling (**Figures 8A**–**C**). Among the proteins of interest, 48 (5%) had interactions with known drugs, mostly non-anticancer agents such as colchicine, acemetacin, aceclofenac (musculo-skeletal system), astemizole (respiratory system), eptifibatide (blood system), or acetyldigitoxin (cardiovascular system), which have shown anti-tumor activity experimentally in breast cancer (**Figure 8D**) (**Supplementary Table 2**). Among the mutated proteins, 10 reference genes were associated with breast cancer DMFS (**Figure 9A**) or OS (**Figure 9B**) and were involved in the response to interferon gamma.

### Clinical Relevance of the Drugs Identified With the Clonal Proteome Approach

To show the clinical usefulness of the drugs identified through their interactions with protein targets of the clonal proteomic dataset, the clinicaltrial.gov database was searched to determine the proportion of these drugs that reached clinical investigation for breast cancer treatment. Among the 721 drugs accessible through the clonal proteome, 107 drugs were investigated in at least one breast cancer clinical trial: only 26 were drugs already used to treat breast cancer, 49 were antineoplastic drugs not yet approved for breast cancer and 32 were non-anticancer drugs. As expected, the 26 drugs used in breast cancer were chemotherapy molecules (taxanes, doxorubicin, eribulin, methotrexate, gemcitabine, etoposide, platinum, vinorelbine), endocrine therapy, targeted therapies such as everolimus and olaparib, and anti-HER2 therapies (trastuzumab, pertuzumab, trastuzumab emtansine, lapatinib), and steroids and zoledronic acid. Our approach identified 49 antineoplastic drugs already under clinical investigation to explore their value for repositioning in breast cancer, either in monotherapy or in combination. Their known protein targets were detailed in **Table 3** along with identification number of the clinical trials and the trials phase. A majority of these drugs were investigated in phase 1 or 2 trials. Our approach also identified 32 non-anticancer drugs in trial for repurposing in breast cancer. The drugs were mainly anti-infective agents, or were involved in metabolism, the cardiovascular system or the nervous system. The ATC category of the drugs was indicated in **Table 4** with the protein targets, trials ID and trial phase.

**Table 3:** Anticancer drugs identified through the clonal proteome that are under clinical investigation in breast cancer patients for repositioning.

Drug name	Protein targets	Reference genes	Trial status	Phase 1 trials	Phase 1/2 trials	Phase 2 trials	Phase 3 trials or other
afatinib	Cyclin-G-associated kinase Serine/threonine-protein kinase 10 STE20-like serine/threonine-protein kinase Mitogen-activated protein kinase 14	GAK STK10 SLK MAPK14	Completed	NCT01649271	NCT01441596; NCT01594177		
			Ongoing	NCT03878524	NCT02768337	NCT04158947; NCT02465060	
apatinib	Tyrosine-protein kinase CSK	CSK	Completed			NCT01176669; NCT01653561; NCT02878057; NCT03394287	
			Ongoing	NCT03075462		NCT02768415; NCT03254654; NCT03775928; NCT03982485; NCT04303741; NCT03580395	NCT04335006; NCT03475589
belinostat	Histone deacetylase 1,2,3,4,5,6,7,8,9,10,11	HDAC1,2,3,4,5,6,7,8,9,10,11	Completed	NCT00413322			
			Ongoing	NCT04315233; NCT04703920			
bendamustine	Histone deacetylase 1,2,3,6,8,10	HDAC1,2,3,6,8,10	Completed	NCT00661739	NCT00834678	NCT01891227	

bortezomib	Proteasome subunit alpha type-1 Prothrombin Cathepsin G Chymase Proteasome subunit beta type-1/-5/-2 Nuclear factor NF-kappa-B complex 26S proteasome non-ATPase regulatory subunit 1	PSMA1 F2 CTSG CMA1 PSMB1/2/5 NFKB1 PSMD1	Completed	NCT00620295; NCT00622674; NCT00667641		NCT00025584; NCT00028639	
			Ongoing	NCT03878524		NCT01142401	
bosutinib	ALK tyrosine kinase receptor Angiopoietin-1 receptor Bcr/Abl fusion protein Dual specificity mitogen-activated protein kinase kinase 1/2 Ephrin type-A receptor 2 Epidermal growth factor receptor Hepatocyte growth factor receptor Histone deacetylase 1,2,3,4,5,6,7,8,9,10,11 Macrophage colony-stimulating factor 1 receptor Mast/stem cell growth factor receptor Kit Non-receptor tyrosine-protein kinase TYK2 Platelet-derived growth factor receptor alpha/beta Protein kinase C delta type Proto-oncogene tyrosine-protein kinase receptor Ret Proto-oncogene tyrosine-protein kinase Src Receptor tyrosine-protein kinase erbB-2/-4 Receptor-type tyrosine-protein kinase FLT3 Rho-associated protein kinase 1/2 Tyrosine-protein kinase ABL1, BTK, FYN, HCK, ITK, JAK2, JAK3, LCK, LYN, YES1	ALK TEK ABL1 MAP2K1/2 EPHA2 EGFR MET HDAC1,2,3,4,5,6,7,8,9,10,11 CSF1R KIT TYK2 PDGFRA/B PRKCD RET SRC ERBB2/4 FLT3 ROCK1/2 ABL1, BTK, FYN, HCK, ITK, JAK2, JAK3, LCK, LYN, YES1	Completed	NCT00759837			

Their known protein targets are indicated along with the identification number of the clinical trials (NCT number) and the trials phase.

**Table 4:** Non-anticancer drugs identified through the clonal proteome that are under clinical investigation in breast cancer patients for repurposing.

Drug ATC class	Drug name	Protein targets	Reference genes	Trial status	Phase 1 trials	Phase 2 trials	Phase 3/4 or other
Alimentary tract and metabolism	calcitriol	Vitamin D3 receptor Vitamin D 25-hydroxylase 25-hydroxyvitamin D-1 alpha hydroxylase, mitochondrial Vitamin D-binding protein	VDR CYP2R1 CYP27B1 GC	Ongoing		NCT01293682	
Alimentary tract and metabolism	doxycycline	72 kDa type IV collagenase	MMP2	Completed		NCT01847976	
				Ongoing	NCT03435952	NCT02874430	
Alimentary tract and metabolism	lansoprazole	Microtubule-associated protein tau	MAPT	Ongoing		NCT04188119	
Alimentary tract and metabolism	omeprazole	Cytochrome P450 1A2 Cytochrome P450 2C9 Multidrug resistance protein 1	CYP1A2 CYP2C9 ABCB1	Completed	NCT01596647		
				Ongoing	NCT02950259	NCT02595372	
Alimentary tract and metabolism	sulfasalazine	Caspase-1 Mitogen-activated protein kinase 1 Acetyl-CoA acetyltransferase, mitochondrial Carbonic anhydrase 1, 2 Cyclooxygenase	CASP1 MAPK1 ACAT1 CA1, CA2 PTGS1	Ongoing		NCT03847311	
Antiinfectives for systemic use	itraconazole	Lanosterol 14-alpha demethylase	CYP51A1	Completed			NCT00798135
				Ongoing	NCT04712396		
Antiinfectives for systemic use	ritonavir	Cytochrome P450 2C9 Multidrug resistance protein 1	CYP2C9 ABCB1	Completed	NCT01009437		
Antiparasitic products, insecticides and repellents	hydroxychloroquine	Toll-like receptor 9	TLR9	Ongoing	NCT03774472	NCT03032406; NCT04523857	
Antiparasitic products, insecticides and repellents	suramin	DNA-dependent protein kinase catalytic subunit Protein arginine N-methyltransferase 1	PRKDC PRMT1	Completed	NCT00003038; NCT00054028		
Blood and blood forming organs	apixaban	Prothrombin Coagulation factor X	F2 F10	Completed	NCT03083782		
Cardiovascular system	amlodipine	Voltage-dependent L-type calcium channel subunit alpha-1D/-1C Voltage-dependent calcium channel gamma-1 subunit Alpha-2A adrenergic receptor 5-hydroxytryptamine receptor 6 Alpha-2C adrenergic receptor Sodium-dependent dopamine transporter Carbonic anhydrase 1 Aldehyde oxidase Voltage-dependent T-type calcium channel subunit alpha-1H Alpha-1D adrenergic receptor Potassium channel subfamily K member 2 Alpha-1A adrenergic receptor Alpha-1B adrenergic receptor Voltage-dependent calcium channel subunit alpha-2/delta-1	CACNA1D/C CACNG1 ADRA2A HTR6 ADRA2C SLC6A3 CA1 AOX1 CACNA1H ADRA1D KCNK2 ADRA1A ADRA1B CACNA2D1	Ongoing	NCT02834403		
Cardiovascular system	atorvastatin	3-hydroxy-3-methylglutaryl-coenzyme A reductase Nuclear receptor subfamily 1 group I member 3 Cytochrome P450 3A4 Histone deacetylase 1,2,6	HMGCR NR1I3 CYP3A4 HDAC1,2,6	Completed		NCT00816244	
Cardiovascular system				Ongoing	NCT01980823	NCT03872388	
Cardiovascular system	digoxin	Sodium/potassium-transporting ATPase Signal transducer and activator of transcription 3	ATP1A1 STAT3	Completed	NCT00650910; NCT04094519	NCT01763931	
				Ongoing	NCT03928210		
Cardiovascular system	indomethacin	Prostaglandin G/H synthase 1 Aldo-keto reductase family 1 member C4/C2 Lactoylglutathione lyase Multidrug resistance protein 1	PTGS1 AKR1C4/AKR1C2 GLO1 ABCB1	Ongoing	NCT02950259		
Cardiovascular system	lidocaine	Sodium channel protein type 5 subunit alpha	SCN5A	Completed		NCT02839668	
Cardiovascular system	losartan	Cytochrome P450 2C9 Angiotensin-converting enzyme Multidrug resistance protein 1	CYP2C9 ACE ABCB1	Ongoing	NCT03878524		
Cardiovascular system	propranolol	Cytochrome P450 1A2 Multidrug resistance protein 1	CYP1A2 ABCB1	Ongoing		NCT01847001	
Dermatologicals	tacrolimus	Peptidyl-prolyl cis-trans isomerase FKBP1A Peptidyl-prolyl cis-trans isomerase FKBP10 Serine/threonine-protein kinase mTOR Splicing factor 3B subunit 3 Serine/threonine-protein phosphatase 2B catalytic subunit alpha isoform Peptidyl-prolyl cis-trans isomerase FKBP5	FKBP1A FKBP10 MTOR SF3B3 PPP3CA FKBP5	Completed	NCT03083782		
Dermatologicals	tretinoin	Mitogen-activated protein kinase 1	MAPK1	Ongoing	NCT03878524		
Dermatologicals	ketoconazole	Aldehyde oxidase Lanosterol 14-alpha demethylase Multidrug resistance protein 1	AOX1 CYP51A1 ABCB1	Ongoing	NCT03796273		
Genito urinary system and sex hormones	celecoxib	Carbonic anhydrase 2 Prostaglandin G/H synthase 1 Carbonic anhydrase 1/9 Mitogen-activated protein kinase 14	CA2 PTGS1 CA1 - CA9 MAPK14	Completed	NCT00070057; NCT01425476	NCT00006381; NCT00056082; NCT00201773; NCT00291694; NCT01695226	NCT00525096; NCT02429427
				Ongoing	NCT01881048; NCT03599453; NCT03878524; NCT04081389	NCT04348747	
Genito urinary system and sex hormones	mifepristone	Mitogen-activated protein kinase 14	MAPK14	Completed	NCT01493310; NCT02046421		NCT02651844
				Ongoing		NCT01898312; NCT02788981; NCT03225547	
Genito urinary system and sex hormones	sildenafil	cGMP-specific 3’,5’-cyclic phosphodiesterase	PDE5A	Completed	NCT01375699		
Musculo-skeletal system	nimesulide	Prostaglandin G/H synthase 1 Myeloperoxidase	PTGS1 MPO	Completed		NCT01500577	
Musculo-skeletal system	sulindac	72 kDa type IV collagenase Lactoylglutathione lyase	MMP2 GLO1	Completed	NCT00245024	NCT00039520	
Nervous system	modafinil	Cytochrome P450 1A2	CYP1A2	Completed			NCT00917748
Nervous system	disulfiram	Cytochrome P450 1A2 Amine oxidase [flavin-containing] A	CYP1A2 MAOA	Ongoing		NCT03323346; NCT04265274	
Nervous system	fluvoxamine	Cytochrome P450 1A2	CYP1A2	Completed	NCT01700270		
Nervous system	midazolam	Multidrug resistance protein 1	ABCB1	Completed	NCT00258050; NCT01596647; NCT03955939		
				Ongoing	NCT01296555; NCT01655225		
Nervous system	pregabalin	Voltage-dependent calcium channel subunit alpha-2/delta-1	CACNA2D1	Ongoing			NCT03216187
Nervous system	propofol	Prostaglandin G/H synthase 1 Cytochrome P450 2C9 Carbonic anhydrase 1 Carbonic anhydrase 2	PTGS1 CYP2C9 CA1 CA2	Completed			NCT02005770; NCT02758249
Nervous system				Ongoing			NCT01975064; NCT04074460
Nervous system	valproic acid	Alcohol dehydrogenase [NADP(+)] Histone deacetylase 2 Succinate-semialdehyde dehydrogenase, mitochondrial Histone deacetylase 1	AKR1A1 HDAC2 ALDH5A1 HDAC1	Ongoing	NCT01552434		

The ATC category is indicated with the protein targets, the identification number of the clinical trials (NCT number) and the trials phase.

## Discussion

The present study showed that MALDI MSI combined with microproteomics can be used as a precision oncology tool to map and profile specific tumor subpopulations in luminal breast cancers for clonal theranostic applications. This unsupervised and label-free technology characterized the tumors conventional proteome along with the mutated and alternative proteomes, at a clonal level, to identify candidate druggable targets. Our MS imaging and microproteomic technology offers the advantage of identifying proteomic clones *in situ* on intact tumors. In MALDI MSI, the signal intensities are recorded for analytes at specific x,y coordinates of the tissue section in their native states. MSI produces images of the scanned area where each pixel contains the MS spectrum at this location. In the present study, 204 spectra were generated by square millimeter. Spectra are high-dimensional vectors (typically in the order of 10^5^ dimensions), making MSI data similar to hyperspectral images. The spectrum produced by MSI at a given location represents a signature of the molecules present at this location. This proteomic signature was used to provide a label free unbiased method to map and visualize tumor functional heterogeneity and perform directed proteomic profiling on selected tumor subpopulations. This label free method is a strength compared to multiplex technics that require few selected markers (23). This makes the MSI-microproteomics technology particularly suited to discovery. So far, relevant candidate tumor targets for drug development are sought mainly among tumor genomic alterations because of their putative role as oncogenic drivers and in drug resistance. Implementation of this concept in the clinic has yielded mitigated benefit for patients (87). Moreover, the majority of oncogenic mutations are not druggable (88). Target inference from bioinformatic analyses based on genomic data may not provide knowledge precise enough about the functional state of the tumor and its diversity to identify relevant targets. Searching candidate targets among proteins circumvents these limitations. The performance and usefulness of the MSI-microproteomic technology was previously reported in solid tumors such as ovarian cancer or gliomas to help finding novel biomarkers or refining diagnosis classification (29, 30). Additionally, MSI-microproteomics tumor subpopulation scale allowed a successful identification of specific tumor stroma proteins. This is a significant advantage given the involvement of the tumor microenvironment in drug response (88), contrary to single cell methods, which cannot analyze intercellular communications in their intact microenvironment.

The clonal proteome showed a rich landscape of proteins and biological processes compared to genomic or transcriptomic datasets. The overlap with TCGA data and transcriptomic panels was limited and a distinct distribution of biological processes was observed in enrichment analyses. The clonal proteomic dataset provided more information on enzymatic and metabolic processes. A study by Patel et al. reporting on a computational assessment of drug targets showed that enzymes were the most frequent protein class and the most druggable (89). The clonal proteome revealed 41 exclusive metabolic pathways, most of them understudied in relation to breast cancer. This was of particular interest because tumor metabolic phenotype has been recognized as a hallmark of cancer and is involved in drug resistance (81). Transcriptomic panels were enriched with kinases or immune processes as expected. The discrepancy with TCGA data may be related to alternative splicing and post-translational modifications revealed by mass spectrometry analyses that cannot be predicted by genome databases. Although the bulk of targetable proteins identified might not be involved in driver oncogenic pathways to which cancer cells are addicted, focusing on common and cell type- or stage-specific proteins and processes might increase their relevance. The relevance of the protein targets was showed by the fact that a large number of proteins in the clonal proteome dataset was associated with breast cancer outcome and highlighted shared or specific biological processes of interest.

An additional strength of the present mass spectrometry technology relies on the detection of altered proteins, such as those originating from missense mutations or single nucleotide polymorphism missense mutations, and a newly recognized type of proteins named alternative proteins (or ghost proteins) because of their translation from alternative open reading frames. Although their functions cannot be predicted from their reference gene, their presence may reveal altered biological processes. Alternative proteins represent a vast class of proteins with still largely unknown biological functions, thus expanding the proteome complexity (90). This field of research offers exciting perspectives about the functions of these modified proteins related to cancer and their potential impact on drug target interactions.

Our study showed that a clonal proteomic analysis brought additional non-redundant molecular information. The proteins and pathways uniquely identified with this clonal approach may offer opportunities to identify novel drug targets. Drug development struggles with the molecular heterogeneity of tumor subpopulations, potentially leading to a differential target expression among cancer cells, which contributes to drug resistance. This has stimulated the development of multi-targeted therapeutic strategies (91, 92), facilitated by the fast expansion of the drug pipeline. Interestingly, a high proportion of the proteins in the clonal proteome dataset were druggable, with interactions with a variety of drug classes, either antineoplastic agents or non-anticancer drugs. We showed that many of these drugs, both antineoplastic agents and non-anticancer drugs, were already under clinical investigation for breast cancer treatment. This underlines the clinical relevance of using this approach for clone-tailored strategies of systematic high-throughput unbiased drug target screening for drug combination or repositioning (93). A significant number of proteins had partially or not yet known drug interactions, showing also the potential of our approach for discovery. Despite the recognition of breast cancer heterogeneity, technical limitations hampered the implementation of clonal theranostics in practice. MSI-microproteomics technology revealed more edges of breast cancer heterogeneity and bridges the technological gap to allow contemplating a paradigm shift from treating one main detectable tumor clone (with current technics) to strategies taking into account several functional clones. To tackle tumor complexity, system biology approaches are developing to reveal therapeutic opportunities associated with the multiple dimensions of cancer through integration of tumor genome, phenome and other omics data (94, 95). Accessing sufficient quantities of tumor tissue to perform all the omics analyses represents a technical challenge. Our technology uses only a limited amount of tumor tissue while maintaining the whole tissue section integrity allowing it to be re-used for additional experiments. For this reason and the large amount of data generated, the MSI-microproteomic technology is suited to multiomic strategies.

In conclusion, spatially resolved MSI-guided microproteomics is a unique tool to perform a label-free multidimensional proteomic characterization of intratumor heterogeneity for clone-tailored drug target screening. This new approach is adapted to drug target discovery and repurposing to achieve clonal theranostics. Moreover, it is applicable in routine clinical care and its scalability thanks to the speed of analysis of current and next generation mass spectrometry instruments, makes MSI-microproteomics integration to precision oncology tools foreseeable in a near future to implement clone-tailored therapies.

## Data Availability Statement

The datasets presented in this study can be found in online repositories. The names of the repository/repositories and accession number(s) can be found in the article/**Supplementary Material**.

## Ethics Statement

The studies involving human participants were reviewed and approved by the local Research and Ethics Committee (Centre Oscar Lambret). The patients/participants provided their written informed consent to participate in this study.

## Author Contributions

NH wrote the manuscript original draft. NH designed the study. SA and NH performed the analyses. NH and DB selected and collected the breast cancer samples. Y-MR and DB performed histology and validated diagnostics. NH, SA, TC, IF, and MS analyzed the data. IF, MS, TC, and SA corrected the manuscript. NH, IF, and MS supervised the project and MS, IF, and NH provided the funding. All authors contributed to the article and approved the submitted version.

## Funding

This work was funded by Inserm and Centre Oscar Lambret.

## Conflict of Interest

The authors declare that the research was conducted in the absence of any commercial or financial relationships that could be construed as a potential conflict of interest.

## Publisher’s Note

All claims expressed in this article are solely those of the authors and do not necessarily represent those of their affiliated organizations, or those of the publisher, the editors and the reviewers. Any product that may be evaluated in this article, or claim that may be made by its manufacturer, is not guaranteed or endorsed by the publisher.

## Supplementary Material

Supplementary Material 1 TCGA database of mutations and CNV alterations in early and advanced breast cancers.

Supplementary Material 2 BC360 panel gene list

Supplementary Material 3 CDx panel gene list

Supplementary Material 4 MALDI MSI of 52 cases of primary tumors showing the spatial proteomic heterogeneity of the tumors. In each sample vignette, the MALDI MS imaging is displayed with the histological HPS picture (upper left), the principal component analysis of the proteomic clones (upper right), the segmentation tree (middle right), and the spectra of the clones (bottom right).

Supplementary Material 5 MALDI MSI of 24 cases of metastases showing the spatial proteomic heterogeneity of the tumors. In each sample vignette, the MALDI MS imaging is displayed with the histological HPS picture (upper left), the principal component analysis of the proteomic clones (upper right), the segmentation tree (middle right), and the spectra of the clones (bottom right).

Supplementary Material 6 Number of total proteins identified according to **(A)** tissue types (bone n=3; liver n=5; nodes n=6; skin n=10) or **(B)** to the sampling method **(B)**, i.e. surgery (n=52), surgical biopsy (n=9) or fine needle core biopsy (n=15). Line in the middle of the box is the median, the upper and lower sides of the box represent the 75th and 25th percentiles respectively, and the outside lines are the extremes.

Supplementary Material 7 Panther analysis of the clonal proteome landscape showing **(A)** the protein class distribution (in %), and **(B)** a comparison of the distribution between primary tumors and stroma (relative difference in % in blue) and between primary tumors and metastases (relative difference in % in red).

Supplementary Material 8 Reference gene distribution across the four datasets (clonal proteome, TCGA, BC360, CDx).

Supplementary Material 9 Targets interacting with anticancer drugs across the four datasets (clonal proteome, TCGA, BC360, CDx).

Supplementary Material 10 String networks in stroma (cluster 1) and in primary tumors and metastases (cluster 2).

Supplementary Table 1 Information concerning patients and type of tumors identified by the pathologist.

Supplementary Table 2 References cited in **Table 2** and **Figure 8D**.

## Abbreviations

ABC, advanced breast cancer; ACN, acetonitrile; AGC, automatic gain control; AltProt, alternative proteins; Amu, atomic mass unit; ANOVA, analysis of variance; ATC, anatomical therapeutic chemical; BC, breast cancer; Da, Dalton; DMFS, distant metastases free survival; EBC, early breast cancer; EMA, European medicines agency; FDA, food and drug administration; FDR, false discovery rate; FFPE, formalin-fixed paraffin-embedded; HCCA, hydroxycinnamic acid; HER2, human epidermal growth factor receptor-2; HR, hazard ratio; HUGO, human genome organization; ID, identification; IDG, illuminating the druggable genome; IDG-KMC, illuminating the druggable genome knowledge management center; ITO, indium tin oxide; LC, liquid chromatography; LESA, liquid extraction surface analysis; LFQ, label-free quantification; MALDI, matrix assisted laser desorption ionization; MeOH, methanol; Meta metastases; MS mass spectrometry; MSI mass spectrometry imaging; OS, overall survival; PMDA, pharmaceuticals and medical devices agency; PSM, peptide spectrum matches; SNP, single nucleotide polymorphism; TDL, target development level; TFA, trifluoroacetic acid; TIC, total ion count.
