# Supplementary material for: Path to Clonal Theranostics in Luminal Breast Cancers
Source: Front Oncol. 2022 Jan 13;11:802177. doi: 10.3389/fonc.2021.802177 (PMC8793283; doi:10.3389/fonc.2021.802177)
Supplement: Supplementary Material 1 — TCGA database of mutations and CNV alterations in early and advanced breast cancers. [file DataSheet_1.zip › Data Sheet 10.pdf]

Supplementary material 10: String networks in stroma (cluster 1) and in primary tumors and metastases (cluster 2)

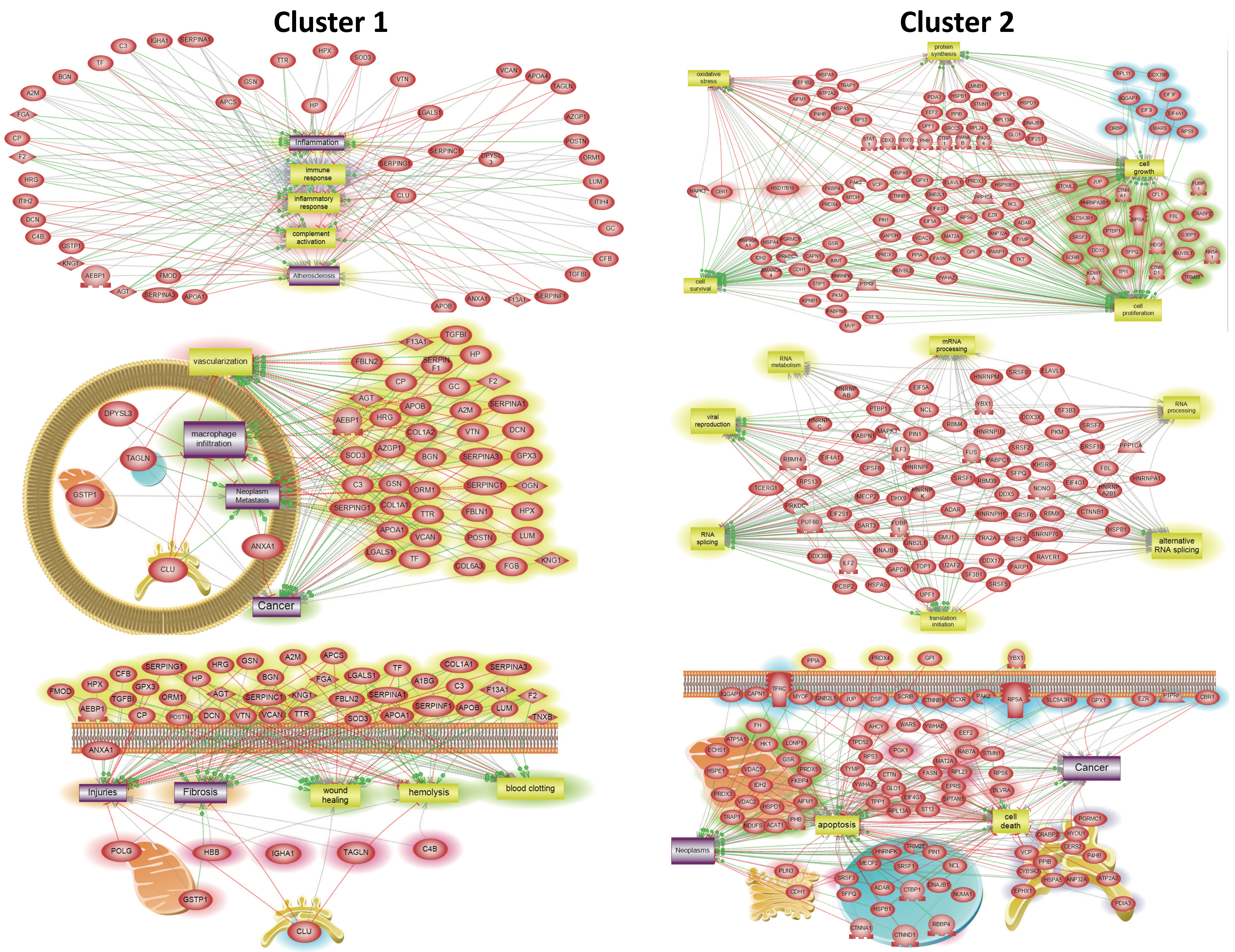

Cluster 1
Cluster 2
Inflammation
immune response
inflammatory response
complement activation
Atherosclerosis
vascularization
macrophage infiltration
Neoplasm Metastasis
Cancer
Injuries
Fibrosis
wound healing
hemolysis
blood clotting
protein synthesis
oxidative stress
cell growth
cell survival
cell proliferation
mRNA processing
RNA metabolism
RNA processing
viral reproduction
RNA splicing
alternative RNA splicing
translation initiation
apoptosis
cell death
Neoplasms
Cancer
