# Supplementary material for: Path to Clonal Theranostics in Luminal Breast Cancers
Source: Front Oncol. 2022 Jan 13;11:802177. doi: 10.3389/fonc.2021.802177 (PMC8793283; doi:10.3389/fonc.2021.802177)
Supplement: Supplementary Material 1 — TCGA database of mutations and CNV alterations in early and advanced breast cancers. [file DataSheet_1.zip › Data Sheet 12.docx]

**Supplementary Table 2**: References cited in Table 2 and Figure 8D

Al-Ansari MM, Aboussekhra A. Caffeine mediates sustained inactivation of breast cancer-associated myofibroblasts via up-regulation of tumor suppressor genes. PLoS One 2014; 9: e90907.

Barni S, Ardizzoia A, Bernardo G, Villa S, Strada MR, Cazzaniga M et al. Vinorelbine as single agent in pretreated patients with advanced breast cancer. Tumori 1994; 80: 280–282.

Barupal DK, Gao B, Budczies J, Phinney BS, Perroud B, Denkert C et al. Prioritization of metabolic genes as novel therapeutic targets in estrogen-receptor negative breast tumors using multi-omics data and text mining. Oncotarget 2019; 10: 3894–3909.

Brown KK, Spinelli JB, Asara JM, Toker A. Adaptive Reprogramming of De Novo Pyrimidine Synthesis Is a Metabolic Vulnerability in Triple-Negative Breast Cancer. Cancer Discov 2017; 7: 391–399.

Cai D, Wang J, Gao B, Li J, Wu F, Zou JX et al. RORγ is a targetable master regulator of cholesterol biosynthesis in a cancer subtype. Nat Commun 2019; 10: 4621.

Chauffert B, Rey D, Coudert B, Dumas M, Martin F. Amiodarone is more efficient than verapamil in reversing resistance to anthracyclines in tumour cells. Br J Cancer 1987; 56: 119–122.

Chiaradonna F, Ricciardiello F, Palorini R. The Nutrient-Sensing Hexosamine Biosynthetic Pathway as the Hub of Cancer Metabolic Rewiring. Cells 2018; 7. doi:10.3390/cells7060053.

Efimova EV, Takahashi S, Shamsi NA, Wu D, Labay E, Ulanovskaya OA et al. Linking Cancer Metabolism to DNA Repair and Accelerated Senescence. Mol Cancer Res 2016; 14: 173–184.

Egbuta C, Lo J, Ghosh D. Mechanism of inhibition of estrogen biosynthesis by azole fungicides. Endocrinology 2014; 155: 4622–4628.

Ehmsen S, Pedersen MH, Wang G, Terp MG, Arslanagic A, Hood BL et al. Increased Cholesterol Biosynthesis Is a Key Characteristic of Breast Cancer Stem Cells Influencing Patient Outcome. Cell Rep 2019; 27: 3927-3938.e6.

Einbond LS, Wu H-A, Su T, Chang T, Panjikaran M, Wang X et al. Digitoxin activates EGR1 and synergizes with paclitaxel on human breast cancer cells. J Carcinog 2010; 9: 10.

Fan Y, Zhou X, Xia T-S, Chen Z, Li J, Liu Q et al. Human plasma metabolomics for identifying differential metabolites and predicting molecular subtypes of breast cancer. Oncotarget 2016; 7: 9925–9938.

Ferrer CM, Lynch TP, Sodi VL, Falcone JN, Schwab LP, Peacock DL et al. O-GlcNAcylation regulates cancer metabolism and survival stress signaling via regulation of the HIF-1 pathway. Mol Cell 2014; 54: 820–831.

Ferrer CM, Sodi VL, Reginato MJ. O-GlcNAcylation in Cancer Biology: Linking Metabolism and Signaling. J Mol Biol 2016; 428: 3282–3294.

García-Quiroz J, García-Becerra R, Barrera D, Santos N, Avila E, Ordaz-Rosado D et al. Astemizole synergizes calcitriol antiproliferative activity by inhibiting CYP24A1 and upregulating VDR: a novel approach for breast cancer therapy. PLoS One 2012; 7: e45063.

García-Quiroz J, García-Becerra R, Santos-Martínez N, Barrera D, Ordaz-Rosado D, Avila E et al. In vivo dual targeting of the oncogenic Ether-à-go-go-1 potassium channel by calcitriol and astemizole results in enhanced antineoplastic effects in breast tumors. BMC Cancer 2014; 14: 745.

García-Quiroz J, González-González ME, Díaz L, Ordaz-Rosado D, Segovia-Mendoza M, Prado-García H et al. ASTEMIZOLE, AN INHIBITOR OF ETHER-À-GO-GO-1 POTASSIUM CHANNEL, INCREASES THE ACTIVITY OF THE TYROSINE KINASE INHIBITOR GEFITINIB IN BREAST CANCER CELLS. Rev Invest Clin 2019; 71: 186–194.

Garg NK, Tyagi RK, Sharma G, Jain A, Singh B, Jain S et al. Functionalized Lipid-Polymer Hybrid Nanoparticles Mediated Codelivery of Methotrexate and Aceclofenac: A Synergistic Effect in Breast Cancer with Improved Pharmacokinetics Attributes. Mol Pharm 2017; 14: 1883–1897.

Geck RC, Foley JR, Murray Stewart T, Asara JM, Casero RA, Toker A. Inhibition of the polyamine synthesis enzyme ornithine decarboxylase sensitizes triple-negative breast cancer cells to cytotoxic chemotherapy. J Biol Chem 2020; 295: 6263–6277.

Geck RC, Toker A. Nonessential amino acid metabolism in breast cancer. Adv Biol Regul 2016; 62: 11–17.

Helgason HH, Kruijtzer CMF, Huitema ADR, Marcus SG, ten Bokkel Huinink WW, Schot ME et al. Phase II and pharmacological study of oral paclitaxel (Paxoral) plus ciclosporin in anthracycline-pretreated metastatic breast cancer. Br J Cancer 2006; 95: 794–800.

Hu N, Li Y, Zhao Y, Wang Q, You J, Zhang X et al. A novel positive feedback loop involving FASN/p-ERK1/2/5-LOX/LTB4/FASN sustains high growth of breast cancer cells. Acta Pharmacol Sin 2011; 32: 921–929.

Ifergan I, Assaraf YG. Molecular mechanisms of adaptation to folate deficiency. Vitam Horm 2008; 79: 99–143.

Jaeckle KA, Phuphanich S, Bent MJ, Aiken R, Batchelor T, Campbell T et al. Intrathecal treatment of neoplastic meningitis due to breast cancer with a slow-release formulation of cytarabine. Br J Cancer 2001; 84: 157–163.

Jakhar R, Paul S, Bhardwaj M, Kang SC. Astemizole-Histamine induces Beclin-1-independent autophagy by targeting p53-dependent crosstalk between autophagy and apoptosis. Cancer Lett 2016; 372: 89–100.

Jeong NY, Yoo YH. Cerulenin-induced apoptosis is mediated by disrupting the interaction between AIF and hexokinase II. Int J Oncol 2012; 40: 1949–1956.

Kim D-H, Yoon H-J, Cha Y-N, Surh Y-J. Role of heme oxygenase-1 and its reaction product, carbon monoxide, in manifestation of breast cancer stem cell-like properties: Notch-1 as a putative target. Free Radic Res 2018; 52: 1336–1347.

Kim H-Y, Lee K-M, Kim S-H, Kwon Y-J, Chun Y-J, Choi H-K. Comparative metabolic and lipidomic profiling of human breast cancer cells with different metastatic potentials. Oncotarget 2016; 7: 67111–67128.

Kononczuk J, Surazynski A, Czyzewska U, Prokop I, Tomczyk M, Palka J et al. αIIbβ3-integrin Ligands: Abciximab and Eptifibatide as Proapoptotic Factors in MCF-7 Human Breast Cancer Cells. Curr Drug Targets 2015; 16: 1429–1437.

Kulkarni YM, Yakisich JS, Azad N, Venkatadri R, Kaushik V, O’Doherty G et al. Anti-tumorigenic effects of a novel digitoxin derivative on both estrogen receptor-positive and triple-negative breast cancer cells. Tumour Biol 2017; 39: 1010428317705331.

Lampa M, Arlt H, He T, Ospina B, Reeves J, Zhang B et al. Glutaminase is essential for the growth of triple-negative breast cancer cells with a deregulated glutamine metabolism pathway and its suppression synergizes with mTOR inhibition. PLoS One 2017; 12: e0185092.

Lee H, Kang S, Kim W. Drug Repositioning for Cancer Therapy Based on Large-Scale Drug-Induced Transcriptional Signatures. PLoS One 2016; 11. doi:10.1371/journal.pone.0150460.

Li Z-Y, Yin Y-F, Guo Y, Li H, Xu M-Q, Liu M et al. Enhancing Anti-Tumor Activity of Sorafenib Mesoporous Silica Nanomatrix in Metastatic Breast Tumor and Hepatocellular Carcinoma via the Co-Administration with Flufenamic Acid. Int J Nanomedicine 2020; 15: 1809–1821.

Ma Z, Vosseller K. Cancer metabolism and elevated O-GlcNAc in oncogenic signaling. J Biol Chem 2014; 289: 34457–34465.

Machado KL, Marinello PC, Silva TNX, Silva CFN, Luiz RC, Cecchini R et al. Oxidative Stress in Caffeine Action on the Proliferation and Death of Human Breast Cancer Cells MCF-7 and MDA-MB-231. Nutr Cancer 2020; : 1–11.

Makwana V, Ryan P, Patel B, Dukie S-A, Rudrawar S. Essential role of O-GlcNAcylation in stabilization of oncogenic factors. Biochim Biophys Acta Gen Subj 2019; 1863: 1302–1317.

Martín M, Ruiz A, Muñoz M, Balil A, García-Mata J, Calvo L et al. Gemcitabine plus vinorelbine versus vinorelbine monotherapy in patients with metastatic breast cancer previously treated with anthracyclines and taxanes: final results of the phase III Spanish Breast Cancer Research Group (GEICAM) trial. Lancet Oncol 2007; 8: 219–225.

Menna PL, Parera RL, Cardama GA, Alonso DF, Gomez DE, Farina HG. Enhanced cytostatic activity of statins in mouse mammary carcinoma cells overexpressing β2-chimaerin. Mol Med Rep 2009; 2: 97–102.

Mrugala MM, Kim B, Sharma A, Johnson N, Graham C, Kurland BF et al. Phase II Study of Systemic High-dose Methotrexate and Intrathecal Liposomal Cytarabine for Treatment of Leptomeningeal Carcinomatosis From Breast Cancer. Clin Breast Cancer 2019; 19: 311–316.

Nie H, Yi W. O-GlcNAcylation, a sweet link to the pathology of diseases. J Zhejiang Univ Sci B 2019; 20: 437–448.

Niknafs B. Induction of apoptosis and non-apoptosis in human breast cancer cell line (MCF-7) by cisplatin and caffeine. Iran Biomed J 2011; 15: 130–133.

Opolski A, Mazurkiewicz M, Wietrzyk J, Kleinrok Z, Radzikowski C. The role of GABA-ergic system in human mammary gland pathology and in growth of transplantable murine mammary cancer. J Exp Clin Cancer Res 2000; 19: 383–390.

Parczyk K, Schneider MR. The future of antihormone therapy: innovations based on an established principle. J Cancer Res Clin Oncol 1996; 122: 383–396.

Park BJ, Whichard ZL, Corey SJ. Dasatinib synergizes with both cytotoxic and signal transduction inhibitors in heterogeneous breast cancer cell lines--lessons for design of combination targeted therapy. Cancer Lett 2012; 320: 104–110.

Parshad R, Hazrah P, Kumar S, Gupta SD, Ray R, Bal S. Effect of preoperative short course famotidine on TILs and survival in breast cancer. Indian J Cancer 2005; 42: 185–190.

Parshad R, Kapoor S, Gupta SD, Kumar A, Chattopadhyaya TK. Does famotidine enhance tumor infiltrating lymphocytes in breast cancer? Results of a randomized prospective pilot study. Acta Oncol 2002; 41: 362–365.

Pickup KE, Pardow F, Carbonell-Caballero J, Lioutas A, Villanueva-Cañas JL, Wright RHG et al. Expression of Oncogenic Drivers in 3D Cell Culture Depends on Nuclear ATP Synthesis by NUDT5. Cancers (Basel) 2019; 11. doi:10.3390/cancers11091337.

Rosendahl AH, Perks CM, Zeng L, Markkula A, Simonsson M, Rose C et al. Caffeine and Caffeic Acid Inhibit Growth and Modify Estrogen Receptor and Insulin-like Growth Factor I Receptor Levels in Human Breast Cancer. Clin Cancer Res 2015; 21: 1877–1887.

Rossi M, Rotblat B, Ansell K, Amelio I, Caraglia M, Misso G et al. High throughput screening for inhibitors of the HECT ubiquitin E3 ligase ITCH identifies antidepressant drugs as regulators of autophagy. Cell Death Dis 2014; 5: e1203.

Scher KS, Somlo G. Dasatinib : a novel therapy for breast cancer? Expert Opin Investig Drugs 2013; 22: 795–801.

Schug ZT, Peck B, Jones DT, Zhang Q, Grosskurth S, Alam IS et al. Acetyl-CoA synthetase 2 promotes acetate utilization and maintains cancer cell growth under metabolic stress. Cancer Cell 2015; 27: 57–71.

Shkurnikov MY, Nechaev IN, Khaustova NA, Krainova NA, Savelov NA, Grinevich VN et al. Expression profile of inflammatory breast cancer. Bull Exp Biol Med 2013; 155: 667–672.

Simigdala N, Gao Q, Pancholi S, Roberg-Larsen H, Zvelebil M, Ribas R et al. Cholesterol biosynthesis pathway as a novel mechanism of resistance to estrogen deprivation in estrogen receptor-positive breast cancer. Breast Cancer Res 2016; 18: 58.

Singh A, Nunes JJ, Ateeq B. Role and therapeutic potential of G-protein coupled receptors in breast cancer progression and metastases. Eur J Pharmacol 2015; 763: 178–183.

Stewart A, Maity B, Fisher RA. Two for the Price of One: G Protein-Dependent and -Independent Functions of RGS6 In Vivo. Prog Mol Biol Transl Sci 2015; 133: 123–151.

Strekalova E, Malin D, Weisenhorn EMM, Russell JD, Hoelper D, Jain A et al. S-adenosylmethionine biosynthesis is a targetable metabolic vulnerability of cancer stem cells. Breast Cancer Res Treat 2019; 175: 39–50.

Sun Y, Lin X, Chang H. Proliferation inhibition and apoptosis of breast cancer MCF-7 cells under the influence of colchicine. J BUON 2016; 21: 570–575.

Surguchov A. Intracellular Dynamics of Synucleins: ‘Here, There and Everywhere’. Int Rev Cell Mol Biol 2015; 320: 103–169.

Tadjuidje E, Wang TS, Pandey RN, Sumanas S, Lang RA, Hegde RS. The EYA tyrosine phosphatase activity is pro-angiogenic and is inhibited by benzbromarone. PLoS One 2012; 7: e34806.

Takemoto Y, Ito A, Niwa H, Okamura M, Fujiwara T, Hirano T et al. Identification of Cyproheptadine as an Inhibitor of SET Domain Containing Lysine Methyltransferase 7/9 (Set7/9) That Regulates Estrogen-Dependent Transcription. J Med Chem 2016; 59: 3650–3660.

Takeuchi H, Saoo K, Matsuda Y, Yokohira M, Yamakawa K, Zeng Y et al. Dose dependent inhibitory effects of dietary 8-methoxypsoralen on NNK-induced lung tumorigenesis in female A/J mice. Cancer Lett 2006; 234: 232–238.

Taylor JM, Simpson RU. Inhibition of cancer cell growth by calcium channel antagonists in the athymic mouse. Cancer Res 1992; 52: 2413–2418.

Urbaniak A, Jousheghany F, Piña-Oviedo S, Yuan Y, Majcher-Uchańska U, Klejborowska G et al. Carbamate derivatives of colchicine show potent activity towards primary acute lymphoblastic leukemia and primary breast cancer cells-in vitro and ex vivo study. J Biochem Mol Toxicol 2020; 34: e22487.

Vaughan RA, Gannon NP, Garcia-Smith R, Licon-Munoz Y, Barberena MA, Bisoffi M et al. β-alanine suppresses malignant breast epithelial cell aggressiveness through alterations in metabolism and cellular acidity in vitro. Mol Cancer 2014; 13: 14.

Vazquez-Martin A, Ropero S, Brunet J, Colomer R, Menendez JA. Inhibition of Fatty Acid Synthase (FASN) synergistically enhances the efficacy of 5-fluorouracil in breast carcinoma cells. Oncol Rep 2007; 18: 973–980.

Very N, Vercoutter-Edouart A-S, Lefebvre T, Hardivillé S, El Yazidi-Belkoura I. Cross-Dysregulation of O-GlcNAcylation and PI3K/AKT/mTOR Axis in Human Chronic Diseases. Front Endocrinol (Lausanne) 2018; 9. doi:10.3389/fendo.2018.00602.

Wang RC, Chen X, Parissenti AM, Joy AA, Tuszynski J, Brindley DN et al. Sensitivity of docetaxel-resistant MCF-7 breast cancer cells to microtubule-destabilizing agents including vinca alkaloids and colchicine-site binding agents. PLoS One 2017; 12: e0182400.

Wang X, Wan J, Xu Z, Jiang S, Ji L, Liu Y et al. Identification of competitive endogenous RNAs network in breast cancer. Cancer Med 2019; 8: 2392–2403.

Wu X, Li X, Fu Q, Cao Q, Chen X, Wang M et al. AKR1B1 promotes basal-like breast cancer progression by a positive feedback loop that activates the EMT program. J Exp Med 2017; 214: 1065–1079.

Yu L, Li K, Xu Z, Cui G, Zhang X. Integrated omics and gene expression analysis identifies the loss of metabolite-metabolite correlations in small cell lung cancer. Onco Targets Ther 2018; 11: 3919–3929.

Zhang Y, Li R-J, Ying X, Tian W, Yao H-J, Men Y et al. Targeting therapy with mitosomal daunorubicin plus amlodipine has the potential to circumvent intrinsic resistant breast cancer. Mol Pharm 2011; 8: 162–175.
