# Supplementary material for: Path to Clonal Theranostics in Luminal Breast Cancers
Source: Front Oncol. 2022 Jan 13;11:802177. doi: 10.3389/fonc.2021.802177 (PMC8793283; doi:10.3389/fonc.2021.802177)

Supplementary material 4: MALDI MSI of 52 cases of primary tumors showing the spatial proteomic heterogeneity of the tumors. In each sample vignette, the MALDI MS imaging is displayed with the histological HPS picture (upper left), the principal component analysis of the proteomic clones (upper right), the segmentation tree (middle right), and the spectra of the clones (bottom right). S: stroma area and T: tumor area selected for microproteomics

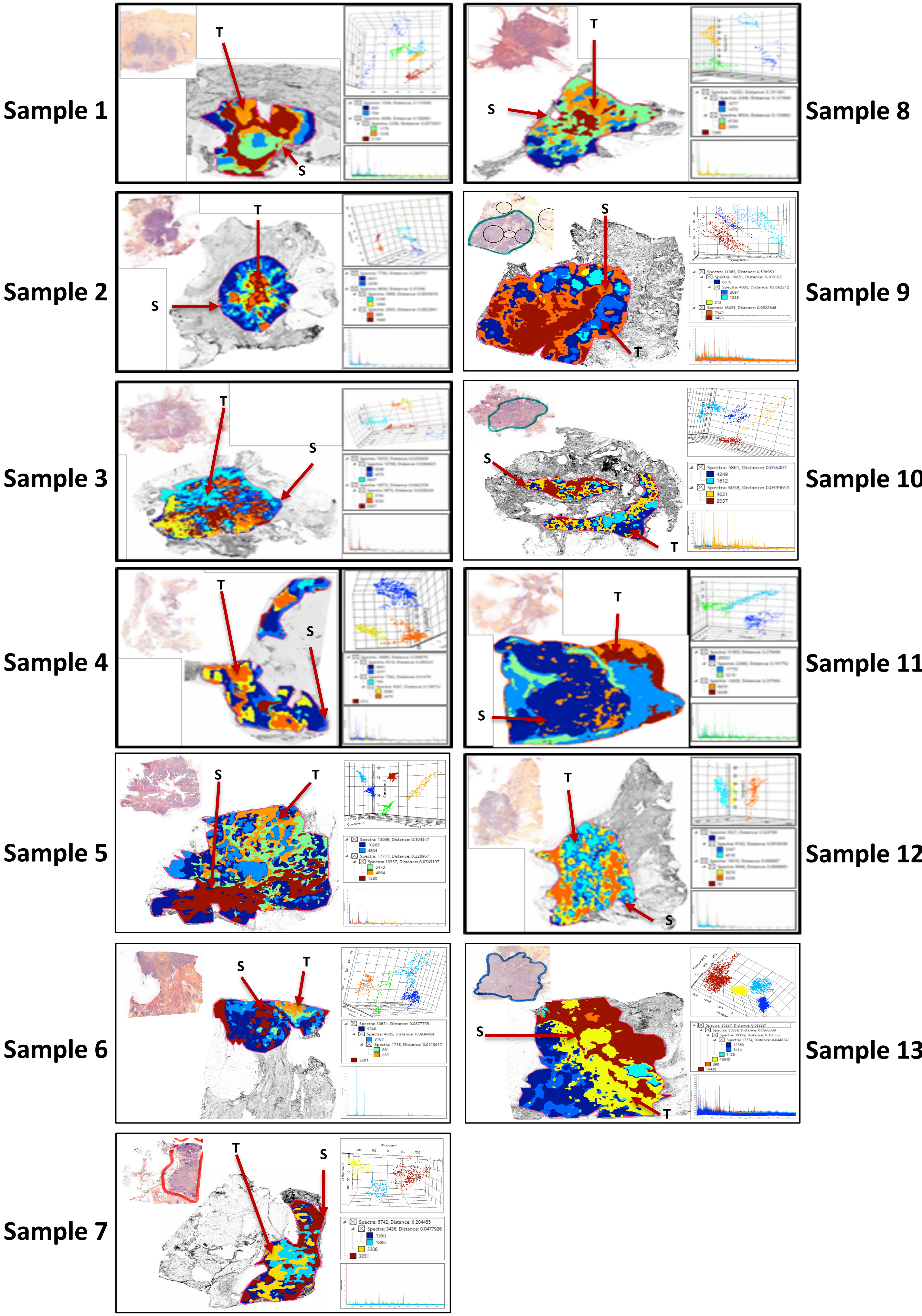
Sample 1
Sample 2
Sample 3
Sample 4
Sample 5
Sample 6
Sample 7
Sample 8
Sample 9
Sample 10
Sample 11
Sample 12
Sample 13

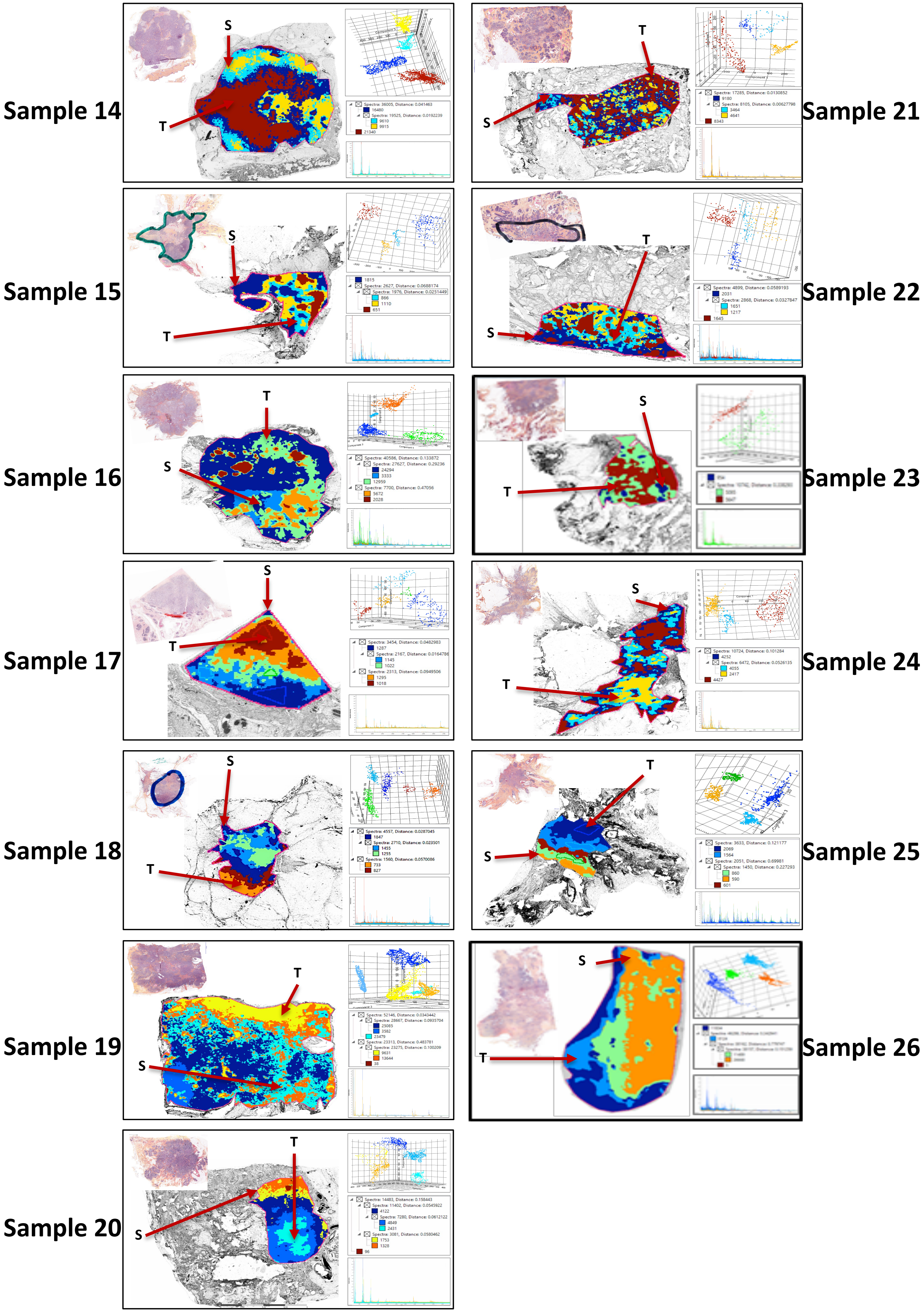
Sample 14
Sample 21
Sample 15
Sample 22
Sample 16
Sample 23
Sample 17
Sample 24
Sample 18
Sample 25
Sample 19
Sample 26
Sample 20

Sample 27

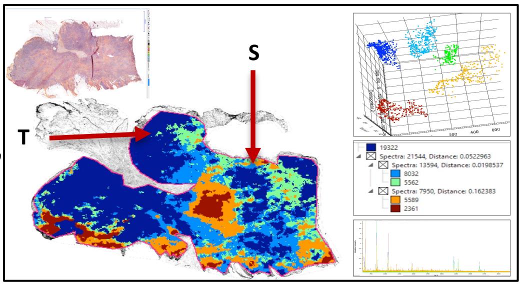

Sample 34

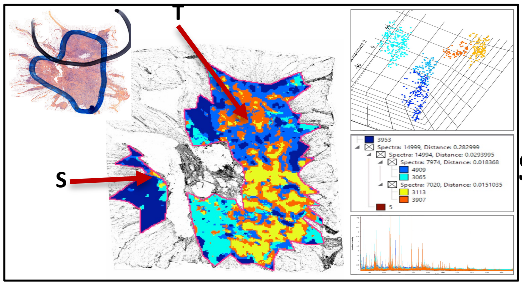

Sample 28

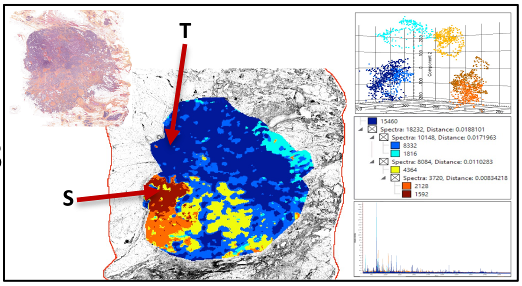

Sample 35

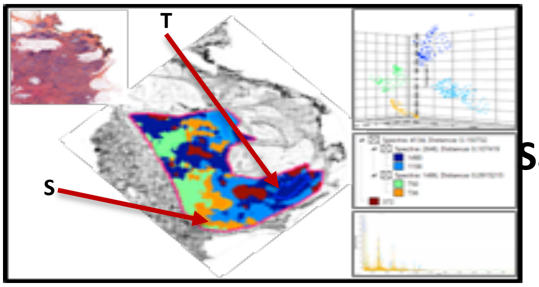

Sample 29

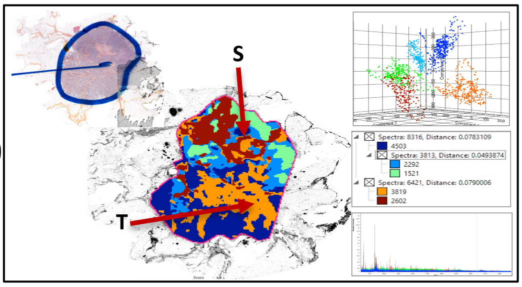

Sample 36

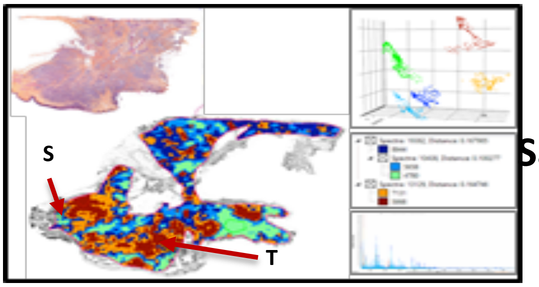

Sample 30

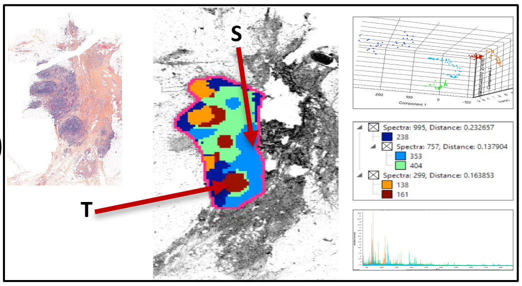

Sample 37

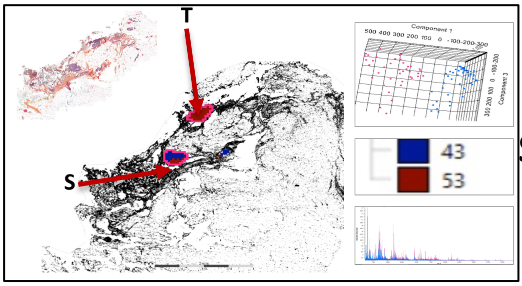

Sample 31

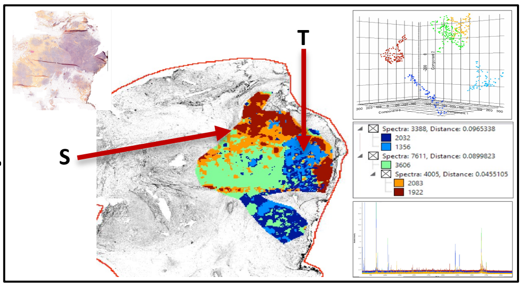

Sample 38

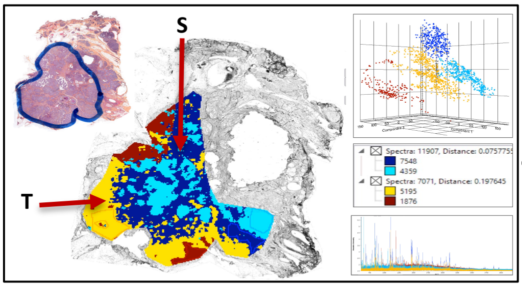

Sample 32

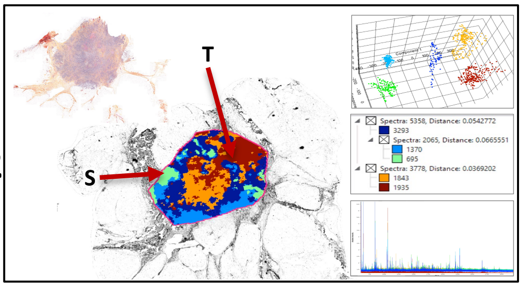

Sample 39

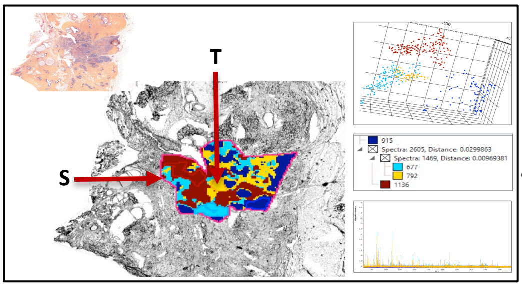

Sample 33

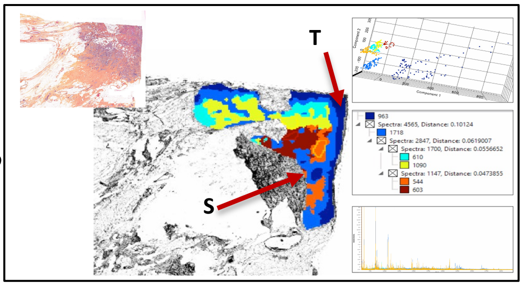

Sample 40

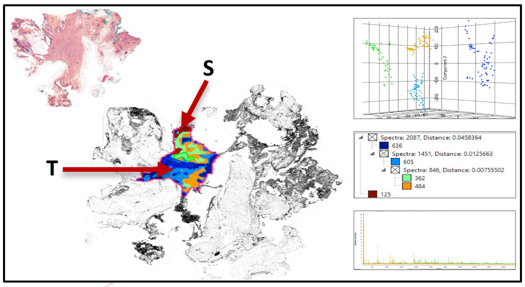

Sample 47

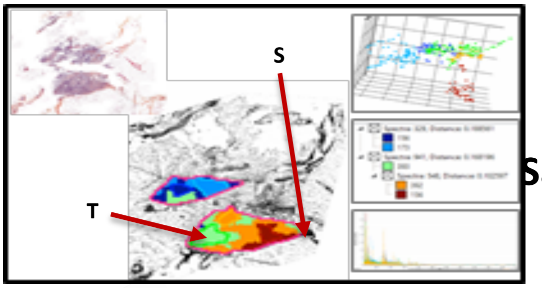

Sample 41

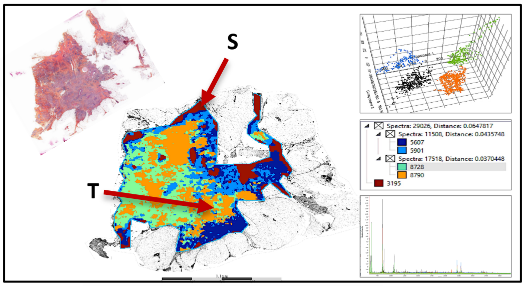

Sample 48

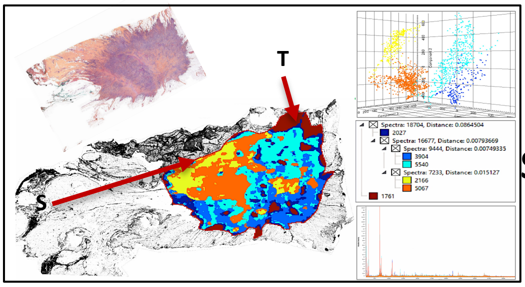

Sample 42

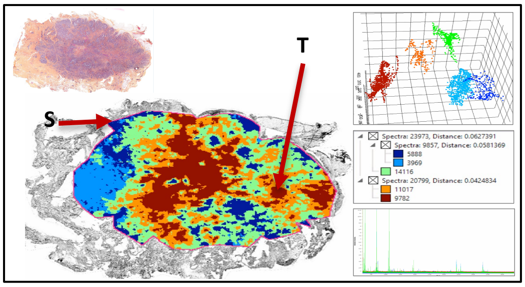

Sample 49

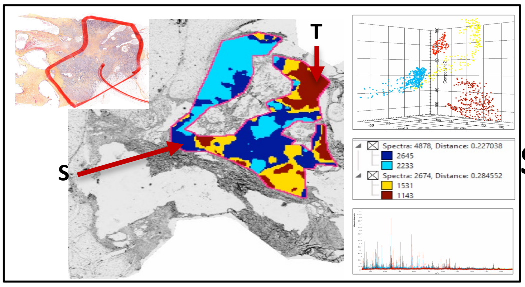

Sample 43

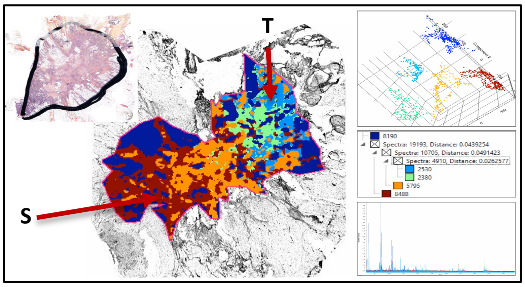

Sample 50

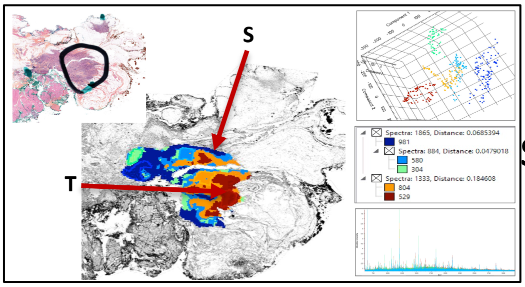

Sample 44

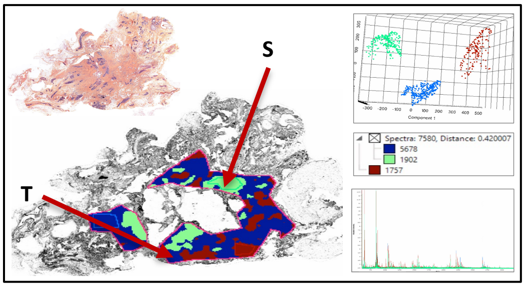

Sample 51

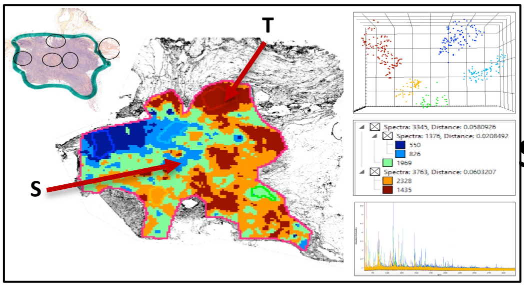

Sample 45

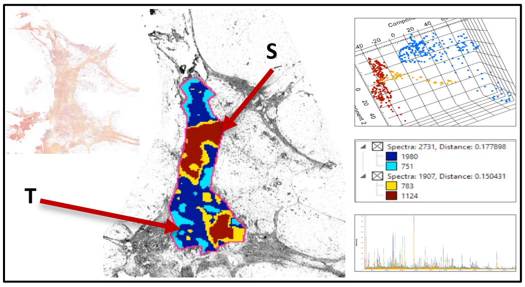

Sample 52

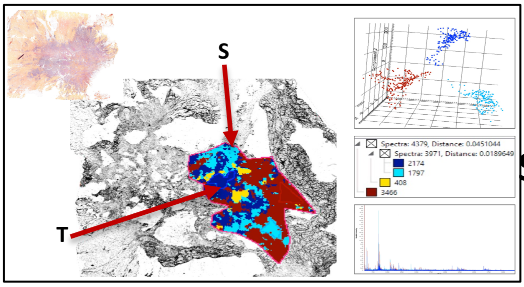

Sample 46

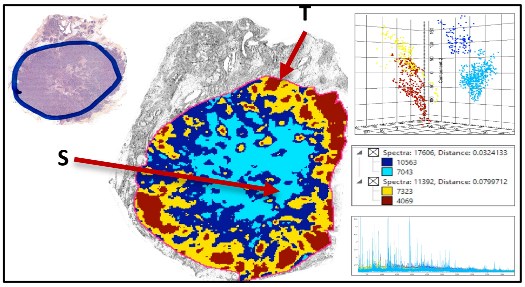

Supplement: Supplementary Material 1 — TCGA database of mutations and CNV alterations in early and advanced breast cancers. [file DataSheet_1.zip › Data Sheet 4.pdf]
