# Supplementary material for: Path to Clonal Theranostics in Luminal Breast Cancers
Source: Front Oncol. 2022 Jan 13;11:802177. doi: 10.3389/fonc.2021.802177 (PMC8793283; doi:10.3389/fonc.2021.802177)

Supplementary material 5: MALDI MSI of 24 cases of metastases showing the spatial proteomic heterogeneity of the tumors. In each sample vignette, the MALDI MS imaging is displayed with the histological HPS picture (upper left), the principal component analysis of the proteomic clones (upper right), the segmentation tree (middle right), and the spectra of the clones (bottom right). T: tumor area selected for microproteomics

Sample 53

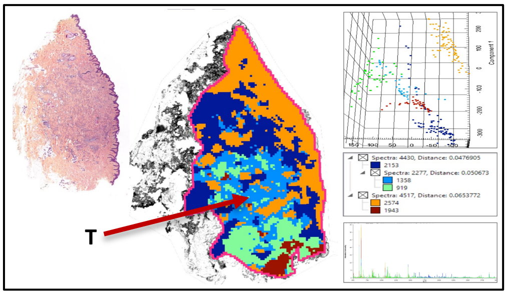

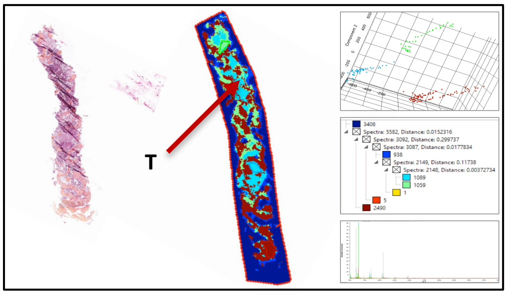

Sample 59

Sample 54

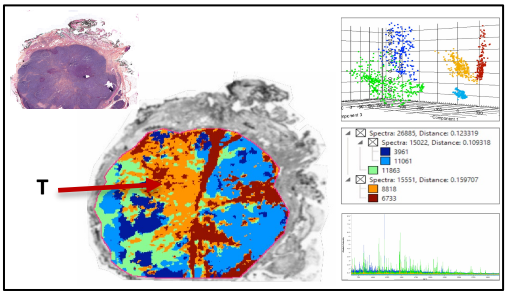

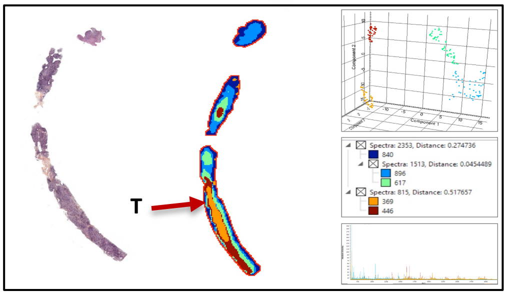

Sample 60

Sample 55

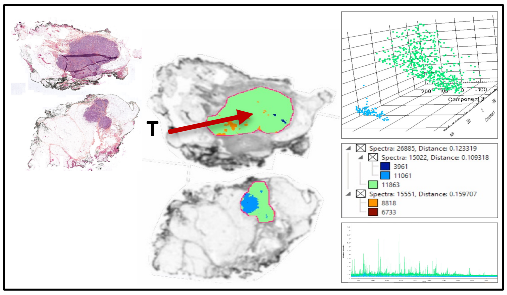

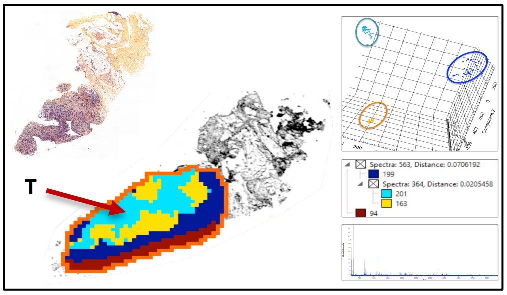

Sample 61

Sample 56

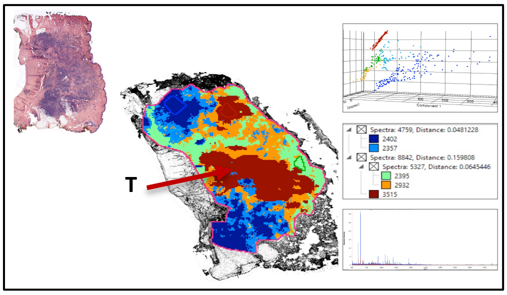

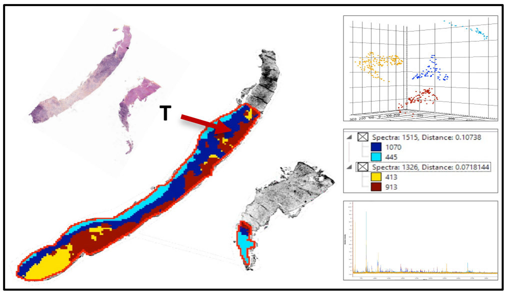

Sample 62

Sample 57

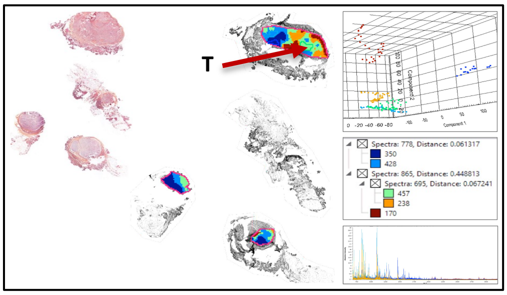

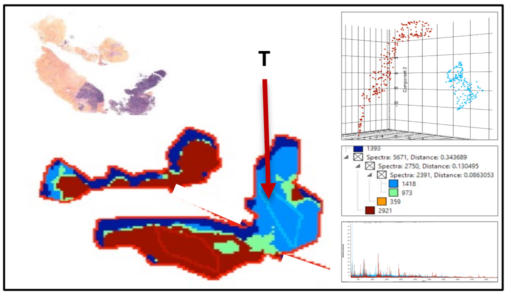

Sample 63

Sample 58

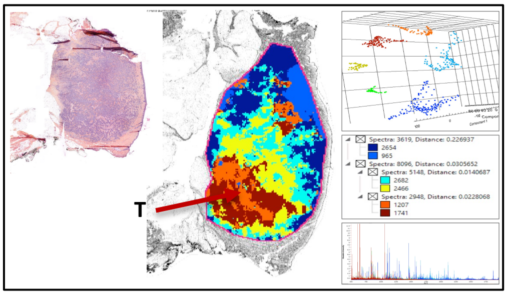

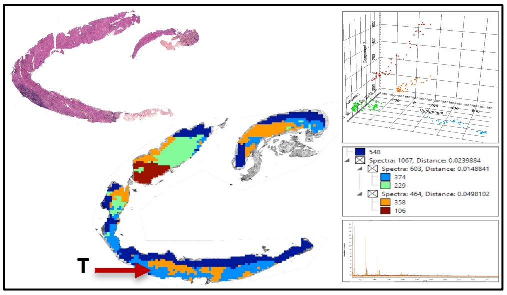

Sample 64

Sample 65

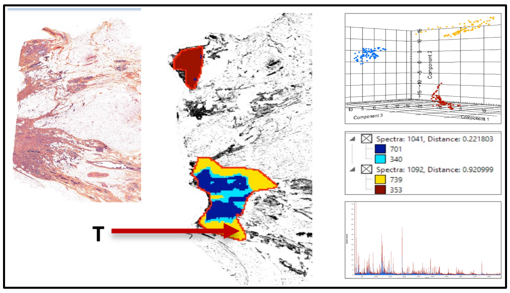

Sample 66

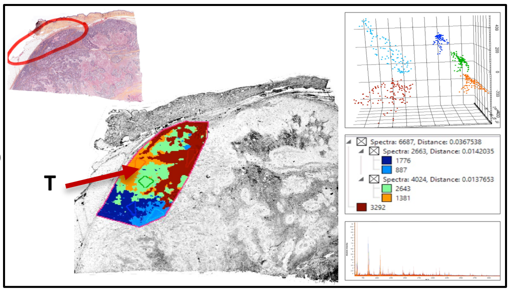

Sample 67

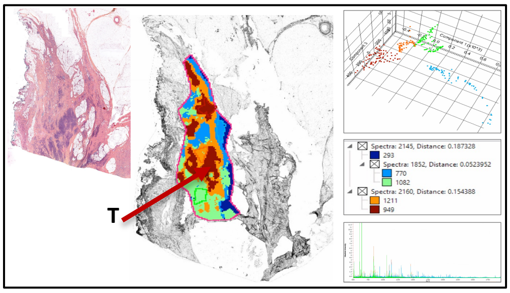

Sample 68

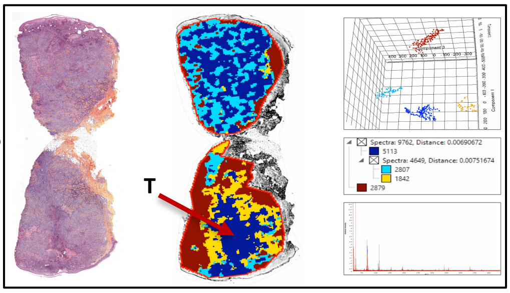

Sample 69

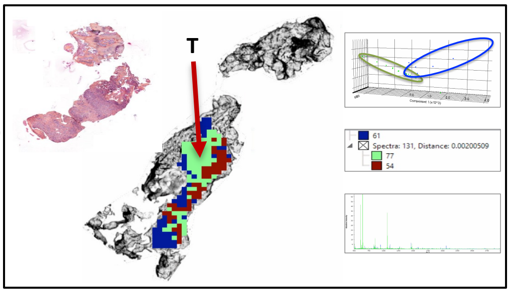

Sample 70

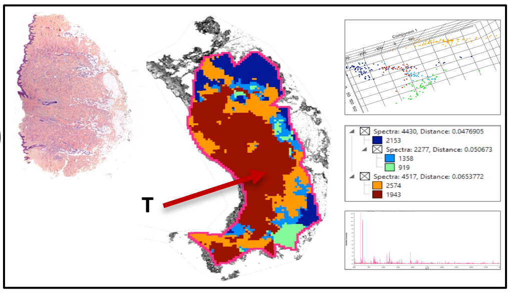

Sample 71

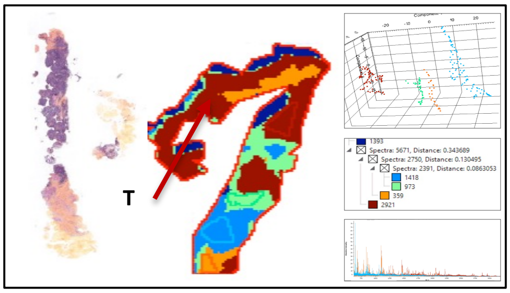

Sample 72

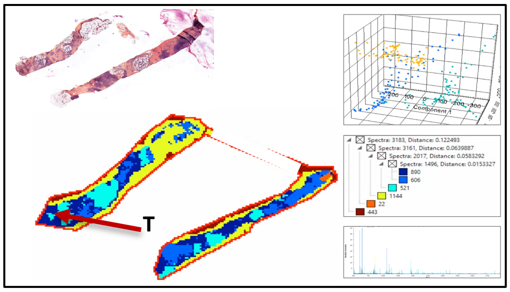

Sample 73

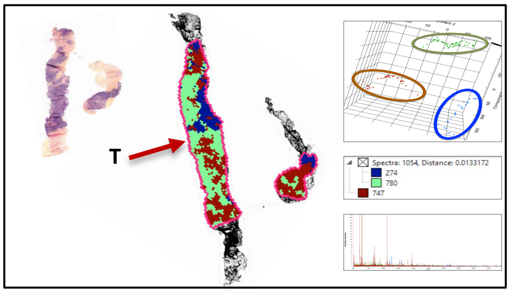

Sample 74

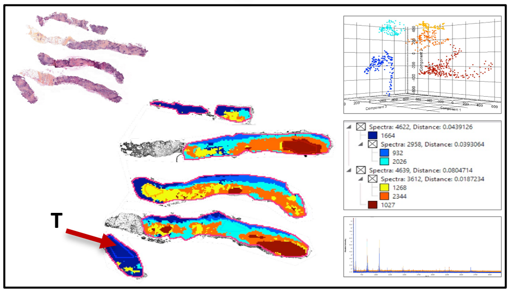

Sample 75

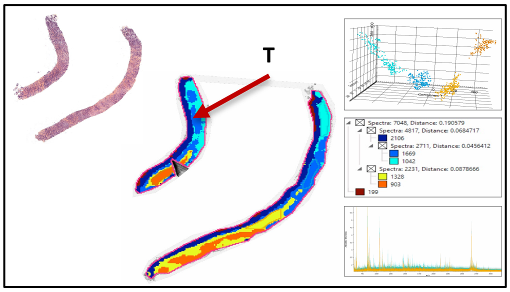

Sample 76

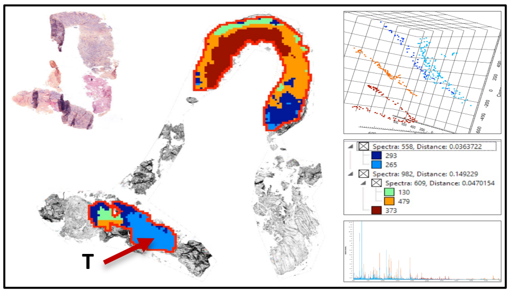

Supplement: Supplementary Material 1 — TCGA database of mutations and CNV alterations in early and advanced breast cancers. [file DataSheet_1.zip › Data Sheet 5.pdf]
