# Supplementary material for: Path to Clonal Theranostics in Luminal Breast Cancers
Source: Front Oncol. 2022 Jan 13;11:802177. doi: 10.3389/fonc.2021.802177 (PMC8793283; doi:10.3389/fonc.2021.802177)
Supplement: Supplementary Material 1 — TCGA database of mutations and CNV alterations in early and advanced breast cancers. [file DataSheet_1.zip › Data Sheet 7.pdf]

Supplementary material 7: Panther analysis of the clonal proteome landscape showing (A) the protein class distribution (in %), and (B) a comparison of the distribution between primary tumors and stroma (relative difference in % in blue) and between primary tumors and metastases (relative difference in % in red).

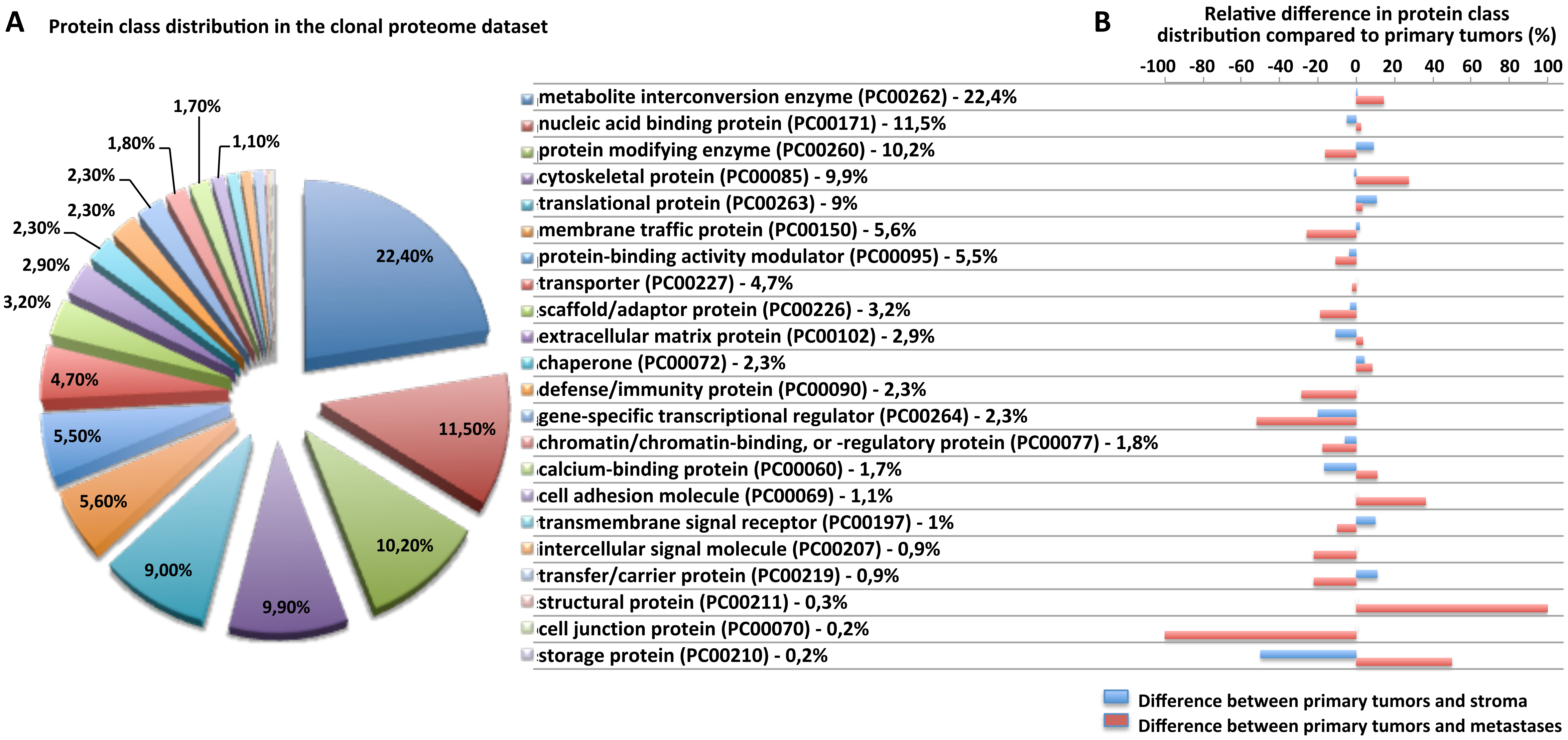
A
Protein class distribution in the clonal proteome dataset
22,40%
11,50%
10,20%
9,90%
9,00%
5,60%
5,50%
4,70%
3,20%
2,90%
2,30%
2,30%
2,30%
1,80%
1,70%
1,10%
B
Relative difference in protein class distribution compared to primary tumors (%)
-100 -80 -60 -40 -20 0 20 40 60 80 100
metabolite interconversion enzyme (PC00262) - 22,4%
nucleic acid binding protein (PC00171) - 11,5%
protein modifying enzyme (PC00260) - 10,2%
cytoskeletal protein (PC00085) - 9,9%
translational protein (PC00263) - 9%
membrane traffic protein (PC00150) - 5,6%
protein-binding activity modulator (PC00095) - 5,5%
transporter (PC00227) - 4,7%
scaffold/adaptor protein (PC00226) - 3,2%
extracellular matrix protein (PC00102) - 2,9%
chaperone (PC00072) - 2,3%
defense/immunity protein (PC00090) - 2,3%
gene-specific transcriptional regulator (PC00264) - 2,3%
chromatin/chromatin-binding, or -regulatory protein (PC00077) - 1,8%
calcium-binding protein (PC00060) - 1,7%
cell adhesion molecule (PC00069) - 1,1%
transmembrane signal receptor (PC00197) - 1%
intercellular signal molecule (PC00207) - 0,9%
transfer/carrier protein (PC00219) - 0,9%
structural protein (PC00211) - 0,3%
cell junction protein (PC00070) - 0,2%
storage protein (PC00210) - 0,2%
Difference between primary tumors and stroma
Difference between primary tumors and metastases
