# Supplementary material for: Path to Clonal Theranostics in Luminal Breast Cancers
Source: Front Oncol. 2022 Jan 13;11:802177. doi: 10.3389/fonc.2021.802177 (PMC8793283; doi:10.3389/fonc.2021.802177)
Supplement: Supplementary Material 1 — TCGA database of mutations and CNV alterations in early and advanced breast cancers. [file DataSheet_1.zip › Data Sheet 1.doc]

**Supplementary material 1:** TCGA database of mutations and CNV alterations in early and advanced breast cancers

| **Genes TCGA early BC** | **Name** | **Genes TCGA advanced BC** | **Name** |
| --- | --- | --- | --- |
| **TP53** | tumor protein p53 | **PIK3CA** | phosphatidylinositol-4,5-bisphosphate 3-kinase, catalytic subunit alpha |
| **PIK3CA** | phosphatidylinositol-4,5-bisphosphate 3-kinase, catalytic subunit alpha | **TP53** | tumor protein p53 |
| **CDH1** | cadherin 1, type 1, E-cadherin (epithelial) | **CLIP1** | CAP-GLY domain containing linker protein 1 |
| **GATA3** | GATA binding protein 3 | **MUC1** | mucin 1, cell surface associated |
| **KMT2C** | lysine (K)-specific methyltransferase 2C | **TSHR** | thyroid stimulating hormone receptor |
| **MAP3K1** | mitogen-activated protein kinase kinase kinase 1, E3 ubiquitin protein ligase | **FGFR2** | fibroblast growth factor receptor 2 |
| **PTEN** | phosphatase and tensin homolog | **CBLB** | Cbl proto-oncogene B, E3 ubiquitin protein ligase |
| **NCOR1** | nuclear receptor corepressor 1 | **IL21R** | interleukin 21 receptor |
| **NF1** | neurofibromin 1 | **AMER1** | APC membrane recruitment protein 1 |
| **RUNX1** | runt-related transcription factor 1 | **NSD1** | nuclear receptor binding SET domain protein 1 |
| **ARID1A** | AT rich interactive domain 1A (SWI-like) | **ETNK1** | ethanolamine kinase 1 |
| **PIK3R1** | phosphoinositide-3-kinase, regulatory subunit 1 (alpha) | **AKAP9** | A kinase (PRKA) anchor protein 9 |
| **MAP2K4** | mitogen-activated protein kinase kinase 4 | **PTPRB** | protein tyrosine phosphatase, receptor type, B |
| **RNF213** | ring finger protein 213 | **ERBB2** | erb-b2 receptor tyrosine kinase 2 |
| **KMT2D** | lysine (K)-specific methyltransferase 2D | **NOTCH1** | notch 1 |
| **SPEN** | spen family transcriptional repressor | **TPR** | translocated promoter region, nuclear basket protein |
| **AKAP9** | A kinase (PRKA) anchor protein 9 | **FLI1** | Fli-1 proto-oncogene, ETS transcription factor |
| **FAT1** | FAT atypical cadherin 1 | **CDH1** | cadherin 1, type 1, E-cadherin (epithelial) |
| **MYH9** | myosin, heavy chain 9, non-muscle | **MEN1** | multiple endocrine neoplasia I |
| **TBX3** | T-box 3 | **MLLT10** | myeloid/lymphoid or mixed-lineage leukemia (trithorax homolog, Drosophila); translocated to, 10 |
| **MED12** | mediator complex subunit 12 | **CAMTA1** | calmodulin binding transcription activator 1 |
| **KMT2A** | lysine (K)-specific methyltransferase 2A | **AKT1** | v-akt murine thymoma viral oncogene homolog 1 |
| **MLLT4** | myeloid/lymphoid or mixed-lineage leukemia (trithorax homolog, Drosophila); translocated to, 4 | **NFATC2** | nuclear factor of activated T-cells, cytoplasmic, calcineurin-dependent 2 |
| **ATRX** | alpha thalassemia/mental retardation syndrome X-linked | **NOTCH2** | notch 2 |
| **FOXA1** | forkhead box A1 | **CHD4** | chromodomain helicase DNA binding protein 4 |
| **PTPRB** | protein tyrosine phosphatase, receptor type, B | **FLCN** | folliculin |
| **ERBB2** | erb-b2 receptor tyrosine kinase 2 | **MSI2** | musashi RNA-binding protein 2 |
| **ATM** | ATM serine/threonine kinase | **CREB1** | cAMP responsive element binding protein 1 |
| **TPR** | translocated promoter region, nuclear basket protein | **RALGDS** | ral guanine nucleotide dissociation stimulator |
| **AKT1** | v-akt murine thymoma viral oncogene homolog 1 | **ABL2** | ABL proto-oncogene 2, non-receptor tyrosine kinase |
| **UBR5** | ubiquitin protein ligase E3 component n-recognin 5 | **NDRG1** | N-myc downstream regulated 1 |
| **CHD4** | chromodomain helicase DNA binding protein 4 | **KCNJ5** | potassium channel, inwardly rectifying subfamily J, member 5 |
| **BRCA1** | breast cancer 1, early onset | **ERBB3** | erb-b2 receptor tyrosine kinase 3 |
| **ARID1B** | AT rich interactive domain 1B (SWI1-like) | **CHEK2** | checkpoint kinase 2 |
| **MTOR** | mechanistic target of rapamycin (serine/threonine kinase) | **MYCL** | v-myc avian myelocytomatosis viral oncogene lung carcinoma derived homolog |
| **BRCA2** | breast cancer 2, early onset | **BCL11B** | B-cell CLL/lymphoma 11B (zinc finger protein) |
| **NOTCH2** | notch 2 | **PTPN11** | protein tyrosine phosphatase, non-receptor type 11 |
| **SETD2** | SET domain containing 2 | **PCSK7** | proprotein convertase subtilisin/kexin type 7 |
| **CBFB** | core-binding factor, beta subunit | **GNAS** | GNAS complex locus |
| **RB1** | retinoblastoma 1 | **SOCS1** | suppressor of cytokine signaling 1 |
| **GRIN2A** | glutamate receptor, ionotropic, N-methyl D-aspartate 2A | **GNA11** | guanine nucleotide binding protein (G protein), alpha 11 (Gq class) |
| **FAT4** | FAT atypical cadherin 4 | **RAD21** | RAD21 homolog (S. pombe) |
| **RUNX1T1** | runt-related transcription factor 1; translocated to, 1 (cyclin D-related) | **COX6C** | cytochrome c oxidase subunit VIc |
| **ERBB3** | erb-b2 receptor tyrosine kinase 3 | **LIFR** | leukemia inhibitory factor receptor alpha |
| **TET1** | tet methylcytosine dioxygenase 1 | **PMS2** | PMS2 postmeiotic segregation increased 2 (S. cerevisiae) |
| **ATR** | ATR serine/threonine kinase | **ACKR3** | atypical chemokine receptor 3 |
| **SF3B1** | splicing factor 3b, subunit 1, 155kDa | **MAP2K2** | mitogen-activated protein kinase kinase 2 |
| **PDE4DIP** | phosphodiesterase 4D interacting protein | **EZR** | ezrin |
| **AMER1** | APC membrane recruitment protein 1 | **FGFR1OP** | FGFR1 oncogene partner |
| **KDM6A** | lysine (K)-specific demethylase 6A | **CARS** | cysteinyl-tRNA synthetase |
| **CREBBP** | CREB binding protein | **MTOR** | mechanistic target of rapamycin (serine/threonine kinase) |
| **TRRAP** | transformation/transcription domain-associated protein | **PPP6C** | protein phosphatase 6, catalytic subunit |
| **STAG2** | stromal antigen 2 | **FANCF** | Fanconi anemia, complementation group F |
| **MYH11** | myosin, heavy chain 11, smooth muscle | **ATM** | ATM serine/threonine kinase |
| **ZFHX3** | zinc finger homeobox 3 | **TET2** | tet methylcytosine dioxygenase 2 |
| **KAT6B** | K(lysine) acetyltransferase 6B | **FH** | fumarate hydratase |
| **CTCF** | CCCTC-binding factor (zinc finger protein) | **PRF1** | perforin 1 (pore forming protein) |
| **CASP8** | caspase 8, apoptosis-related cysteine peptidase | **IKZF1** | IKAROS family zinc finger 1 (Ikaros) |
| **ATP2B3** | ATPase, Ca++ transporting, plasma membrane 3 | **FOXA1** | forkhead box A1 |
| **EP300** | E1A binding protein p300 | **NUP214** | nucleoporin 214kDa |
| **TET2** | tet methylcytosine dioxygenase 2 | **BRAF** | B-Raf proto-oncogene, serine/threonine kinase |
| **ROS1** | ROS proto-oncogene 1 , receptor tyrosine kinase | **SMAD3** | SMAD family member 3 |
| **SMARCA4** | SWI/SNF related, matrix associated, actin dependent regulator of chromatin, subfamily a, member 4 | **USP8** | ubiquitin specific peptidase 8 |
| **EGFR** | epidermal growth factor receptor | **VTI1A** | vesicle transport through interaction with t-SNAREs 1A |
| **AFF3** | AF4/FMR2 family, member 3 | **SF3B1** | splicing factor 3b, subunit 1, 155kDa |
| **ARID2** | AT rich interactive domain 2 (ARID, RFX-like) | **RBM15** | RNA binding motif protein 15 |
| **ERBB4** | erb-b2 receptor tyrosine kinase 4 | **FLT3** | fms-related tyrosine kinase 3 |
| **NIN** | ninein (GSK3B interacting protein) | **NKX2-1** | NK2 homeobox 1 |
| **RANBP2** | RAN binding protein 2 | **TAL1** | T-cell acute lymphocytic leukemia 1 |
| **CNTRL** | centriolin | **OLIG2** | oligodendrocyte lineage transcription factor 2 |
| **FBXW7** | F-box and WD repeat domain containing 7, E3 ubiquitin protein ligase | **sept-05** | septin 5 |
| **FANCD2** | Fanconi anemia, complementation group D2 | **CDX2** | caudal type homeobox 2 |
| **AFF1** | AF4/FMR2 family, member 1 | **HIF1A** | hypoxia inducible factor 1, alpha subunit (basic helix-loop-helix transcription factor) |
| **CACNA1D** | calcium channel, voltage-dependent, L type, alpha 1D subunit | **MYOD1** | myogenic differentiation 1 |
| **KDM5A** | lysine (K)-specific demethylase 5A | **LZTR1** | leucine-zipper-like transcription regulator 1 |
| **BCR** | breakpoint cluster region | **MDM2** | MDM2 proto-oncogene, E3 ubiquitin protein ligase |
| **BLM** | Bloom syndrome, RecQ helicase-like | **FAS** | Fas cell surface death receptor |
| **KAT6A** | K(lysine) acetyltransferase 6A | **HOXC13** | homeobox C13 |
| **PTCH1** | patched 1 | **ELL** | elongation factor RNA polymerase II |
| **BCL9** | B-cell CLL/lymphoma 9 | **MAX** | MYC associated factor X |
| **APC** | adenomatous polyposis coli | **PSIP1** | PC4 and SFRS1 interacting protein 1 |
| **PTPRC** | protein tyrosine phosphatase, receptor type, C | **FOXL2** | forkhead box L2 |
| **TRIP11** | thyroid hormone receptor interactor 11 | **FBXW7** | F-box and WD repeat domain containing 7, E3 ubiquitin protein ligase |
| **ASXL1** | additional sex combs like transcriptional regulator 1 | **HNF1A** | HNF1 homeobox A |
| **MYO5A** | myosin VA (heavy chain 12, myoxin) | **HSP90AB1** | heat shock protein 90kDa alpha (cytosolic), class B member 1 |
| **NSD1** | nuclear receptor binding SET domain protein 1 | **PTPRC** | protein tyrosine phosphatase, receptor type, C |
| **NUP98** | nucleoporin 98kDa | **DCTN1** | dynactin 1 |
| **NUMA1** | nuclear mitotic apparatus protein 1 | **SRSF2** | serine/arginine-rich splicing factor 2 |
| **MECOM** | MDS1 and EVI1 complex locus | **NUMA1** | nuclear mitotic apparatus protein 1 |
| **CLTC** | clathrin, heavy chain (Hc) | **PML** | promyelocytic leukemia |
| **MAP3K13** | mitogen-activated protein kinase kinase kinase 13 | **MYH9** | myosin, heavy chain 9, non-muscle |
| **XPO1** | exportin 1 | **MECOM** | MDS1 and EVI1 complex locus |
| **ESR1** | estrogen receptor 1 | **ECT2L** | epithelial cell transforming 2 like |
| **CDK12** | cyclin-dependent kinase 12 | **BRCA1** | breast cancer 1, early onset |
| **BRIP1** | BRCA1 interacting protein C-terminal helicase 1 | **FUS** | FUS RNA binding protein |
| **WHSC1L1** | Wolf-Hirschhorn syndrome candidate 1-like 1 | **PAFAH1B2** | platelet-activating factor acetylhydrolase 1b, catalytic subunit 2 (30kDa) |
| **JAK3** | Janus kinase 3 | **SDHD** | succinate dehydrogenase complex, subunit D, integral membrane protein |
| **EIF4A2** | eukaryotic translation initiation factor 4A2 | **PIM1** | Pim-1 proto-oncogene, serine/threonine kinase |
| **BCOR** | BCL6 corepressor | **RAC1** | ras-related C3 botulinum toxin substrate 1 (rho family, small GTP binding protein Rac1) |
| **CLIP1** | CAP-GLY domain containing linker protein 1 | **MYCN** | v-myc avian myelocytomatosis viral oncogene neuroblastoma derived homolog |
| **ZNF521** | zinc finger protein 521 | **CD274** | CD274 molecule |
| **ZMYM2** | zinc finger, MYM-type 2 | **ZBTB16** | zinc finger and BTB domain containing 16 |
| **KTN1** | kinectin 1 (kinesin receptor) | **EML4** | echinoderm microtubule associated protein like 4 |
| **DDX5** | DEAD (Asp-Glu-Ala-Asp) box helicase 5 | **TFRC** | transferrin receptor |
| **CARD11** | caspase recruitment domain family, member 11 | **FHIT** | fragile histidine triad |
| **POLE** | polymerase (DNA directed), epsilon, catalytic subunit | **PTEN** | phosphatase and tensin homolog |
| **PLCG1** | phospholipase C, gamma 1 | **FANCA** | Fanconi anemia, complementation group A |
| **CUX1** | cut-like homeobox 1 | **CBL** | Cbl proto-oncogene, E3 ubiquitin protein ligase |
| **COL1A1** | collagen, type I, alpha 1 | **MAFB** | v-maf avian musculoaponeurotic fibrosarcoma oncogene homolog B |
| **GNAS** | GNAS complex locus | **TNFRSF17** | tumor necrosis factor receptor superfamily, member 17 |
| **CDKN1B** | cyclin-dependent kinase inhibitor 1B (p27, Kip1) | **CLTC** | clathrin, heavy chain (Hc) |
| **FGFR2** | fibroblast growth factor receptor 2 | **JAK2** | Janus kinase 2 |
| **CBLB** | Cbl proto-oncogene B, E3 ubiquitin protein ligase | **ERCC5** | excision repair cross-complementation group 5 |
| **JAK1** | Janus kinase 1 | **TERT** | telomerase reverse transcriptase |
| **MAML2** | mastermind-like 2 (Drosophila) | **TMPRSS2** | transmembrane protease, serine 2 |
| **TSC2** | tuberous sclerosis 2 | **SUZ12** | SUZ12 polycomb repressive complex 2 subunit |
| **CAMTA1** | calmodulin binding transcription activator 1 | **IL7R** | interleukin 7 receptor |
| **PTPN13** | protein tyrosine phosphatase, non-receptor type 13 (APO-1/CD95 (Fas)-associated phosphatase) | **MAP3K13** | mitogen-activated protein kinase kinase kinase 13 |
| **ALK** | anaplastic lymphoma receptor tyrosine kinase | **FUBP1** | far upstream element (FUSE) binding protein 1 |
| **DICER1** | dicer 1, ribonuclease type III | **CBFA2T3** | core-binding factor, runt domain, alpha subunit 2; translocated to, 3 |
| **IKBKB** | inhibitor of kappa light polypeptide gene enhancer in B-cells, kinase beta | **ARID1A** | AT rich interactive domain 1A (SWI-like) |
| **PBRM1** | polybromo 1 | **ASPSCR1** | alveolar soft part sarcoma chromosome region, candidate 1 |
| **CYLD** | cylindromatosis (turban tumor syndrome) | **NF2** | neurofibromin 2 (merlin) |
| **DCTN1** | dynactin 1 | **CIITA** | class II, major histocompatibility complex, transactivator |
| **AFF4** | AF4/FMR2 family, member 4 | **CCDC6** | coiled-coil domain containing 6 |
| **FGFR1** | fibroblast growth factor receptor 1 | **TNFRSF14** | tumor necrosis factor receptor superfamily, member 14 |
| **PDGFRA** | platelet-derived growth factor receptor, alpha polypeptide | **KLF6** | Kruppel-like factor 6 |
| **TNFAIP3** | tumor necrosis factor, alpha-induced protein 3 | **EWSR1** | EWS RNA-binding protein 1 |
| **ATP1A1** | ATPase, Na+/K+ transporting, alpha 1 polypeptide | **FGFR3** | fibroblast growth factor receptor 3 |
| **SMAD4** | SMAD family member 4 | **LCP1** | lymphocyte cytosolic protein 1 (L-plastin) |
| **STAT6** | signal transducer and activator of transcription 6, interleukin-4 induced | **HOXA9** | homeobox A9 |
| **RANBP17** | RAN binding protein 17 | **DAXX** | death-domain associated protein |
| **LIFR** | leukemia inhibitory factor receptor alpha | **PRDM1** | PR domain containing 1, with ZNF domain |
| **HSP90AB1** | heat shock protein 90kDa alpha (cytosolic), class B member 1 | **TCF3** | transcription factor 3 |
| **MSH6** | mutS homolog 6 | **GNAQ** | guanine nucleotide binding protein (G protein), q polypeptide |
| **TBL1XR1** | transducin (beta)-like 1 X-linked receptor 1 | **PAX8** | paired box 8 |
| **BAP1** | BRCA1 associated protein-1 (ubiquitin carboxy-terminal hydrolase) | **PIK3R1** | phosphoinositide-3-kinase, regulatory subunit 1 (alpha) |
| **BCL11A** | B-cell CLL/lymphoma 11A (zinc finger protein) | **BRCA2** | breast cancer 2, early onset |
| **CASC5** | cancer susceptibility candidate 5 | **FGFR1** | fibroblast growth factor receptor 1 |
| **MET** | MET proto-oncogene, receptor tyrosine kinase | **SDC4** | syndecan 4 |
| **CEP89** | centrosomal protein 89kDa | **TBL1XR1** | transducin (beta)-like 1 X-linked receptor 1 |
| **TRIM33** | tripartite motif containing 33 | **FOXO3** | forkhead box O3 |
| **NOTCH1** | notch 1 | **CCND3** | cyclin D3 |
| **KIAA1549** | KIAA1549 | **FGFR4** | fibroblast growth factor receptor 4 |
| **DNM2** | dynamin 2 | **BCL7A** | B-cell CLL/lymphoma 7A |
| **CHN1** | chimerin 1 | **ZMYM2** | zinc finger, MYM-type 2 |
| **SETBP1** | SET binding protein 1 | **CCND1** | cyclin D1 |
| **WRN** | Werner syndrome, RecQ helicase-like | **JAK1** | Janus kinase 1 |
| **NR4A3** | nuclear receptor subfamily 4, group A, member 3 | **BAP1** | BRCA1 associated protein-1 (ubiquitin carboxy-terminal hydrolase) |
| **MLLT10** | myeloid/lymphoid or mixed-lineage leukemia (trithorax homolog, Drosophila); translocated to, 10 | **NAB2** | NGFI-A binding protein 2 (EGR1 binding protein 2) |
| **KDM5C** | lysine (K)-specific demethylase 5C | **LCK** | LCK proto-oncogene, Src family tyrosine kinase |
| **ETV6** | ets variant 6 | **CSF3R** | colony stimulating factor 3 receptor (granulocyte) |
| **ABL2** | ABL proto-oncogene 2, non-receptor tyrosine kinase | **HOXA11** | homeobox A11 |
| **RAD21** | RAD21 homolog (S. pombe) | **ZNF384** | zinc finger protein 384 |
| **PMS2** | PMS2 postmeiotic segregation increased 2 (S. cerevisiae) | **PPP2R1A** | protein phosphatase 2, regulatory subunit A, alpha |
| **XPC** | xeroderma pigmentosum, complementation group C | **SDHB** | succinate dehydrogenase complex, subunit B, iron sulfur (Ip) |
| **ERCC5** | excision repair cross-complementation group 5 | **BCR** | breakpoint cluster region |
| **PRDM1** | PR domain containing 1, with ZNF domain | **ERBB4** | erb-b2 receptor tyrosine kinase 4 |
| **FOXO4** | forkhead box O4 | **SFPQ** | splicing factor proline/glutamine-rich |
| **SPOP** | speckle-type POZ protein | **NTRK3** | neurotrophic tyrosine kinase, receptor, type 3 |
| **STAT3** | signal transducer and activator of transcription 3 (acute-phase response factor) | **SMARCB1** | SWI/SNF related, matrix associated, actin dependent regulator of chromatin, subfamily b, member 1 |
| **TFE3** | transcription factor binding to IGHM enhancer 3 | **TCEA1** | transcription elongation factor A (SII), 1 |
| **ITK** | IL2-inducible T-cell kinase | **MAP2K4** | mitogen-activated protein kinase kinase 4 |
| **NCOA2** | nuclear receptor coactivator 2 | **HOXC11** | homeobox C11 |
| **RBM10** | RNA binding motif protein 10 | **NCOR2** | nuclear receptor corepressor 2 |
| **ERCC3** | excision repair cross-complementation group 3 | **HSP90AA1** | heat shock protein 90kDa alpha (cytosolic), class A member 1 |
| **EPS15** | epidermal growth factor receptor pathway substrate 15 | **NUTM2A** | NUT family member 2A |
| **ZCCHC8** | zinc finger, CCHC domain containing 8 | **NRG1** | neuregulin 1 |
| **TSC1** | tuberous sclerosis 1 | **KMT2A** | lysine (K)-specific methyltransferase 2A |
| **ERC1** | ELKS/RAB6-interacting/CAST family member 1 | **NUTM1** | NUT midline carcinoma, family member 1 |
| **NFATC2** | nuclear factor of activated T-cells, cytoplasmic, calcineurin-dependent 2 | **EIF3E** | eukaryotic translation initiation factor 3, subunit E |
| **KDR** | kinase insert domain receptor | **HOXD13** | homeobox D13 |
| **DNMT3A** | DNA (cytosine-5-)-methyltransferase 3 alpha | **NFE2L2** | nuclear factor, erythroid 2-like 2 |
| **TRIM24** | tripartite motif containing 24 | **NRAS** | neuroblastoma RAS viral (v-ras) oncogene homolog |
| **PDGFRB** | platelet-derived growth factor receptor, beta polypeptide | **PDGFRA** | platelet-derived growth factor receptor, alpha polypeptide |
| **TPM3** | tropomyosin 3 | **DDX10** | DEAD (Asp-Glu-Ala-Asp) box polypeptide 10 |
| **FOXP1** | forkhead box P1 | **RSPO2** | R-spondin 2 |
| **TCF12** | transcription factor 12 | **RNF213** | ring finger protein 213 |
| **KIT** | v-kit Hardy-Zuckerman 4 feline sarcoma viral oncogene homolog | **ELN** | elastin |
| **CNOT3** | CCR4-NOT transcription complex, subunit 3 | **CXCR4** | chemokine (C-X-C motif) receptor 4 |
| **EXT2** | exostosin glycosyltransferase 2 | **CLTCL1** | clathrin, heavy chain-like 1 |
| **PCSK7** | proprotein convertase subtilisin/kexin type 7 | **BMPR1A** | bone morphogenetic protein receptor, type IA |
| **SS18** | synovial sarcoma translocation, chromosome 18 | **TET1** | tet methylcytosine dioxygenase 1 |
| **CARS** | cysteinyl-tRNA synthetase | **C2orf44** | chromosome 2 open reading frame 44 |
| **NUP214** | nucleoporin 214kDa | **RB1** | retinoblastoma 1 |
| **TSHR** | thyroid stimulating hormone receptor | **ASXL1** | additional sex combs like transcriptional regulator 1 |
| **BRD4** | bromodomain containing 4 | **MAML2** | mastermind-like 2 (Drosophila) |
| **PML** | promyelocytic leukemia | **NTRK1** | neurotrophic tyrosine kinase, receptor, type 1 |
| **JAK2** | Janus kinase 2 | **GMPS** | guanine monphosphate synthase |
| **FOXO3** | forkhead box O3 | **TNFAIP3** | tumor necrosis factor, alpha-induced protein 3 |
| **NTRK3** | neurotrophic tyrosine kinase, receptor, type 3 | **MYO5A** | myosin VA (heavy chain 12, myoxin) |
| **NCOR2** | nuclear receptor corepressor 2 | **SRSF3** | serine/arginine-rich splicing factor 3 |
| **RPL10** | ribosomal protein L10 | **KIAA1598** | KIAA1598 |
| **ARHGEF12** | Rho guanine nucleotide exchange factor (GEF) 12 | **NIN** | ninein (GSK3B interacting protein) |
| **ERCC2** | excision repair cross-complementation group 2 | **MYH11** | myosin, heavy chain 11, smooth muscle |
| **CCND2** | cyclin D2 | **SDHAF2** | succinate dehydrogenase complex assembly factor 2 |
| **USP6** | ubiquitin specific peptidase 6 | **RPL22** | ribosomal protein L22 |
| **THRAP3** | thyroid hormone receptor associated protein 3 | **TBX3** | T-box 3 |
| **MSN** | moesin | **CASC5** | cancer susceptibility candidate 5 |
| **AR** | androgen receptor | **HOXA13** | homeobox A13 |
| **MLLT3** | myeloid/lymphoid or mixed-lineage leukemia (trithorax homolog, Drosophila); translocated to, 3 | **KTN1** | kinectin 1 (kinesin receptor) |
| **PALB2** | partner and localizer of BRCA2 | **UBR5** | ubiquitin protein ligase E3 component n-recognin 5 |
| **EBF1** | early B-cell factor 1 | **MLF1** | myeloid leukemia factor 1 |
| **PER1** | period circadian clock 1 | **ELK4** | ELK4, ETS-domain protein (SRF accessory protein 1) |
| **HIP1** | huntingtin interacting protein 1 | **IL6ST** | interleukin 6 signal transducer |
| **PCM1** | pericentriolar material 1 | **MLLT1** | myeloid/lymphoid or mixed-lineage leukemia (trithorax homolog, Drosophila); translocated to, 1 |
| **RNF43** | ring finger protein 43 | **TLX3** | T-cell leukemia homeobox 3 |
| **RABEP1** | rabaptin, RAB GTPase binding effector protein 1 | **HLA-A** | major histocompatibility complex, class I, A |
| **RET** | ret proto-oncogene | **LMO1** | LIM domain only 1 (rhombotin 1) |
| **PMS1** | PMS1 postmeiotic segregation increased 1 (S. cerevisiae) | **STRN** | striatin, calmodulin binding protein |
| **sept-06** | septin 6 | **ATP1A1** | ATPase, Na+/K+ transporting, alpha 1 polypeptide |
| **PAX3** | paired box 3 | **SPOP** | speckle-type POZ protein |
| **MYB** | v-myb avian myeloblastosis viral oncogene homolog | **H3F3B** | H3 histone, family 3B (H3.3B) |
| **CHEK2** | checkpoint kinase 2 | **PHF6** | PHD finger protein 6 |
| **BCL11B** | B-cell CLL/lymphoma 11B (zinc finger protein) | **ESR1** | estrogen receptor 1 |
| **RAF1** | Raf-1 proto-oncogene, serine/threonine kinase | **STAT3** | signal transducer and activator of transcription 3 (acute-phase response factor) |
| **PSIP1** | PC4 and SFRS1 interacting protein 1 | **WWTR1** | WW domain containing transcription regulator 1 |
| **FANCA** | Fanconi anemia, complementation group A | **CCNB1IP1** | cyclin B1 interacting protein 1, E3 ubiquitin protein ligase |
| **WHSC1** | Wolf-Hirschhorn syndrome candidate 1 | **EXT1** | exostosin glycosyltransferase 1 |
| **IL7R** | interleukin 7 receptor | **SMARCA4** | SWI/SNF related, matrix associated, actin dependent regulator of chromatin, subfamily a, member 4 |
| **EWSR1** | EWS RNA-binding protein 1 | **MLLT11** | myeloid/lymphoid or mixed-lineage leukemia (trithorax homolog, Drosophila); translocated to, 11 |
| **HSP90AA1** | heat shock protein 90kDa alpha (cytosolic), class A member 1 | **ACVR1** | activin A receptor, type I |
| **IL6ST** | interleukin 6 signal transducer | **KDM6A** | lysine (K)-specific demethylase 6A |
| **RPL5** | ribosomal protein L5 | **DDX5** | DEAD (Asp-Glu-Ala-Asp) box helicase 5 |
| **PPFIBP1** | PTPRF interacting protein, binding protein 1 (liprin beta 1) | **RPL10** | ribosomal protein L10 |
| **PRRX1** | paired related homeobox 1 | **HOXD11** | homeobox D11 |
| **SMAD2** | SMAD family member 2 | **SLC45A3** | solute carrier family 45, member 3 |
| **MEN1** | multiple endocrine neoplasia I | **STK11** | serine/threonine kinase 11 |
| **KIF5B** | kinesin family member 5B | **GRIN2A** | glutamate receptor, ionotropic, N-methyl D-aspartate 2A |
| **COL2A1** | collagen, type II, alpha 1 | **AXIN1** | axin 1 |
| **MLH1** | mutL homolog 1 | **NCOR1** | nuclear receptor corepressor 1 |
| **TGFBR2** | transforming growth factor, beta receptor II (70/80kDa) | **RHOH** | ras homolog family member H |
| **WAS** | Wiskott-Aldrich syndrome | **ZNF331** | zinc finger protein 331 |
| **SPECC1** | sperm antigen with calponin homology and coiled-coil domains 1 | **NPM1** | nucleophosmin (nucleolar phosphoprotein B23, numatrin) |
| **MKL1** | megakaryoblastic leukemia (translocation) 1 | **JAZF1** | JAZF zinc finger 1 |
| **LPP** | LIM domain containing preferred translocation partner in lipoma | **sept-09** | septin 9 |
| **ABL1** | ABL proto-oncogene 1, non-receptor tyrosine kinase | **GPC3** | glypican 3 |
| **RALGDS** | ral guanine nucleotide dissociation stimulator | **ARID1B** | AT rich interactive domain 1B (SWI1-like) |
| **FGFR1OP** | FGFR1 oncogene partner | **PICALM** | phosphatidylinositol binding clathrin assembly protein |
| **PRF1** | perforin 1 (pore forming protein) | **TRIM33** | tripartite motif containing 33 |
| **NACA** | nascent polypeptide-associated complex alpha subunit | **HMGA1** | high mobility group AT-hook 1 |
| **HIF1A** | hypoxia inducible factor 1, alpha subunit (basic helix-loop-helix transcription factor) | **CANT1** | calcium activated nucleotidase 1 |
| **ELL** | elongation factor RNA polymerase II | **ARHGEF12** | Rho guanine nucleotide exchange factor (GEF) 12 |
| **FUS** | FUS RNA binding protein | **RECQL4** | RecQ protein-like 4 |
| **EML4** | echinoderm microtubule associated protein like 4 | **AFF1** | AF4/FMR2 family, member 1 |
| **CBL** | Cbl proto-oncogene, E3 ubiquitin protein ligase | **ZFHX3** | zinc finger homeobox 3 |
| **CIITA** | class II, major histocompatibility complex, transactivator | **TFPT** | TCF3 (E2A) fusion partner (in childhood Leukemia) |
| **WT1** | Wilms tumor 1 | **KMT2C** | lysine (K)-specific methyltransferase 2C |
| **PPP2R1A** | protein phosphatase 2, regulatory subunit A, alpha | **RPL5** | ribosomal protein L5 |
| **NRG1** | neuregulin 1 | **RAD51B** | RAD51 paralog B |
| **NFE2L2** | nuclear factor, erythroid 2-like 2 | **GATA3** | GATA binding protein 3 |
| **ARHGAP26** | Rho GTPase activating protein 26 | **NCOA1** | nuclear receptor coactivator 1 |
| **KRAS** | Kirsten rat sarcoma viral oncogene homolog | **OMD** | osteomodulin |
| **EXT1** | exostosin glycosyltransferase 1 | **KLK2** | kallikrein-related peptidase 2 |
| **RECQL4** | RecQ protein-like 4 | **CARD11** | caspase recruitment domain family, member 11 |
| **DDB2** | damage-specific DNA binding protein 2, 48kDa | **ERCC2** | excision repair cross-complementation group 2 |
| **FLI1** | Fli-1 proto-oncogene, ETS transcription factor | **NBN** | nibrin |
| **PRKAR1A** | protein kinase, cAMP-dependent, regulatory, type I, alpha | **CREBBP** | CREB binding protein |
| **ETV1** | ets variant 1 | **TRRAP** | transformation/transcription domain-associated protein |
| **ETV5** | ets variant 5 | **SH3GL1** | SH3-domain GRB2-like 1 |
| **CDH11** | cadherin 11, type 2, OB-cadherin (osteoblast) | **CCND2** | cyclin D2 |
| **SMO** | smoothened, frizzled class receptor | **SPEN** | spen family transcriptional repressor |
| **TCF7L2** | transcription factor 7-like 2 (T-cell specific, HMG-box) | **MAP3K1** | mitogen-activated protein kinase kinase kinase 1, E3 ubiquitin protein ligase |
| **LRIG3** | leucine-rich repeats and immunoglobulin-like domains 3 | **CDK12** | cyclin-dependent kinase 12 |
| **MSH2** | mutS homolog 2 | **KIAA1549** | KIAA1549 |
| **SRGAP3** | SLIT-ROBO Rho GTPase activating protein 3 | **DDX6** | DEAD (Asp-Glu-Ala-Asp) box helicase 6 |
| **ELF4** | E74-like factor 4 (ets domain transcription factor) | **SMAD4** | SMAD family member 4 |
| **HEY1** | hes-related family bHLH transcription factor with YRPW motif 1 | **CHIC2** | cysteine-rich hydrophobic domain 2 |
| **HOOK3** | hook microtubule-tethering protein 3 | **MYC** | v-myc avian myelocytomatosis viral oncogene homolog |
| **ERG** | v-ets avian erythroblastosis virus E26 oncogene homolog | **SND1** | staphylococcal nuclease and tudor domain containing 1 |
| **EZH2** | enhancer of zeste 2 polycomb repressive complex 2 subunit | **BIRC3** | baculoviral IAP repeat containing 3 |
| **PTPRK** | protein tyrosine phosphatase, receptor type, K | **USP6** | ubiquitin specific peptidase 6 |
| **AXIN2** | axin 2 | **NUP98** | nucleoporin 98kDa |
| **NFIB** | nuclear factor I/B | **RMI2** | RecQ mediated genome instability 2 |
| **ERCC4** | excision repair cross-complementation group 4 | **PDCD1LG2** | programmed cell death 1 ligand 2 |
| **PPARG** | peroxisome proliferator-activated receptor gamma | **THRAP3** | thyroid hormone receptor associated protein 3 |
| **CDC73** | cell division cycle 73 | **KDSR** | 3-ketodihydrosphingosine reductase |
| **BRAF** | B-Raf proto-oncogene, serine/threonine kinase | **PRRX1** | paired related homeobox 1 |
| **USP8** | ubiquitin specific peptidase 8 | **CASP8** | caspase 8, apoptosis-related cysteine peptidase |
| **FLT3** | fms-related tyrosine kinase 3 | **PRKAR1A** | protein kinase, cAMP-dependent, regulatory, type I, alpha |
| **TERT** | telomerase reverse transcriptase | **STAT6** | signal transducer and activator of transcription 6, interleukin-4 induced |
| **FUBP1** | far upstream element (FUSE) binding protein 1 | **PRDM16** | PR domain containing 16 |
| **NF2** | neurofibromin 2 (merlin) | **CHN1** | chimerin 1 |
| **CSF3R** | colony stimulating factor 3 receptor (granulocyte) | **BRIP1** | BRCA1 interacting protein C-terminal helicase 1 |
| **SLC34A2** | solute carrier family 34 (type II sodium/phosphate cotransporter), member 2 | **SET** | SET nuclear proto-oncogene |
| **SFPQ** | splicing factor proline/glutamine-rich | **IRF4** | interferon regulatory factor 4 |
| **NUTM1** | NUT midline carcinoma, family member 1 | **ARNT** | aryl hydrocarbon receptor nuclear translocator |
| **EIF3E** | eukaryotic translation initiation factor 3, subunit E | **ETV1** | ets variant 1 |
| **ELN** | elastin | **ATP2B3** | ATPase, Ca++ transporting, plasma membrane 3 |
| **CLTCL1** | clathrin, heavy chain-like 1 | **U2AF1** | U2 small nuclear RNA auxiliary factor 1 |
| **NTRK1** | neurotrophic tyrosine kinase, receptor, type 1 | **ETV5** | ets variant 5 |
| **ACSL6** | acyl-CoA synthetase long-chain family member 6 | **LMNA** | lamin A/C |
| **PAX5** | paired box 5 | **RANBP17** | RAN binding protein 17 |
| **SLC45A3** | solute carrier family 45, member 3 | **PAX7** | paired box 7 |
| **AXIN1** | axin 1 | **DEK** | DEK proto-oncogene |
| **PICALM** | phosphatidylinositol binding clathrin assembly protein | **NCOA2** | nuclear receptor coactivator 2 |
| **CANT1** | calcium activated nucleotidase 1 | **KAT6B** | K(lysine) acetyltransferase 6B |
| **NCOA1** | nuclear receptor coactivator 1 | **NFKB2** | nuclear factor of kappa light polypeptide gene enhancer in B-cells 2 (p49/p100) |
| **NBN** | nibrin | **GAS7** | growth arrest-specific 7 |
| **CTNNB1** | catenin (cadherin-associated protein), beta 1, 88kDa | **FSTL3** | follistatin-like 3 (secreted glycoprotein) |
| **NFKB2** | nuclear factor of kappa light polypeptide gene enhancer in B-cells 2 (p49/p100) | **CDKN2A** | cyclin-dependent kinase inhibitor 2A |
| **HLF** | hepatic leukemia factor | **MDS2** | myelodysplastic syndrome 2 translocation associated |
| **STAT5B** | signal transducer and activator of transcription 5B | **ATIC** | 5-aminoimidazole-4-carboxamide ribonucleotide formyltransferase/IMP cyclohydrolase |
| **STIL** | SCL/TAL1 interrupting locus | **CACNA1D** | calcium channel, voltage-dependent, L type, alpha 1D subunit |
| **HMGA2** | high mobility group AT-hook 2 | **CREB3L2** | cAMP responsive element binding protein 3-like 2 |
| **PRCC** | papillary renal cell carcinoma (translocation-associated) | **ABI1** | abl-interactor 1 |
| **FIP1L1** | factor interacting with PAPOLA and CPSF1 | **NCKIPSD** | NCK interacting protein with SH3 domain |
| **TCL6** | T-cell leukemia/lymphoma 6 (non-protein coding) | **ERCC3** | excision repair cross-complementation group 3 |
| **IKZF1** | IKAROS family zinc finger 1 (Ikaros) | **MLLT3** | myeloid/lymphoid or mixed-lineage leukemia (trithorax homolog, Drosophila); translocated to, 3 |
| **MALT1** | MALT1 paracaspase | **MNX1** | motor neuron and pancreas homeobox 1 |
| **SMAD3** | SMAD family member 3 | **MN1** | meningioma (disrupted in balanced translocation) 1 |
| **RBM15** | RNA binding motif protein 15 | **WHSC1L1** | Wolf-Hirschhorn syndrome candidate 1-like 1 |
| **sept-05** | septin 5 | **BCL2** | B-cell CLL/lymphoma 2 |
| **LZTR1** | leucine-zipper-like transcription regulator 1 | **SS18L1** | synovial sarcoma translocation gene on chromosome 18-like 1 |
| **ZBTB16** | zinc finger and BTB domain containing 16 | **ZCCHC8** | zinc finger, CCHC domain containing 8 |
| **TFRC** | transferrin receptor | **WRN** | Werner syndrome, RecQ helicase-like |
| **SUZ12** | SUZ12 polycomb repressive complex 2 subunit | **TSC1** | tuberous sclerosis 1 |
| **LSM14A** | LSM14A, SCD6 homolog A (S. cerevisiae) | **PALB2** | partner and localizer of BRCA2 |
| **TNFRSF14** | tumor necrosis factor receptor superfamily, member 14 | **POLE** | polymerase (DNA directed), epsilon, catalytic subunit |
| **DAXX** | death-domain associated protein | **PHOX2B** | paired-like homeobox 2b |
| **FGFR4** | fibroblast growth factor receptor 4 | **PDE4DIP** | phosphodiesterase 4D interacting protein |
| **LCK** | LCK proto-oncogene, Src family tyrosine kinase | **KIF5B** | kinesin family member 5B |
| **HOXA11** | homeobox A11 | **SDHA** | succinate dehydrogenase complex, subunit A, flavoprotein (Fp) |
| **ZNF384** | zinc finger protein 384 | **GPHN** | gephyrin |
| **AKT2** | v-akt murine thymoma viral oncogene homolog 2 | **AP002884.2** | AP002884.2 |
| **FBXO11** | F-box protein 11 | **FLT4** | fms-related tyrosine kinase 4 |
| **RPN1** | ribophorin I | **SH2B3** | SH2B adaptor protein 3 |
| **ETNK1** | ethanolamine kinase 1 | **TCF7L2** | transcription factor 7-like 2 (T-cell specific, HMG-box) |
| **GPC3** | glypican 3 | **FNBP1** | formin binding protein 1 |
| **RAD51B** | RAD51 paralog B | **CRTC1** | CREB regulated transcription coactivator 1 |
| **DDX6** | DEAD (Asp-Glu-Ala-Asp) box helicase 6 | **PER1** | period circadian clock 1 |
| **SND1** | staphylococcal nuclease and tudor domain containing 1 | **SMARCE1** | SWI/SNF related, matrix associated, actin dependent regulator of chromatin, subfamily e, member 1 |
| **ARNT** | aryl hydrocarbon receptor nuclear translocator | **HIP1** | huntingtin interacting protein 1 |
| **PHOX2B** | paired-like homeobox 2b | **PLAG1** | pleiomorphic adenoma gene 1 |
| **FLT4** | fms-related tyrosine kinase 4 | **BLM** | Bloom syndrome, RecQ helicase-like |
| **GATA2** | GATA binding protein 2 | **IDH2** | isocitrate dehydrogenase 2 (NADP+), mitochondrial |
| **CIC** | capicua transcriptional repressor | **NF1** | neurofibromin 1 |
| **MITF** | microphthalmia-associated transcription factor | **MLLT4** | myeloid/lymphoid or mixed-lineage leukemia (trithorax homolog, Drosophila); translocated to, 4 |
| **MAP2K1** | mitogen-activated protein kinase kinase 1 | **JAK3** | Janus kinase 3 |
| **HNRNPA2B1** | heterogeneous nuclear ribonucleoprotein A2/B1 | **CLP1** | cleavage and polyadenylation factor I subunit 1 |
| **BRD3** | bromodomain containing 3 | **STAT5B** | signal transducer and activator of transcription 5B |
| **PRKACA** | protein kinase, cAMP-dependent, catalytic, alpha | **CIC** | capicua transcriptional repressor |
| **RHOA** | ras homolog family member A | **FOXO1** | forkhead box O1 |
| **IDH1** | isocitrate dehydrogenase 1 (NADP+), soluble | **KAT6A** | K(lysine) acetyltransferase 6A |
| **RARA** | retinoic acid receptor, alpha | **PCM1** | pericentriolar material 1 |
| **TRAF7** | TNF receptor-associated factor 7, E3 ubiquitin protein ligase | **NFKBIE** | nuclear factor of kappa light polypeptide gene enhancer in B-cells inhibitor, epsilon |
| **TOP1** | topoisomerase (DNA) I | **MUTYH** | mutY homolog |
| **NDRG1** | N-myc downstream regulated 1 | **RANBP2** | RAN binding protein 2 |
| **MYCL** | v-myc avian myelocytomatosis viral oncogene lung carcinoma derived homolog | **MITF** | microphthalmia-associated transcription factor |
| **CCNE1** | cyclin E1 | **FEV** | FEV (ETS oncogene family) |
| **GNA11** | guanine nucleotide binding protein (G protein), alpha 11 (Gq class) | **ERC1** | ELKS/RAB6-interacting/CAST family member 1 |
| **SSX1** | synovial sarcoma, X breakpoint 1 | **CD79B** | CD79b molecule, immunoglobulin-associated beta |
| **EZR** | ezrin | **RUNX1** | runt-related transcription factor 1 |
| **MUC1** | mucin 1, cell surface associated | **BCL6** | B-cell CLL/lymphoma 6 |
| **HNF1A** | HNF1 homeobox A | **RNF43** | ring finger protein 43 |
| **SRSF2** | serine/arginine-rich splicing factor 2 | **SUFU** | suppressor of fused homolog (Drosophila) |
| **ECT2L** | epithelial cell transforming 2 like | **ALDH2** | aldehyde dehydrogenase 2 family (mitochondrial) |
| **PAX8** | paired box 8 | **EIF4A2** | eukaryotic translation initiation factor 4A2 |
| **DDX10** | DEAD (Asp-Glu-Ala-Asp) box polypeptide 10 | **RABEP1** | rabaptin, RAB GTPase binding effector protein 1 |
| **KIAA1598** | KIAA1598 | **CRTC3** | CREB regulated transcription coactivator 3 |
| **FAM46C** | family with sequence similarity 46, member C | **MAP2K1** | mitogen-activated protein kinase kinase 1 |
| **KLK2** | kallikrein-related peptidase 2 | **FCGR2B** | Fc fragment of IgG, low affinity IIb, receptor (CD32) |
| **PRDM16** | PR domain containing 16 | **STIL** | SCL/TAL1 interrupting locus |
| **CREB3L1** | cAMP responsive element binding protein 3-like 1 | **JUN** | jun proto-oncogene |
| **KLF4** | Kruppel-like factor 4 (gut) | **COL2A1** | collagen, type II, alpha 1 |
| **GAS7** | growth arrest-specific 7 | **PLCG1** | phospholipase C, gamma 1 |
| **GOPC** | golgi-associated PDZ and coiled-coil motif containing | **TFEB** | transcription factor EB |
| **TPM4** | tropomyosin 4 | **PTPN13** | protein tyrosine phosphatase, non-receptor type 13 (APO-1/CD95 (Fas)-associated phosphatase) |
| **PLAG1** | pleiomorphic adenoma gene 1 | **PRCC** | papillary renal cell carcinoma (translocation-associated) |
| **CD79B** | CD79b molecule, immunoglobulin-associated beta | **HNRNPA2B1** | heterogeneous nuclear ribonucleoprotein A2/B1 |
| **FCGR2B** | Fc fragment of IgG, low affinity IIb, receptor (CD32) | **RET** | ret proto-oncogene |
| **FANCC** | Fanconi anemia, complementation group C | **HEY1** | hes-related family bHLH transcription factor with YRPW motif 1 |
| **FAM131B** | family with sequence similarity 131, member B | **ATF1** | activating transcription factor 1 |
| **HRAS** | Harvey rat sarcoma viral oncogene homolog | **PMS1** | PMS1 postmeiotic segregation increased 1 (S. cerevisiae) |
| **FCRL4** | Fc receptor-like 4 | **BRD3** | bromodomain containing 3 |
| **SMARCD1** | SWI/SNF related, matrix associated, actin dependent regulator of chromatin, subfamily d, member 1 | **KDM5A** | lysine (K)-specific demethylase 5A |
| **POU5F1** | POU class 5 homeobox 1 | **HOOK3** | hook microtubule-tethering protein 3 |
| **REL** | v-rel avian reticuloendotheliosis viral oncogene homolog | **ERG** | v-ets avian erythroblastosis virus E26 oncogene homolog |
| **PTPN11** | protein tyrosine phosphatase, non-receptor type 11 | **KDR** | kinase insert domain receptor |
| **ACKR3** | atypical chemokine receptor 3 | **MDM4** | MDM4, p53 regulator |
| **FH** | fumarate hydratase | **LHFP** | lipoma HMGIC fusion partner |
| **HOXC13** | homeobox C13 | **ALK** | anaplastic lymphoma receptor tyrosine kinase |
| **MAX** | MYC associated factor X | **FIP1L1** | factor interacting with PAPOLA and CPSF1 |
| **PWWP2A** | PWWP domain containing 2A | **DNMT3A** | DNA (cytosine-5-)-methyltransferase 3 alpha |
| **PIM1** | Pim-1 proto-oncogene, serine/threonine kinase | **LASP1** | LIM and SH3 protein 1 |
| **MAFB** | v-maf avian musculoaponeurotic fibrosarcoma oncogene homolog B | **BCL9** | B-cell CLL/lymphoma 9 |
| **ASPSCR1** | alveolar soft part sarcoma chromosome region, candidate 1 | **FAM131B** | family with sequence similarity 131, member B |
| **LCP1** | lymphocyte cytosolic protein 1 (L-plastin) | **PATZ1** | POZ (BTB) and AT hook containing zinc finger 1 |
| **TCF3** | transcription factor 3 | **TRIM24** | tripartite motif containing 24 |
| **CCND3** | cyclin D3 | **HRAS** | Harvey rat sarcoma viral oncogene homolog |
| **NAB2** | NGFI-A binding protein 2 (EGR1 binding protein 2) | **EZH2** | enhancer of zeste 2 polycomb repressive complex 2 subunit |
| **TCEA1** | transcription elongation factor A (SII), 1 | **RHOA** | ras homolog family member A |
| **CXCR4** | chemokine (C-X-C motif) receptor 4 | **SPECC1** | sperm antigen with calponin homology and coiled-coil domains 1 |
| **STRN** | striatin, calmodulin binding protein | **SYK** | spleen tyrosine kinase |
| **CCNB1IP1** | cyclin B1 interacting protein 1, E3 ubiquitin protein ligase | **RUNX1T1** | runt-related transcription factor 1; translocated to, 1 (cyclin D-related) |
| **NONO** | non-POU domain containing, octamer-binding | **PAX3** | paired box 3 |
| **ZNF331** | zinc finger protein 331 | **MKL1** | megakaryoblastic leukemia (translocation) 1 |
| **SEPT09** | septin 9 | **SOX2** | SRY (sex determining region Y)-box 2 |
| **FANCG** | Fanconi anemia, complementation group G | **MTCP1** | mature T-cell proliferation 1 |
| **PAX7** | paired box 7 | **CUX1** | cut-like homeobox 1 |
| **GOLGA5** | golgin A5 | **TPM3** | tropomyosin 3 |
| **CREB3L2** | cAMP responsive element binding protein 3-like 2 | **FCRL4** | Fc receptor-like 4 |
| **ABI1** | abl-interactor 1 | **AXIN2** | axin 2 |
| **NCKIPSD** | NCK interacting protein with SH3 domain | **RAP1GDS1** | RAP1, GTP-GDP dissociation stimulator 1 |
| **GPHN** | gephyrin | **FOXP1** | forkhead box P1 |
| **FNBP1** | formin binding protein 1 | **TRIM27** | tripartite motif containing 27 |
| **BCL6** | B-cell CLL/lymphoma 6 | **PBX1** | pre-B-cell leukemia homeobox 1 |
| **JUN** | jun proto-oncogene | **BCL10** | B-cell CLL/lymphoma 10 |
| **LASP1** | LIM and SH3 protein 1 | **FANCE** | Fanconi anemia, complementation group E |
| **SYK** | spleen tyrosine kinase | **TCF12** | transcription factor 12 |
| **TFG** | TRK-fused gene | **LEF1** | lymphoid enhancer-binding factor 1 |
| **RAP1GDS1** | RAP1, GTP-GDP dissociation stimulator 1 | **SDHC** | succinate dehydrogenase complex, subunit C, integral membrane protein, 15kDa |
| **BCL10** | B-cell CLL/lymphoma 10 | **POU2AF1** | POU class 2 associating factor 1 |
| **TTL** | tubulin tyrosine ligase | **CDK6** | cyclin-dependent kinase 6 |
| **BUB1B** | BUB1 mitotic checkpoint serine/threonine kinase B | **C15orf65** | chromosome 15 open reading frame 65 |
| **MSI2** | musashi RNA-binding protein 2 | **AFF3** | AF4/FMR2 family, member 3 |
| **CREB1** | cAMP responsive element binding protein 1 | **NFIB** | nuclear factor I/B |
| **MAP2K2** | mitogen-activated protein kinase kinase 2 | **KIT** | v-kit Hardy-Zuckerman 4 feline sarcoma viral oncogene homolog |
| **PPP6C** | protein phosphatase 6, catalytic subunit | **IKBKB** | inhibitor of kappa light polypeptide gene enhancer in B-cells, kinase beta |
| **FANCF** | Fanconi anemia, complementation group F | **LPP** | LIM domain containing preferred translocation partner in lipoma |
| **MDM2** | MDM2 proto-oncogene, E3 ubiquitin protein ligase | **ABL1** | ABL proto-oncogene 1, non-receptor tyrosine kinase |
| **PDGFB** | platelet-derived growth factor beta polypeptide | **COL1A1** | collagen, type I, alpha 1 |
| **CD274** | CD274 molecule | **CEBPA** | CCAAT/enhancer binding protein (C/EBP), alpha |
| **MYD88** | myeloid differentiation primary response 88 | **IDH1** | isocitrate dehydrogenase 1 (NADP+), soluble |
| **CCDC6** | coiled-coil domain containing 6 | **SMARCD1** | SWI/SNF related, matrix associated, actin dependent regulator of chromatin, subfamily d, member 1 |
| **BCL7A** | B-cell CLL/lymphoma 7A | **RARA** | retinoic acid receptor, alpha |
| **CD79A** | CD79a molecule, immunoglobulin-associated alpha | **KMT2D** | lysine (K)-specific methyltransferase 2D |
| **POT1** | protection of telomeres 1 | **POU5F1** | POU class 5 homeobox 1 |
| **NUTM2A** | NUT family member 2A | **H3F3A** | H3 histone, family 3A |
| **RSPO2** | R-spondin 2 | **PBRM1** | polybromo 1 |
| **GMPS** | guanine monphosphate synthase | **SETD2** | SET domain containing 2 |
| **SRSF3** | serine/arginine-rich splicing factor 3 | **CHCHD7** | coiled-coil-helix-coiled-coil-helix domain containing 7 |
| **HOXA13** | homeobox A13 | **ACSL3** | acyl-CoA synthetase long-chain family member 3 |
| **MLF1** | myeloid leukemia factor 1 | **ETV4** | ets variant 4 |
| **H3F3B** | H3 histone, family 3B (H3.3B) | **MYB** | v-myb avian myeloblastosis viral oncogene homolog |
| **BCL3** | B-cell CLL/lymphoma 3 | **TTL** | tubulin tyrosine ligase |
| **WWTR1** | WW domain containing transcription regulator 1 | **YWHAE** | tyrosine 3-monooxygenase/tryptophan 5-monooxygenase activation protein, epsilon |
| **HERPUD1** | homocysteine-inducible, endoplasmic reticulum stress-inducible, ubiquitin-like domain member 1 | **MPL** | MPL proto-oncogene, thrombopoietin receptor |
| **RHOH** | ras homolog family member H | **MAF** | v-maf avian musculoaponeurotic fibrosarcoma oncogene homolog |
| **TFPT** | TCF3 (E2A) fusion partner (in childhood Leukemia) | **ERCC4** | excision repair cross-complementation group 4 |
| **OMD** | osteomodulin | **BUB1B** | BUB1 mitotic checkpoint serine/threonine kinase B |
| **MYC** | v-myc avian myelocytomatosis viral oncogene homolog | **EP300** | E1A binding protein p300 |
| **SET** | SET nuclear proto-oncogene | **PTK6** | protein tyrosine kinase 6 |
| **LMNA** | lamin A/C | **CNOT3** | CCR4-NOT transcription complex, subunit 3 |
| **DEK** | DEK proto-oncogene | **TOP1** | topoisomerase (DNA) I |
| **ATIC** | 5-aminoimidazole-4-carboxamide ribonucleotide formyltransferase/IMP cyclohydrolase |  |  |
| **MN1** | meningioma (disrupted in balanced translocation) 1 |  |  |
| **BCL2** | B-cell CLL/lymphoma 2 |  |  |
| **WIF1** | WNT inhibitory factor 1 |  |  |
| **SDHA** | succinate dehydrogenase complex, subunit A, flavoprotein (Fp) |  |  |
| **SH2B3** | SH2B adaptor protein 3 |  |  |
| **SMARCE1** | SWI/SNF related, matrix associated, actin dependent regulator of chromatin, subfamily e, member 1 |  |  |
| **SUFU** | suppressor of fused homolog (Drosophila) |  |  |
| **ATF1** | activating transcription factor 1 |  |  |
| **MDM4** | MDM4, p53 regulator |  |  |
| **SOX2** | SRY (sex determining region Y)-box 2 |  |  |
| **SDHC** | succinate dehydrogenase complex, subunit C, integral membrane protein, 15kDa |  |  |
| **H3F3A** | H3 histone, family 3A |  |  |
| **ACSL3** | acyl-CoA synthetase long-chain family member 3 |  |  |
| **MAF** | v-maf avian musculoaponeurotic fibrosarcoma oncogene homolog |  |  |
| **FLCN** | folliculin |  |  |
| **KCNJ5** | potassium channel, inwardly rectifying subfamily J, member 5 |  |  |
| **SBDS** | Shwachman-Bodian-Diamond syndrome |  |  |
| **TAL1** | T-cell acute lymphocytic leukemia 1 |  |  |
| **MYCN** | v-myc avian myelocytomatosis viral oncogene neuroblastoma derived homolog |  |  |
| **DDIT3** | DNA-damage-inducible transcript 3 |  |  |
| **TNFRSF17** | tumor necrosis factor receptor superfamily, member 17 |  |  |
| **SDC4** | syndecan 4 |  |  |
| **SDHB** | succinate dehydrogenase complex, subunit B, iron sulfur (Ip) |  |  |
| **SMARCB1** | SWI/SNF related, matrix associated, actin dependent regulator of chromatin, subfamily b, member 1 |  |  |
| **HOXD13** | homeobox D13 |  |  |
| **BMPR1A** | bone morphogenetic protein receptor, type IA |  |  |
| **P2RY8** | purinergic receptor P2Y, G-protein coupled, 8 |  |  |
| **RPL22** | ribosomal protein L22 |  |  |
| **PHF6** | PHD finger protein 6 |  |  |
| **STK11** | serine/threonine kinase 11 |  |  |
| **DNAJB1** | DnaJ (Hsp40) homolog, subfamily B, member 1 |  |  |
| **CHIC2** | cysteine-rich hydrophobic domain 2 |  |  |
| **BIRC3** | baculoviral IAP repeat containing 3 |  |  |
| **GATA1** | GATA binding protein 1 (globin transcription factor 1) |  |  |
| **IRF4** | interferon regulatory factor 4 |  |  |
| **SS18L1** | synovial sarcoma translocation gene on chromosome 18-like 1 |  |  |
| **MUTYH** | mutY homolog |  |  |
| **CRTC3** | CREB regulated transcription coactivator 3 |  |  |
| **TFEB** | transcription factor EB |  |  |
| **LHFP** | lipoma HMGIC fusion partner |  |  |
| **PATZ1** | POZ (BTB) and AT hook containing zinc finger 1 |  |  |
| **PBX1** | pre-B-cell leukemia homeobox 1 |  |  |
| **FANCE** | Fanconi anemia, complementation group E |  |  |
| **LEF1** | lymphoid enhancer-binding factor 1 |  |  |
| **CDK6** | cyclin-dependent kinase 6 |  |  |
| **C15orf65** | chromosome 15 open reading frame 65 |  |  |
| **MPL** | MPL proto-oncogene, thrombopoietin receptor |  |  |
| **PTK6** | protein tyrosine kinase 6 |  |  |
| **TAL2** | T-cell acute lymphocytic leukemia 2 |  |  |
| **LYL1** | lymphoblastic leukemia associated hematopoiesis regulator 1 |  |  |
| **CDX2** | caudal type homeobox 2 |  |  |
| **FAS** | Fas cell surface death receptor |  |  |
| **RAC1** | ras-related C3 botulinum toxin substrate 1 (rho family, small GTP binding protein Rac1) |  |  |
| **FHIT** | fragile histidine triad |  |  |
| **KLF6** | Kruppel-like factor 6 |  |  |
| **FGFR3** | fibroblast growth factor receptor 3 |  |  |
| **HOXA9** | homeobox A9 |  |  |
| **GNAQ** | guanine nucleotide binding protein (G protein), q polypeptide |  |  |
| **CCND1** | cyclin D1 |  |  |
| **CALR** | calreticulin |  |  |
| **HOXC11** | homeobox C11 |  |  |
| **NRAS** | neuroblastoma RAS viral (v-ras) oncogene homolog |  |  |
| **IL21R** | interleukin 21 receptor |  |  |
| **C2orf44** | chromosome 2 open reading frame 44 |  |  |
| **SDHAF2** | succinate dehydrogenase complex assembly factor 2 |  |  |
| **ELK4** | ELK4, ETS-domain protein (SRF accessory protein 1) |  |  |
| **LMO2** | LIM domain only 2 (rhombotin-like 1) |  |  |
| **MLLT1** | myeloid/lymphoid or mixed-lineage leukemia (trithorax homolog, Drosophila); translocated to, 1 |  |  |
| **HLA-A** | major histocompatibility complex, class I, A |  |  |
| **LMO1** | LIM domain only 1 (rhombotin 1) |  |  |
| **ACVR1** | activin A receptor, type I |  |  |
| **NPM1** | nucleophosmin (nucleolar phosphoprotein B23, numatrin) |  |  |
| **HMGA1** | high mobility group AT-hook 1 |  |  |
| **SH3GL1** | SH3-domain GRB2-like 1 |  |  |
| **MDS2** | myelodysplastic syndrome 2 translocation associated |  |  |
| **MNX1** | motor neuron and pancreas homeobox 1 |  |  |
| **FOXO1** | forkhead box O1 |  |  |
| **NFKBIE** | nuclear factor of kappa light polypeptide gene enhancer in B-cells inhibitor, epsilon |  |  |
| **FEV** | FEV (ETS oncogene family) |  |  |
| **IL2** | interleukin 2 |  |  |
| **RSPO3** | R-spondin 3 |  |  |
| **MTCP1** | mature T-cell proliferation 1 |  |  |
| **TRIM27** | tripartite motif containing 27 |  |  |
| **POU2AF1** | POU class 2 associating factor 1 |  |  |
| **CBLC** | Cbl proto-oncogene C, E3 ubiquitin protein ligase |  |  |
| **CHCHD7** | coiled-coil-helix-coiled-coil-helix domain containing 7 |  |  |
| **HMGN2P46** | high mobility group nucleosomal binding domain 2 pseudogene 46 |  |  |
| **SOCS1** | suppressor of cytokine signaling 1 |  |  |
| **COX6C** | cytochrome c oxidase subunit VIc |  |  |
| **VTI1A** | vesicle transport through interaction with t-SNAREs 1A |  |  |
| **NKX2-1** | NK2 homeobox 1 |  |  |
| **FOXL2** | forkhead box L2 |  |  |
| **PAFAH1B2** | platelet-activating factor acetylhydrolase 1b, catalytic subunit 2 (30kDa) |  |  |
| **SDHD** | succinate dehydrogenase complex, subunit D, integral membrane protein |  |  |
| **TMPRSS2** | transmembrane protease, serine 2 |  |  |
| **CBFA2T3** | core-binding factor, runt domain, alpha subunit 2; translocated to, 3 |  |  |
| **CD74** | CD74 molecule, major histocompatibility complex, class II invariant chain |  |  |
| **CDK4** | cyclin-dependent kinase 4 |  |  |
| **CNBP** | CCHC-type zinc finger, nucleic acid binding protein |  |  |
| **TLX3** | T-cell leukemia homeobox 3 |  |  |
| **HOXD11** | homeobox D11 |  |  |
| **JAZF1** | JAZF zinc finger 1 |  |  |
| **CDKN2C** | cyclin-dependent kinase inhibitor 2C (p18, inhibits CDK4) |  |  |
| **BTG1** | B-cell translocation gene 1, anti-proliferative |  |  |
| **PDCD1LG2** | programmed cell death 1 ligand 2 |  |  |
| **KDSR** | 3-ketodihydrosphingosine reductase |  |  |
| **SSX2** | synovial sarcoma, X breakpoint 2 |  |  |
| **U2AF1** | U2 small nuclear RNA auxiliary factor 1 |  |  |
| **NT5C2** | 5'-nucleotidase, cytosolic II |  |  |
| **ZRSR2** | zinc finger (CCCH type), RNA-binding motif and serine/arginine rich 2 |  |  |
| **CRTC1** | CREB regulated transcription coactivator 1 |  |  |
| **IDH2** | isocitrate dehydrogenase 2 (NADP+), mitochondrial |  |  |
| **CLP1** | cleavage and polyadenylation factor I subunit 1 |  |  |
| **ALDH2** | aldehyde dehydrogenase 2 family (mitochondrial) |  |  |
| **NUTM2B** | NUT family member 2B |  |  |
| **CEBPA** | CCAAT/enhancer binding protein (C/EBP), alpha |  |  |
| **YWHAE** | tyrosine 3-monooxygenase/tryptophan 5-monooxygenase activation protein, epsilon |  |  |
| **VHL** | von Hippel-Lindau tumor suppressor, E3 ubiquitin protein ligase |  |  |
| **OLIG2** | oligodendrocyte lineage transcription factor 2 |  |  |
| **MYOD1** | myogenic differentiation 1 |  |  |
| **MLLT11** | myeloid/lymphoid or mixed-lineage leukemia (trithorax homolog, Drosophila); translocated to, 11 |  |  |
| **RMI2** | RecQ mediated genome instability 2 |  |  |
| **FSTL3** | follistatin-like 3 (secreted glycoprotein) |  |  |
| **CDKN2A** | cyclin-dependent kinase inhibitor 2A |  |  |
| **XPA** | xeroderma pigmentosum, complementation group A |  |  |
| **AP002884.2** | AP002884.2 |  |  |
| **TLX1** | T-cell leukemia homeobox 1 |  |  |
| **TCL1A** | T-cell leukemia/lymphoma 1A |  |  |
| **CRLF2** | cytokine receptor-like factor 2 |  |  |
| **ETV4** | ets variant 4 |  |  |
