# Supplementary material for: Path to Clonal Theranostics in Luminal Breast Cancers
Source: Front Oncol. 2022 Jan 13;11:802177. doi: 10.3389/fonc.2021.802177 (PMC8793283; doi:10.3389/fonc.2021.802177)
Supplement: Supplementary Material 1 — TCGA database of mutations and CNV alterations in early and advanced breast cancers. [file DataSheet_1.zip › Data Sheet 11.doc]

**Supplementary Table 1**: Information concerning patients and type of tumors identified by the pathologist

| Sample ID | Tissue type | Stage | Patient ID* | Method of sample collection and concomitant treatment |
| --- | --- | --- | --- | --- |
| 1 | breast | EBC | 1 | surgery |
| 2 | breast | EBC | 2 | surgery |
| 3 | breast | EBC | 3 | surgery |
| 4 | breast | EBC | 4 | surgery ; chemotherapy |
| 5 | breast | EBC | 5 | surgery |
| 6 | breast | EBC | 6 | surgery |
| 7 | breast | EBC | 7 | surgery |
| 8 | breast | EBC | 8 | surgery |
| 9 | breast | EBC | 9 | surgery |
| 10 | breast | EBC | 10 | surgery |
| 11 | breast | EBC | 11 | surgery |
| 12 | breast | EBC | 12 | surgery |
| 13 | breast | EBC | 13 | surgery |
| 14 | breast | EBC | 14 | surgery |
| 15 | breast | EBC | 15 | surgery |
| 16 | breast | EBC | 16 | surgery |
| 17 | breast | EBC | 17 | surgery |
| 18 | breast | EBC | 18 | surgery |
| 19 | breast | EBC | 19 | surgery |
| 20 | breast | EBC | 20 | surgery |
| 21 | breast | EBC | 21 | surgery |
| 22 | breast | EBC | 22 | surgery |
| 23 | breast | EBC | 23 | surgery |
| 24 | breast | EBC | 24 | surgery |
| 25 | breast | EBC | 4 | surgery ; chemotherapy |
| 26 | breast | EBC | 25 | surgery |
| 27 | breast | EBC | 26 | surgery |
| 28 | breast | EBC | 27 | surgery |
| 29 | breast | EBC | 28 | surgery |
| 30 | breast | EBC | 29 | surgery |
| 31 | breast | EBC | 30 | surgery |
| 32 | breast | EBC | 31 | surgery |
| 33 | breast | EBC | 32 | surgery |
| 34 | breast | EBC | 33 | surgery |
| 35 | breast | EBC | 34 | surgery |
| 36 | breast | EBC | 35 | surgery |
| 37 | breast | EBC | 36 | surgery |
| 38 | breast | EBC | 37 | surgery |
| 39 | breast | EBC | 38 | surgery |
| 40 | breast | EBC | 39 | surgery |
| 41 | breast | EBC | 40 | surgery |
| 42 | breast | EBC | 41 | surgery |
| 43 | breast | EBC | 42 | surgery |
| 44 | breast | EBC | 43 | surgery |
| 45 | breast | EBC | 44 | surgery |
| 46 | breast | EBC | 45 | surgery |
| 47 | breast | EBC | 46 | surgery |
| 48 | breast | EBC | 47 | surgery |
| 49 | breast | EBC | 48 | surgery |
| 50 | breast | EBC | 49 | surgery |
| 51 | breast | EBC | 50 | surgery |
| 52 | breast | EBC | 51 | surgery |
| 53 | skin | ABC | 52 | surgical biopsy ; endocrine therapy |
| 54 | skin | ABC | 53 | surgical biopsy ; endocrine therapy |
| 55 | node | ABC | 53 | surgical biopsy ; endocrine therapy |
| 56 | skin | ABC | 54 | surgical biopsy ; endocrine therapy |
| 57 | node | ABC | 53 | surgical biopsy ; endocrine therapy |
| 58 | skin | ABC | 54 | surgical biopsy ; endocrine therapy |
| 59 | bone | ABC | 52 | FNB ; chemotherapy |
| 60 | liver | ABC | 39 | FNB |
| 61 | node | ABC | 55 | surgical biopsy ; chemotherapy |
| 62 | liver | ABC | 56 | FNB ; chemotherapy |
| 63 | skin | ABC | 57 | surgical biopsy ; endocrine therapy |
| 64 | liver | ABC | 39 | FNB ; chemotherapy |
| 65 | node | ABC | 56 | FNB ; chemotherapy |
| 66 | liver | ABC | 58 | FNB ; endocrine therapy |
| 67 | skin | ABC | 57 | surgical biopsy |
| 68 | skin | ABC | 4 | FNB ; chemotherapy |
| 69 | bone | ABC | 58 | FNB ; endocrine therapy |
| 70 | skin | ABC | 57 | FNB |
| 71 | liver | ABC | 59 | FNB ; endocrine therapy |
| 72 | node | ABC | 60 | FNB |
| 73 | skin | ABC | 57 | FNB |
| 74 | bone | ABC | 60 | FNB ; endocrine therapy |
| 75 | node | ABC | 17 | FNB ; chemotherapy |
| 76 | skin | ABC | 54 | FNB ; endocrine therapy |
| * Some patients had multiple sample collection through the course of the disease; ABC: advanced breast cancer; EBC: early breast cancer; FNB: fine needle biopsy | | | | |
