# Supplementary material for: Path to Clonal Theranostics in Luminal Breast Cancers
Source: Front Oncol. 2022 Jan 13;11:802177. doi: 10.3389/fonc.2021.802177 (PMC8793283; doi:10.3389/fonc.2021.802177)
Supplement: Supplementary Material 1 — TCGA database of mutations and CNV alterations in early and advanced breast cancers. [file DataSheet_1.zip › Data Sheet 2.doc]

**Supplementary material 2:** BC360 panel gene list

| **Gene Symbol** | **Panel BC 360 -Official Full Name** |
| --- | --- |
| **ABCA8** | ATP binding cassette subfamily A member 8 |
| **ACTR3B** | ARP3 actin related protein 3 homolog B |
| **ACVR1B** | activin A receptor type 1B |
| **ACVR1C** | activin A receptor type 1C |
| **ACVRL1** | activin A receptor like type 1 |
| **ADAM12** | ADAM metallopeptidase domain 12 |
| **ADCY9** | adenylate cyclase 9 |
| **ADD1** | adducin 1 |
| **ADM** | adrenomedullin |
| **AGR2** | anterior gradient 2, protein disulphide isomerase family member |
| **AGT** | angiotensinogen |
| **AGTR1** | angiotensin II receptor type 1 |
| **AKT3** | AKT serine/threonine kinase 3 |
| **ALDH1A1** | aldehyde dehydrogenase 1 family member A1 |
| **ALDOA** | aldolase, fructose-bisphosphate A |
| **ANGPT1** | angiopoietin 1 |
| **ANLN** | anillin actin binding protein |
| **ANXA9** | annexin A9 |
| **APH1B** | aph-1 homolog B, gamma-secretase subunit |
| **APOD** | apolipoprotein D |
| **APOE** | apolipoprotein E |
| **AR** | androgen receptor |
| **AREG** | amphiregulin |
| **ARID1A** | AT-rich interaction domain 1A |
| **ARNT2** | aryl hydrocarbon receptor nuclear translocator 2 |
| **ASPM** | abnormal spindle microtubule assembly |
| **ASPN** | asporin |
| **ATAD2** | ATPase family, AAA domain containing 2 |
| **ATM** | ATM serine/threonine kinase |
| **ATP10B** | ATPase phospholipid transporting 10B (putative) |
| **AURKA** | aurora kinase A |
| **AURKB** | aurora kinase B |
| **AXIN1** | axin 1 |
| **AXIN2** | axin 2 |
| **B3GNT3** | UDP-GlcNAc:betaGal beta-1,3-N-acetylglucosaminyltransferase 3 |
| **BAD** | BCL2 associated agonist of cell death |
| **BAG1** | BCL2 associated athanogene 1 |
| **BAIAP2L1** | BAI1 associated protein 2 like 1 |
| **BAIAP3** | BAI1 associated protein 3 |
| **BAMBI** | BMP and activin membrane bound inhibitor |
| **BAX** | BCL2 associated X, apoptosis regulator |
| **BBC3** | BCL2 binding component 3 |
| **BBOX1** | gamma-butyrobetaine hydroxylase 1 |
| **BCAS1** | breast carcinoma amplified sequence 1 |
| **BCL11A** | B-cell CLL/lymphoma 11A |
| **BCL2** | BCL2, apoptosis regulator |
| **BCL2A1** | BCL2 related protein A1 |
| **BCL2L1** | BCL2 like 1 |
| **BCL6B** | B-cell CLL/lymphoma 6B |
| **BDNF** | brain derived neurotrophic factor |
| **BIRC5** | baculoviral IAP repeat containing 5 |
| **BLM** | Bloom syndrome RecQ like helicase |
| **BLVRA** | biliverdin reductase A |
| **BMP2** | bone morphogenetic protein 2 |
| **BMP4** | bone morphogenetic protein 4 |
| **BMP5** | bone morphogenetic protein 5 |
| **BMP6** | bone morphogenetic protein 6 |
| **BMP7** | bone morphogenetic protein 7 |
| **BMP8A** | bone morphogenetic protein 8a |
| **BMPR1A** | bone morphogenetic protein receptor type 1A |
| **BMPR1B** | bone morphogenetic protein receptor type 1B |
| **BMPR2** | bone morphogenetic protein receptor type 2 |
| **BNC2** | basonuclin 2 |
| **BNIP3** | BCL2 interacting protein 3 |
| **BORCS7** | BLOC-1 related complex subunit 7 |
| **BRCA1** | BRCA1, DNA repair associated |
| **BRCA2** | BRCA2, DNA repair associated |
| **BTG2** | BTG anti-proliferation factor 2 |
| **C5orf38** | chromosome 5 open reading frame 38 |
| **CA12** | carbonic anhydrase 12 |
| **CACNA1D** | calcium voltage-gated channel subunit alpha1 D |
| **CACNA1H** | calcium voltage-gated channel subunit alpha1 H |
| **CACNA2D1** | calcium voltage-gated channel auxiliary subunit alpha2delta 1 |
| **CACNA2D3** | calcium voltage-gated channel auxiliary subunit alpha2delta 3 |
| **CACNG1** | calcium voltage-gated channel auxiliary subunit gamma 1 |
| **CACNG4** | calcium voltage-gated channel auxiliary subunit gamma 4 |
| **CACNG6** | calcium voltage-gated channel auxiliary subunit gamma 6 |
| **CALML5** | calmodulin like 5 |
| **CAMK2B** | calcium/calmodulin dependent protein kinase II beta |
| **CAV1** | caveolin 1 |
| **CBLC** | Cbl proto-oncogene C |
| **CCL2** | C-C motif chemokine ligand 2 |
| **CCL21** | C-C motif chemokine ligand 21 |
| **CCL3L1** | C-C motif chemokine ligand 3 like 1 |
| **CCL4** | C-C motif chemokine ligand 4 |
| **CCL5** | C-C motif chemokine ligand 5 |
| **CCL7** | C-C motif chemokine ligand 7 |
| **CCL8** | C-C motif chemokine ligand 8 |
| **CCNA1** | cyclin A1 |
| **CCNA2** | cyclin A2 |
| **CCNB1** | cyclin B1 |
| **CCND1** | cyclin D1 |
| **CCND2** | cyclin D2 |
| **CCNE1** | cyclin E1 |
| **CCNE2** | cyclin E2 |
| **CCR1** | C-C motif chemokine receptor 1 |
| **CCR2** | C-C motif chemokine receptor 2 |
| **CCR5** | C-C motif chemokine receptor 5 (gene/pseudogene) |
| **CD163** | CD163 molecule |
| **CD19** | CD19 molecule |
| **CD1E** | CD1e molecule |
| **CD24** | CD24 molecule |
| **CD27** | CD27 molecule |
| **CD274** | CD274 molecule |
| **CD276** | CD276 molecule |
| **CD34** | CD34 molecule |
| **CD36** | CD36 molecule |
| **CD44** | CD44 molecule (Indian blood group) |
| **CD68** | CD68 molecule |
| **CD84** | CD84 molecule |
| **CD8A** | CD8a molecule |
| **CD8B** | CD8b molecule |
| **CDC14A** | cell division cycle 14A |
| **CDC14B** | cell division cycle 14B |
| **CDC20** | cell division cycle 20 |
| **CDC25A** | cell division cycle 25A |
| **CDC25B** | cell division cycle 25B |
| **CDC25C** | cell division cycle 25C |
| **CDC6** | cell division cycle 6 |
| **CDC7** | cell division cycle 7 |
| **CDCA5** | cell division cycle associated 5 |
| **CDCA7L** | cell division cycle associated 7 like |
| **CDCA8** | cell division cycle associated 8 |
| **CDH1** | cadherin 1 |
| **CDH2** | cadherin 2 |
| **CDH3** | cadherin 3 |
| **CDH5** | cadherin 5 |
| **CDK1** | cyclin dependent kinase 1 |
| **CDK4** | cyclin dependent kinase 4 |
| **CDK6** | cyclin dependent kinase 6 |
| **CDKN1A** | cyclin dependent kinase inhibitor 1A |
| **CDKN1B** | cyclin dependent kinase inhibitor 1B |
| **CDKN1C** | cyclin dependent kinase inhibitor 1C |
| **CDKN2A** | cyclin dependent kinase inhibitor 2A |
| **CDKN2B** | cyclin dependent kinase inhibitor 2B |
| **CDKN2C** | cyclin dependent kinase inhibitor 2C |
| **CDKN2D** | cyclin dependent kinase inhibitor 2D |
| **CDKN3** | cyclin dependent kinase inhibitor 3 |
| **CEACAM5** | carcinoembryonic antigen related cell adhesion molecule 5 |
| **CEACAM6** | carcinoembryonic antigen related cell adhesion molecule 6 |
| **CENPF** | centromere protein F |
| **CEP55** | centrosomal protein 55 |
| **CETN2** | centrin 2 |
| **CFD** | complement factor D |
| **CHAD** | chondroadherin |
| **CHEK2** | checkpoint kinase 2 |
| **CHI3L1** | chitinase 3 like 1 |
| **CHIT1** | chitinase 1 |
| **CHRNA5** | cholinergic receptor nicotinic alpha 5 subunit |
| **CKB** | creatine kinase B |
| **CKMT1A** | creatine kinase, mitochondrial 1A |
| **CKS1B** | CDC28 protein kinase regulatory subunit 1B |
| **CLDN1** | claudin 1 |
| **CLDN3** | claudin 3 |
| **CLDN4** | claudin 4 |
| **CLDN7** | claudin 7 |
| **CLEC14A** | C-type lectin domain containing 14A |
| **CLEC5A** | C-type lectin domain containing 5A |
| **CMKLR1** | chemerin chemokine-like receptor 1 |
| **CNTFR** | ciliary neurotrophic factor receptor |
| **COL11A1** | collagen type XI alpha 1 chain |
| **COL27A1** | collagen type XXVII alpha 1 chain |
| **COL2A1** | collagen type II alpha 1 chain |
| **COL4A6** | collagen type IV alpha 6 chain |
| **COL6A3** | collagen type VI alpha 3 chain |
| **COL7A1** | collagen type VII alpha 1 chain |
| **COL9A3** | collagen type IX alpha 3 chain |
| **COLEC12** | collectin subfamily member 12 |
| **COMP** | cartilage oligomeric matrix protein |
| **CPA3** | carboxypeptidase A3 |
| **CREBBP** | CREB binding protein |
| **CRYAB** | crystallin alpha B |
| **CSF3R** | colony stimulating factor 3 receptor |
| **CTSW** | cathepsin W |
| **CXADR** | CXADR, Ig-like cell adhesion molecule |
| **CXCL10** | C-X-C motif chemokine ligand 10 |
| **CXCL12** | C-X-C motif chemokine ligand 12 |
| **CXCL13** | C-X-C motif chemokine ligand 13 |
| **CXCL5** | C-X-C motif chemokine ligand 5 |
| **CXCL8** | C-X-C motif chemokine ligand 8 |
| **CXCL9** | C-X-C motif chemokine ligand 9 |
| **CXCR6** | C-X-C motif chemokine receptor 6 |
| **CXorf36** | chromosome X open reading frame 36 |
| **CXXC5** | CXXC finger protein 5 |
| **CYBB** | cytochrome b-245 beta chain |
| **CYP4F3** | cytochrome P450 family 4 subfamily F member 3 |
| **DCN** | decorin |
| **DDB2** | damage specific DNA binding protein 2 |
| **DDR2** | discoidin domain receptor tyrosine kinase 2 |
| **DDX39A** | DExD-box helicase 39A |
| **DEPDC1** | DEP domain containing 1 |
| **DHRS2** | dehydrogenase/reductase 2 |
| **DKK1** | dickkopf WNT signaling pathway inhibitor 1 |
| **DKK2** | dickkopf WNT signaling pathway inhibitor 2 |
| **DLGAP5** | DLG associated protein 5 |
| **DLL1** | delta like canonical Notch ligand 1 |
| **DLL3** | delta like canonical Notch ligand 3 |
| **DLL4** | delta like canonical Notch ligand 4 |
| **DNAJC12** | DnaJ heat shock protein family (Hsp40) member C12 |
| **DPT** | dermatopontin |
| **DSC2** | desmocollin 2 |
| **DTX1** | deltex E3 ubiquitin ligase 1 |
| **DTX3** | deltex E3 ubiquitin ligase 3 |
| **DTX4** | deltex E3 ubiquitin ligase 4 |
| **DUSP4** | dual specificity phosphatase 4 |
| **DUSP6** | dual specificity phosphatase 6 |
| **E2F1** | E2F transcription factor 1 |
| **E2F5** | E2F transcription factor 5 |
| **ECM2** | extracellular matrix protein 2 |
| **EDN1** | endothelin 1 |
| **EDNRB** | endothelin receptor type B |
| **EFNA3** | ephrin A3 |
| **EFNA5** | ephrin A5 |
| **EGF** | epidermal growth factor |
| **EGFR** | epidermal growth factor receptor |
| **EGLN2** | egl-9 family hypoxia inducible factor 2 |
| **EGLN3** | egl-9 family hypoxia inducible factor 3 |
| **EIF2AK3** | eukaryotic translation initiation factor 2 alpha kinase 3 |
| **EIF3B** | eukaryotic translation initiation factor 3 subunit B |
| **EIF4E2** | eukaryotic translation initiation factor 4E family member 2 |
| **EIF4EBP1** | eukaryotic translation initiation factor 4E binding protein 1 |
| **ELF3** | E74 like ETS transcription factor 3 |
| **ELK3** | ELK3, ETS transcription factor |
| **ELOVL2** | ELOVL fatty acid elongase 2 |
| **EMCN** | endomucin |
| **ENO1** | enolase 1 |
| **ENPP2** | ectonucleotide pyrophosphatase/phosphodiesterase 2 |
| **EP300** | E1A binding protein p300 |
| **EPAS1** | endothelial PAS domain protein 1 |
| **ERBB2** | erb-b2 receptor tyrosine kinase 2 |
| **ERBB4** | erb-b2 receptor tyrosine kinase 4 |
| **ERCC1** | ERCC excision repair 1, endonuclease non-catalytic subunit |
| **EREG** | epiregulin |
| **ESPL1** | extra spindle pole bodies like 1, separase |
| **ESR1** | estrogen receptor 1 |
| **ETV4** | ETS variant 4 |
| **ETV7** | ETS variant 7 |
| **EXO1** | exonuclease 1 |
| **EYA1** | EYA transcriptional coactivator and phosphatase 1 |
| **EYA2** | EYA transcriptional coactivator and phosphatase 2 |
| **EYA4** | EYA transcriptional coactivator and phosphatase 4 |
| **F3** | coagulation factor III, tissue factor |
| **FAM124B** | family with sequence similarity 124 member B |
| **FAM198B** | family with sequence similarity 198 member B |
| **FAM214A** | family with sequence similarity 214 member A |
| **FAM83D** | family with sequence similarity 83 member D |
| **FANCF** | Fanconi anemia complementation group F |
| **FAP** | fibroblast activation protein alpha |
| **FBN1** | fibrillin 1 |
| **FGF1** | fibroblast growth factor 1 |
| **FGF10** | fibroblast growth factor 10 |
| **FGF12** | fibroblast growth factor 12 |
| **FGF13** | fibroblast growth factor 13 |
| **FGF18** | fibroblast growth factor 18 |
| **FGF2** | fibroblast growth factor 2 |
| **FGF7** | fibroblast growth factor 7 |
| **FGF9** | fibroblast growth factor 9 |
| **FGFR2** | fibroblast growth factor receptor 2 |
| **FGFR3** | fibroblast growth factor receptor 3 |
| **FGFR4** | fibroblast growth factor receptor 4 |
| **FGL2** | fibrinogen like 2 |
| **FHL1** | four and a half LIM domains 1 |
| **FLI1** | Fli-1 proto-oncogene, ETS transcription factor |
| **FLNC** | filamin C |
| **FLRT3** | fibronectin leucine rich transmembrane protein 3 |
| **FLT3** | fms related tyrosine kinase 3 |
| **FNBP1** | formin binding protein 1 |
| **FOS** | Fos proto-oncogene, AP-1 transcription factor subunit |
| **FOSL1** | FOS like 1, AP-1 transcription factor subunit |
| **FOXA1** | forkhead box A1 |
| **FOXC1** | forkhead box C1 |
| **FOXC2** | forkhead box C2 |
| **FOXM1** | forkhead box M1 |
| **FOXP3** | forkhead box P3 |
| **FREM2** | FRAS1 related extracellular matrix protein 2 |
| **FST** | follistatin |
| **FSTL1** | follistatin like 1 |
| **FSTL3** | follistatin like 3 |
| **FUT3** | fucosyltransferase 3 (Lewis blood group) |
| **FXYD3** | FXYD domain containing ion transport regulator 3 |
| **FZD10** | frizzled class receptor 10 |
| **FZD7** | frizzled class receptor 7 |
| **FZD8** | frizzled class receptor 8 |
| **FZD9** | frizzled class receptor 9 |
| **GABRP** | gamma-aminobutyric acid type A receptor pi subunit |
| **GADD45A** | growth arrest and DNA damage inducible alpha |
| **GADD45B** | growth arrest and DNA damage inducible beta |
| **GADD45G** | growth arrest and DNA damage inducible gamma |
| **GAS1** | growth arrest specific 1 |
| **GATA3** | GATA binding protein 3 |
| **GATA4** | GATA binding protein 4 |
| **GDF15** | growth differentiation factor 15 |
| **GDF5** | growth differentiation factor 5 |
| **GGH** | gamma-glutamyl hydrolase |
| **GHR** | growth hormone receptor |
| **GJB2** | gap junction protein beta 2 |
| **GLI3** | GLI family zinc finger 3 |
| **GNG4** | G protein subunit gamma 4 |
| **GNLY** | granulysin |
| **GPC4** | glypican 4 |
| **GPR160** | G protein-coupled receptor 160 |
| **GPX3** | glutathione peroxidase 3 |
| **GRB2** | growth factor receptor bound protein 2 |
| **GRB7** | growth factor receptor bound protein 7 |
| **GREM1** | gremlin 1, DAN family BMP antagonist |
| **GRIA3** | glutamate ionotropic receptor AMPA type subunit 3 |
| **GRIN1** | glutamate ionotropic receptor NMDA type subunit 1 |
| **GRIN2A** | glutamate ionotropic receptor NMDA type subunit 2A |
| **GSK3B** | glycogen synthase kinase 3 beta |
| **GTF2H2** | general transcription factor IIH subunit 2 |
| **GZMA** | granzyme A |
| **GZMB** | granzyme B |
| **GZMH** | granzyme H |
| **GZMM** | granzyme M |
| **HAPLN1** | hyaluronan and proteoglycan link protein 1 |
| **HAS1** | hyaluronan synthase 1 |
| **HBB** | hemoglobin subunit beta |
| **HDAC1** | histone deacetylase 1 |
| **HDAC10** | histone deacetylase 10 |
| **HDAC11** | histone deacetylase 11 |
| **HDAC2** | histone deacetylase 2 |
| **HDAC5** | histone deacetylase 5 |
| **HDAC6** | histone deacetylase 6 |
| **HDC** | histidine decarboxylase |
| **HEG1** | heart development protein with EGF like domains 1 |
| **HELLS** | helicase, lymphoid specific |
| **HEMK1** | HemK methyltransferase family member 1 |
| **HES1** | hes family bHLH transcription factor 1 |
| **HGF** | hepatocyte growth factor |
| **HIF1A** | hypoxia inducible factor 1 alpha subunit |
| **HIST1H1C** | histone cluster 1 H1 family member c |
| **HIST1H2BH** | histone cluster 1 H2B family member h |
| **HIST1H3H** | histone cluster 1 H3 family member h |
| **HIST3H2BB** | histone cluster 3 H2B family member b |
| **HK2** | hexokinase 2 |
| **HLA-A** | H1 histone family member 0 |
| **HLA-B** | major histocompatibility complex, class I, B |
| **HLA-C** | major histocompatibility complex, class I, C |
| **HLA-DMA** | major histocompatibility complex, class II, DM alpha |
| **HLA-DMB** | major histocompatibility complex, class II, DM beta |
| **HLA-DOB** | major histocompatibility complex, class II, DO beta |
| **HLA-DPA1** | major histocompatibility complex, class II, DP alpha 1 |
| **HLA-DPB1** | major histocompatibility complex, class II, DP beta 1 |
| **HLA-DQA1** | major histocompatibility complex, class II, DQ alpha 1 |
| **HLA-DQB1** | major histocompatibility complex, class II, DQ beta 1 |
| **HLA-DRA** | major histocompatibility complex, class II, DR alpha |
| **HLA-DRB1** | major histocompatibility complex, class II, DR beta 1 |
| **HLA-E** | major histocompatibility complex, class I, E |
| **HMGA1** | high mobility group AT-hook 1 |
| **HNF1A** | HNF1 homeobox A |
| **HOXA5** | homeobox A5 |
| **HOXA7** | homeobox A7 |
| **HOXA9** | homeobox A9 |
| **HOXB13** | homeobox B13 |
| **HOXB3** | homeobox B3 |
| **HSPA2** | heat shock protein family A (Hsp70) member 2 |
| **IBSP** | integrin binding sialoprotein |
| **ICAM1** | intercellular adhesion molecule 1 |
| **ID1** | inhibitor of DNA binding 1, HLH protein |
| **ID2** | inhibitor of DNA binding 2 |
| **ID4** | inhibitor of DNA binding 4, HLH protein |
| **IDO1** | indoleamine 2,3-dioxygenase 1 |
| **IFT140** | intraflagellar transport 140 |
| **IGF1** | insulin like growth factor 1 |
| **IGF1R** | insulin like growth factor 1 receptor |
| **IKZF3** | IKAROS family zinc finger 3 |
| **IL10RA** | interleukin 10 receptor subunit alpha |
| **IL11RA** | interleukin 11 receptor subunit alpha |
| **IL12RB2** | interleukin 12 receptor subunit beta 2 |
| **IL13RA1** | interleukin 13 receptor subunit alpha 1 |
| **IL1B** | interleukin 1 beta |
| **IL1R2** | interleukin 1 receptor type 2 |
| **IL1RN** | interleukin 1 receptor antagonist |
| **IL20RA** | interleukin 20 receptor subunit alpha |
| **IL20RB** | interleukin 20 receptor subunit beta |
| **IL22RA2** | interleukin 22 receptor subunit alpha 2 |
| **IL24** | interleukin 24 |
| **IL2RA** | interleukin 2 receptor subunit alpha |
| **IL2RB** | interleukin 2 receptor subunit beta |
| **IL3RA** | interleukin 3 receptor subunit alpha |
| **IL4R** | interleukin 4 receptor |
| **IL6** | interleukin 6 |
| **IL6R** | interleukin 6 receptor |
| **IL7R** | interleukin 7 receptor |
| **INHBA** | inhibin beta A subunit |
| **INHBB** | inhibin beta B subunit |
| **IRF6** | interferon regulatory factor 6 |
| **IRX1** | iroquois homeobox 1 |
| **ISG15** | ISG15 ubiquitin-like modifier |
| **ISM1** | isthmin 1 |
| **ITGA6** | integrin subunit alpha 6 |
| **ITGAV** | integrin subunit alpha V |
| **ITGB1** | integrin subunit beta 1 |
| **ITGB3** | integrin subunit beta 3 |
| **ITGB6** | integrin subunit beta 6 |
| **ITPR1** | inositol 1,4,5-trisphosphate receptor type 1 |
| **JAG1** | jagged 1 |
| **JAG2** | jagged 2 |
| **JAK1** | Janus kinase 1 |
| **JAK2** | Janus kinase 2 |
| **JAK3** | Janus kinase 3 |
| **JAM2** | junctional adhesion molecule 2 |
| **JCAD** | junctional cadherin 5 associated |
| **JUN** | Jun proto-oncogene, AP-1 transcription factor subunit |
| **KAT2B** | lysine acetyltransferase 2B |
| **KCNB1** | potassium voltage-gated channel subfamily B member 1 |
| **KDR** | kinase insert domain receptor |
| **KIAA0040** | KIAA0040 |
| **KIF11** | kinesin family member 11 |
| **KIF14** | kinesin family member 14 |
| **KIF23** | kinesin family member 23 |
| **KIF2C** | kinesin family member 2C |
| **KIFC1** | kinesin family member C1 |
| **KIT** | KIT proto-oncogene receptor tyrosine kinase |
| **KLRK1** | killer cell lectin like receptor K1 |
| **KRT14** | keratin 14 |
| **KRT17** | keratin 17 |
| **KRT5** | keratin 5 |
| **KRT6B** | keratin 6B |
| **KRT7** | keratin 7 |
| **LAD1** | ladinin 1 |
| **LAG3** | lymphocyte activating 3 |
| **LAMA3** | laminin subunit alpha 3 |
| **LAMB3** | laminin subunit beta 3 |
| **LAMC2** | laminin subunit gamma 2 |
| **LEF1** | lymphoid enhancer binding factor 1 |
| **LEFTY2** | left-right determination factor 2 |
| **LEMD1** | LEM domain containing 1 |
| **LEP** | leptin |
| **LEPR** | leptin receptor |
| **LFNG** | LFNG O-fucosylpeptide 3-beta-N-acetylglucosaminyltransferase |
| **LIF** | LIF, interleukin 6 family cytokine |
| **LIFR** | LIF receptor alpha |
| **LINC02381** | long intergenic non-protein coding RNA 2381 |
| **LPL** | lipoprotein lipase |
| **LRP2** | LDL receptor related protein 2 |
| **LRRC32** | leucine rich repeat containing 32 |
| **LTB** | lymphotoxin beta |
| **LTBP1** | latent transforming growth factor beta binding protein 1 |
| **MAD2L1** | mitotic arrest deficient 2 like 1 |
| **MAF** | MAF bZIP transcription factor |
| **MAML2** | mastermind like transcriptional coactivator 2 |
| **MAP2K4** | mitogen-activated protein kinase kinase 4 |
| **MAP3K12** | mitogen-activated protein kinase kinase kinase 12 |
| **MAPK1** | mitogen-activated protein kinase 1 |
| **MAPK10** | mitogen-activated protein kinase 10 |
| **MAPK3** | mitogen-activated protein kinase 3 |
| **MAPK8IP2** | mitogen-activated protein kinase 8 interacting protein 2 |
| **MAPT** | microtubule associated protein tau |
| **MARCO** | macrophage receptor with collagenous structure |
| **MCM2** | minichromosome maintenance complex component 2 |
| **MCM3** | minichromosome maintenance complex component 3 |
| **MDM2** | MDM2 proto-oncogene |
| **MED1** | mediator complex subunit 1 |
| **MELK** | maternal embryonic leucine zipper kinase |
| **MEOX2** | mesenchyme homeobox 2 |
| **MET** | MET proto-oncogene, receptor tyrosine kinase |
| **MFNG** | MFNG O-fucosylpeptide 3-beta-N-acetylglucosaminyltransferase |
| **MIA** | melanoma inhibitory activity |
| **MIS18A** | MIS18 kinetochore protein A |
| **MKI67** | marker of proliferation Ki-67 |
| **MLH1** | mutL homolog 1 |
| **MLLT3** | MLLT3, super elongation complex subunit |
| **MLPH** | melanophilin |
| **MME** | membrane metalloendopeptidase |
| **MMP11** | matrix metallopeptidase 11 |
| **MMP14** | matrix metallopeptidase 14 |
| **MMP3** | matrix metallopeptidase 3 |
| **MMP7** | matrix metallopeptidase 7 |
| **MMP9** | matrix metallopeptidase 9 |
| **MMRN2** | multimerin 2 |
| **MRE11** | MRE11 homolog, double strand break repair nuclease |
| **MS4A2** | membrane spanning 4-domains A2 |
| **MSR1** | macrophage scavenger receptor 1 |
| **MT1G** | metallothionein 1G |
| **MTOR** | mechanistic target of rapamycin kinase |
| **MUC1** | mucin 1, cell surface associated |
| **MUS81** | MUS81 structure-specific endonuclease subunit |
| **MYBL2** | MYB proto-oncogene like 2 |
| **MYC** | MYC proto-oncogene, bHLH transcription factor |
| **MYCN** | MYCN proto-oncogene, bHLH transcription factor |
| **MYCT1** | MYC target 1 |
| **NASP** | nuclear autoantigenic sperm protein |
| **NAT1** | N-acetyltransferase 1 |
| **NCAM1** | neural cell adhesion molecule 1 |
| **NCAPH2** | non-SMC condensin II complex subunit H2 |
| **NDC80** | NDC80, kinetochore complex component |
| **NDP** | NDP, norrin cystine knot growth factor |
| **NEIL1** | nei like DNA glycosylase 1 |
| **NEIL2** | nei like DNA glycosylase 2 |
| **NEIL3** | nei like DNA glycosylase 3 |
| **NEO1** | neogenin 1 |
| **NETO2** | neuropilin and tolloid like 2 |
| **NFATC1** | nuclear factor of activated T-cells 1 |
| **NFKBIZ** | NFKB inhibitor zeta |
| **NGFR** | nerve growth factor receptor |
| **NKG7** | natural killer cell granule protein 7 |
| **NOD2** | nucleotide binding oligomerization domain containing 2 |
| **NOTCH1** | notch 1 |
| **NOTCH2** | notch 2 |
| **NOTCH3** | notch 3 |
| **NPEPPS** | aminopeptidase puromycin sensitive |
| **NPR1** | natriuretic peptide receptor 1 |
| **NR4A1** | nuclear receptor subfamily 4 group A member 1 |
| **NR4A3** | nuclear receptor subfamily 4 group A member 3 |
| **NRCAM** | neuronal cell adhesion molecule |
| **NRXN1** | neurexin 1 |
| **NRXN3** | neurexin 3 |
| **NSD1** | nuclear receptor binding SET domain protein 1 |
| **NSD3** | nuclear receptor binding SET domain protein 3 |
| **NTRK2** | neurotrophic receptor tyrosine kinase 2 |
| **NUDT1** | nudix hydrolase 1 |
| **NUF2** | NUF2, NDC80 kinetochore complex component |
| **NUMBL** | NUMB like, endocytic adaptor protein |
| **NUPR1** | nuclear protein 1, transcriptional regulator |
| **OAS3** | 2'-5'-oligoadenylate synthetase 3 |
| **OCLN** | occludin |
| **OGN** | osteoglycin |
| **OLFML2B** | olfactomedin like 2B |
| **ORC6** | origin recognition complex subunit 6 |
| **PALB2** | partner and localizer of BRCA2 |
| **PALMD** | palmdelphin |
| **PARP1** | poly(ADP-ribose) polymerase 1 |
| **PARP2** | poly(ADP-ribose) polymerase 2 |
| **PARP4** | poly(ADP-ribose) polymerase family member 4 |
| **PAX5** | paired box 5 |
| **PAX8** | paired box 8 |
| **PBX3** | PBX homeobox 3 |
| **PCK1** | phosphoenolpyruvate carboxykinase 1 |
| **PCNA** | proliferating cell nuclear antigen |
| **PDCD1** | programmed cell death 1 |
| **PDCD1LG2** | programmed cell death 1 ligand 2 |
| **PDE9A** | phosphodiesterase 9A |
| **PDGFB** | platelet derived growth factor subunit B |
| **PDGFRA** | platelet derived growth factor receptor alpha |
| **PDGFRB** | platelet derived growth factor receptor beta |
| **PDK4** | pyruvate dehydrogenase kinase 4 |
| **PECAM1** | platelet and endothelial cell adhesion molecule 1 |
| **PFDN2** | prefoldin subunit 2 |
| **PGK1** | phosphoglycerate kinase 1 |
| **PGR** | progesterone receptor |
| **PHGDH** | phosphoglycerate dehydrogenase |
| **PIK3CA** | phosphatidylinositol-4,5-bisphosphate 3-kinase catalytic subunit alpha |
| **PIK3CD** | phosphatidylinositol-4,5-bisphosphate 3-kinase catalytic subunit delta |
| **PIK3CG** | phosphatidylinositol-4,5-bisphosphate 3-kinase catalytic subunit gamma |
| **PIK3R1** | phosphoinositide-3-kinase regulatory subunit 1 |
| **PIK3R2** | phosphoinositide-3-kinase regulatory subunit 2 |
| **PIK3R3** | phosphoinositide-3-kinase regulatory subunit 3 |
| **PIK3R5** | phosphoinositide-3-kinase regulatory subunit 5 |
| **PIM1** | Pim-1 proto-oncogene, serine/threonine kinase |
| **PIP** | prolactin induced protein |
| **PKMYT1** | protein kinase, membrane associated tyrosine/threonine 1 |
| **PLA1A** | phospholipase A1 member A |
| **PLA2G2A** | phospholipase A2 group IIA |
| **PLA2G3** | phospholipase A2 group III |
| **PLA2G4A** | phospholipase A2 group IVA |
| **PLA2G4F** | phospholipase A2 group IVF |
| **PLAT** | plasminogen activator, tissue type |
| **PLCB1** | phospholipase C beta 1 |
| **PLCB4** | phospholipase C beta 4 |
| **PLCE1** | phospholipase C epsilon 1 |
| **PLD1** | phospholipase D1 |
| **PMS2** | PMS1 homolog 2, mismatch repair system component |
| **POLD1** | DNA polymerase delta 1, catalytic subunit |
| **POLQ** | DNA polymerase theta |
| **POPDC3** | popeye domain containing 3 |
| **PPARG** | peroxisome proliferator activated receptor gamma |
| **PPARGC1A** | PPARG coactivator 1 alpha |
| **PPP2CB** | protein phosphatase 2 catalytic subunit beta |
| **PPP2R1A** | protein phosphatase 2 scaffold subunit Aalpha |
| **PPP2R2C** | protein phosphatase 2 regulatory subunit Bgamma |
| **PRC1** | protein regulator of cytokinesis 1 |
| **PREP** | prolyl endopeptidase |
| **PRF1** | perforin 1 |
| **PRKAA2** | protein kinase AMP-activated catalytic subunit alpha 2 |
| **PRKACA** | protein kinase cAMP-activated catalytic subunit alpha |
| **PRKACB** | protein kinase cAMP-activated catalytic subunit beta |
| **PRKCA** | protein kinase C alpha |
| **PRKCB** | protein kinase C beta |
| **PRKDC** | protein kinase, DNA-activated, catalytic polypeptide |
| **PRKX** | protein kinase, X-linked |
| **PRLR** | prolactin receptor |
| **PROM1** | prominin 1 |
| **PSAT1** | phosphoserine aminotransferase 1 |
| **PSMB10** | proteasome subunit beta 10 |
| **PSMB7** | proteasome subunit beta 7 |
| **PSMB9** | proteasome subunit beta 9 |
| **PTCH1** | patched 1 |
| **PTEN** | phosphatase and tensin homolog |
| **PTGDS** | prostaglandin D2 synthase |
| **PTGER3** | prostaglandin E receptor 3 |
| **PTGS2** | prostaglandin-endoperoxide synthase 2 |
| **PTTG1** | pituitary tumor-transforming 1 |
| **PYCARD** | PYD and CARD domain containing |
| **RAC2** | Rac family small GTPase 2 |
| **RAC3** | Rac family small GTPase 3 |
| **RAD51** | RAD51 recombinase |
| **RAD51C** | RAD51 paralog C |
| **RAD52** | RAD52 homolog, DNA repair protein |
| **RAD54L** | RAD54 like |
| **RARRES3** | retinoic acid receptor responder 3 |
| **RASAL1** | RAS protein activator like 1 |
| **RASGRF1** | Ras protein specific guanine nucleotide releasing factor 1 |
| **RASGRF2** | Ras protein specific guanine nucleotide releasing factor 2 |
| **RASGRP1** | RAS guanyl releasing protein 1 |
| **RB1** | RB transcriptional corepressor 1 |
| **RBL1** | RB transcriptional corepressor like 1 |
| **RBL2** | RB transcriptional corepressor like 2 |
| **RBX1** | ring-box 1 |
| **RELN** | reelin |
| **RFC4** | replication factor C subunit 4 |
| **RNASE2** | ribonuclease A family member 2 |
| **RNF103** | ring finger protein 103 |
| **ROBO4** | roundabout guidance receptor 4 |
| **ROCK1** | Rho associated coiled-coil containing protein kinase 1 |
| **ROCK2** | Rho associated coiled-coil containing protein kinase 2 |
| **RORA** | RAR related orphan receptor A |
| **RORB** | RAR related orphan receptor B |
| **RPS6KA5** | ribosomal protein S6 kinase A5 |
| **RPS6KB1** | ribosomal protein S6 kinase B1 |
| **RPS6KB2** | ribosomal protein S6 kinase B2 |
| **RRM2** | ribonucleotide reductase regulatory subunit M2 |
| **RUNX3** | runt related transcription factor 3 |
| **S100A14** | S100 calcium binding protein A14 |
| **S100A7** | S100 calcium binding protein A7 |
| **S1PR1** | sphingosine-1-phosphate receptor 1 |
| **SCARA5** | scavenger receptor class A member 5 |
| **SCUBE2** | signal peptide, CUB domain and EGF like domain containing 2 |
| **SELE** | selectin E |
| **SERBP1** | SERPINE1 mRNA binding protein 1 |
| **SERPINB5** | serpin family B member 5 |
| **SERPINH1** | serpin family H member 1 |
| **SFN** | stratifin |
| **SFRP1** | secreted frizzled related protein 1 |
| **SFRP2** | secreted frizzled related protein 2 |
| **SFRP4** | secreted frizzled related protein 4 |
| **SHC2** | SHC adaptor protein 2 |
| **SHC4** | SHC adaptor protein 4 |
| **SHE** | Src homology 2 domain containing E |
| **SHMT2** | serine hydroxymethyltransferase 2 |
| **SIDT1** | SID1 transmembrane family member 1 |
| **SIGIRR** | single Ig and TIR domain containing |
| **SIX1** | SIX homeobox 1 |
| **SKA3** | spindle and kinetochore associated complex subunit 3 |
| **SKP1** | S-phase kinase associated protein 1 |
| **SKP2** | S-phase kinase associated protein 2 |
| **SLC2A1** | solute carrier family 2 member 1 |
| **SLC39A6** | solute carrier family 39 member 6 |
| **SLC44A4** | solute carrier family 44 member 4 |
| **SLPI** | secretory leukocyte peptidase inhibitor |
| **SMAD1** | SMAD family member 1 |
| **SMAD3** | SMAD family member 3 |
| **SMAD4** | SMAD family member 4 |
| **SMAD5** | SMAD family member 5 |
| **SMC1B** | structural maintenance of chromosomes 1B |
| **SMO** | smoothened, frizzled class receptor |
| **SMURF2** | SMAD specific E3 ubiquitin protein ligase 2 |
| **SNAI1** | snail family transcriptional repressor 1 |
| **SNAI2** | snail family transcriptional repressor 2 |
| **SOCS1** | suppressor of cytokine signaling 1 |
| **SOCS2** | suppressor of cytokine signaling 2 |
| **SOCS3** | suppressor of cytokine signaling 3 |
| **SOX10** | SRY-box 10 |
| **SOX17** | SRY-box 17 |
| **SOX2** | SRY-box 2 |
| **SOX9** | SRY-box 9 |
| **SP1** | Sp1 transcription factor |
| **SPC25** | SPC25, NDC80 kinetochore complex component |
| **SPDEF** | SAM pointed domain containing ETS transcription factor |
| **SPN** | sialophorin |
| **SPP1** | secreted phosphoprotein 1 |
| **SPRY1** | sprouty RTK signaling antagonist 1 |
| **SPRY2** | sprouty RTK signaling antagonist 2 |
| **SPRY4** | sprouty RTK signaling antagonist 4 |
| **SRPX** | sushi repeat containing protein, X-linked |
| **ST6GALNAC2** | ST6 N-acetylgalactosaminide alpha-2,6-sialyltransferase 2 |
| **STAT1** | signal transducer and activator of transcription 1 |
| **STC1** | stanniocalcin 1 |
| **SUV39H2** | suppressor of variegation 3-9 homolog 2 |
| **SYTL4** | synaptotagmin like 4 |
| **TAP1** | transporter 1, ATP binding cassette subfamily B member |
| **TAP2** | transporter 2, ATP binding cassette subfamily B member |
| **TAPBP** | TAP binding protein |
| **TBC1D9** | TBC1 domain family member 9 |
| **TBX1** | T-box 1 |
| **TCEAL1** | transcription elongation factor A like 1 |
| **TCF4** | transcription factor 4 |
| **TCF7L1** | transcription factor 7 like 1 |
| **TEK** | TEK receptor tyrosine kinase |
| **TFDP1** | transcription factor Dp-1 |
| **TFF1** | trefoil factor 1 |
| **TFF3** | trefoil factor 3 |
| **TGFB1** | transforming growth factor beta 1 |
| **TGFB2** | transforming growth factor beta 2 |
| **TGFB3** | transforming growth factor beta 3 |
| **TGFBR2** | transforming growth factor beta receptor 2 |
| **THBS1** | thrombospondin 1 |
| **THBS2** | thrombospondin 2 |
| **THBS4** | thrombospondin 4 |
| **THY1** | Thy-1 cell surface antigen |
| **TIE1** | tyrosine kinase with immunoglobulin like and EGF like domains 1 |
| **TIGIT** | T-cell immunoreceptor with Ig and ITIM domains |
| **TIMP4** | TIMP metallopeptidase inhibitor 4 |
| **TLE3** | transducin like enhancer of split 3 |
| **TLR4** | toll like receptor 4 |
| **TLX1** | T-cell leukemia homeobox 1 |
| **TMEM45B** | transmembrane protein 45B |
| **TMPRSS2** | transmembrane protease, serine 2 |
| **TMPRSS4** | transmembrane protease, serine 4 |
| **TNF** | tumor necrosis factor |
| **TNFAIP6** | TNF alpha induced protein 6 |
| **TNFSF10** | TNF superfamily member 10 |
| **TNKS** | tankyrase |
| **TNKS2** | tankyrase 2 |
| **TNN** | tenascin N |
| **TOP2A** | DNA topoisomerase II alpha |
| **TP53** | tumor protein p53 |
| **TPSAB1** | tryptase alpha/beta 1 |
| **TRIP13** | thyroid hormone receptor interactor 13 |
| **TSPAN1** | tetraspanin 1 |
| **TSPAN7** | tetraspanin 7 |
| **TTK** | TTK protein kinase |
| **TTYH1** | tweety family member 1 |
| **TUBA4A** | tubulin alpha 4a |
| **TWIST1** | twist family bHLH transcription factor 1 |
| **TWIST2** | twist family bHLH transcription factor 2 |
| **TYK2** | tyrosine kinase 2 |
| **TYMP** | thymidine phosphorylase |
| **TYMS** | thymidylate synthetase |
| **UBE2C** | ubiquitin conjugating enzyme E2 C |
| **UBE2T** | ubiquitin conjugating enzyme E2 T |
| **VCAN** | versican |
| **VEGFA** | vascular endothelial growth factor A |
| **VEGFD** | vascular endothelial growth factor D |
| **VIM** | vimentin |
| **VIT** | vitrin |
| **WDR77** | WD repeat domain 77 |
| **WEE1** | WEE1 G2 checkpoint kinase |
| **WIF1** | WNT inhibitory factor 1 |
| **WNT10A** | Wnt family member 10A |
| **WNT11** | Wnt family member 11 |
| **WNT2** | Wnt family member 2 |
| **WNT4** | Wnt family member 4 |
| **WNT5A** | Wnt family member 5A |
| **WNT5B** | Wnt family member 5B |
| **WNT6** | Wnt family member 6 |
| **WNT7B** | Wnt family member 7B |
| **WRN** | Werner syndrome RecQ like helicase |
| **WT1** | Wilms tumor 1 |
| **XRCC2** | X-ray repair cross complementing 2 |
| **XRCC3** | X-ray repair cross complementing 3 |
| **ZBTB16** | zinc finger and BTB domain containing 16 |
| **ZEB1** | zinc finger E-box binding homeobox 1 |
| **ZEB2** | zinc finger E-box binding homeobox 2 |
| **ZFPM2** | zinc finger protein, FOG family member 2 |
| **ZFYVE9** | zinc finger FYVE-type containing 9 |
| **ZIC2** | Zic family member 2 |
| **ZNF205** | zinc finger protein 205 |
| **ABCF1** | ATP binding cassette subfamily F member 1 |
| **ACTB** | actin beta |
| **G6PD** | glucose-6-phosphate dehydrogenase |
| **GUSB** | glucuronidase beta |
| **MRPL19** | mitochondrial ribosomal protein L19 |
| **NRDE2** | NRDE-2, necessary for RNA interference, domain containing |
| **OAZ1** | ornithine decarboxylase antizyme 1 |
| **POLR2A** | RNA polymerase II subunit A |
| **PSMC4** | proteasome 26S subunit, ATPase 4 |
| **PUM1** | pumilio RNA binding family member 1 |
| **RPLP0** | ribosomal protein lateral stalk subunit P0 |
| **SDHA** | succinate dehydrogenase complex flavoprotein subunit A |
| **SF3A1** | splicing factor 3a subunit 1 |
| **STK11IP** | serine/threonine kinase 11 interacting protein |
| **TBC1D10B** | TBC1 domain family member 10B |
| **TBP** | TATA-box binding protein |
| **TFRC** | transferrin receptor |
| **UBB** | ubiquitin B |
