# Supplementary material for: Path to Clonal Theranostics in Luminal Breast Cancers
Source: Front Oncol. 2022 Jan 13;11:802177. doi: 10.3389/fonc.2021.802177 (PMC8793283; doi:10.3389/fonc.2021.802177)
Supplement: Supplementary Material 1 — TCGA database of mutations and CNV alterations in early and advanced breast cancers. [file DataSheet_1.zip › Data Sheet 3.doc]

**Supplementary material 3:** CDx panel gene list

ABL1

ACVR1B

AKT1

AKT2

AKT3

ALK

ALOX12B

AMER1

APC

AR

ARAF

ARFRP1

ARID1A

ASXL1

ATM

ATR

ATRX

AURKA

AURKB

AXIN1

AXL

BAP1

BARD1

BCL2

BCL2L1

BCL2L2

BCL6

BCOR

BCORL1

BRAF

BRCA1

BRCA2

BRD4

BRIP1

BTG1

BTG2

BTK

C11orf30

CALR

CARD11

CASP8

CBFB

CBL

CCND1

CCND2

CCND3

CCNE1

CD22

CD274

CD70

CD79A

CD79B

CDC73

CDH1

CDK12

CDK4

CDK6

CDK8

CDKN1A

CDKN1B

CDKN2A

CDKN2B

CDKN2C

CEBPA

CHEK1

CHEK2

CIC

CREBBP

CRKL

CSF1R

CSF3R

CTCF

CTNNA1

CTNNB1

CUL3

CUL4A

CXCR4

CYP17A1

DAXX

DDR1

DDR2

DIS3

DNMT3A

DOT1L

EED

EGFR

EP300

EPHA3

EPHB1

EPHB4

ERBB2

ERBB3

ERBB4

ERCC4

ERG

ERRFI1

ESR1

EZH2

FAM46C

FANCA

FANCC

FANCG

FANCL

FAS

FBXW7

FGF10

FGF12

FGF14

FGF19

FGF23

FGF3

FGF4

FGF6

FGFR1

FGFR2

FGFR3

FGFR4

FH

FLCN

FLT1

FLT3

FOXL2

FUBP1

GABRA6

GATA3

GATA4

GATA6

GID4

GNA11

GNA13

GNAQ

GNAS

GRM3

GSK3B

H3F3A

HDAC1

HGF

HNF1A

HRAS

HSD3B1

ID3

IDH1

IDH2

IGF1R

IKBKE

IKZF1

INPP4B

IRF2

IRF4

IRS2

JAK1

JAK2

JAK3

JUN

KDM5A

KDM5C

KDM6A

KDR

KEAP1

KEL

KIT

KLHL6

KMT2A

KMT2D

KRAS

LTK

LYN

MAF

MAP2K1

MAP2K2

MAP2K4

MAP3K1

MAP3K13

MAPK1

MCL1

MDM2

MDM4

MED12

MEF2B

MEN1

MERTK

MET

MITF

MKNK1

MLH1

MPL

MRE11A

MSH2

MSH3

MSH6

MST1R

MTAP

MTOR

MUTYH

MYC

MYCL

MYCN

MYD88

NBN

NF1

NF2

NFE2L2

NFKBIA

NKX2-1

NOTCH1

NOTCH2

NOTCH3

NPM1

NRAS

NT5C2

NTRK1

NTRK2

NTRK3

P2RY8

Page

PALB2

PARK2

PARP1

PARP2

PARP3

PAX5

PBRM1

PDCD1

PDCD1LG2

PDGFRA

PDGFRB

PDK1

PIK3C2B

PIK3C2G

PIK3CA

PIK3CB

PIK3R1

PIM1

PMS2

POLD1

POLE

PPARG

PPP2R1A

PPP2R2A

PRDM1

PRKAR1A

PRKCI

PTCH1

PTEN

PTPN11

PTPRO

QKI

RAC1

RAD21

RAD51

RAD51B

RAD51C

RAD51D

RAD52

RAD54L

RAF1

RAL-0003-03

RARA

RB1

RBM10

REL

RET

RICTOR

RNF43

ROS1

RPTOR

SDHA

SDHB

SDHC

SDHD

SETD2

SF3B1

SGK1

SMAD2

SMAD4

SMARCA4

SMARCB1

SMO

SNCAIP

SOCS1

SOX2

SOX9

SPEN

SPOP

SRC

STAG2

STAT3

STK11

SUFU

SYK

TBX3

TEK

TET2

TGFBR2

TIPARP

TNFAIP3

TNFRSF14

TP53

TSC1

TSC2

TYRO3

U2AF1

VEGFA

VHL

WHSC1

WHSC1L1

WT1

XPO1

XRCC2

ZNF217

ZNF703
