# Supplementary material for: Path to Clonal Theranostics in Luminal Breast Cancers
Source: Front Oncol. 2022 Jan 13;11:802177. doi: 10.3389/fonc.2021.802177 (PMC8793283; doi:10.3389/fonc.2021.802177)
Supplement: Supplementary Material 1 — TCGA database of mutations and CNV alterations in early and advanced breast cancers. [file DataSheet_1.zip › Data Sheet 6.doc]

**Supplementary material 6:** Number of total proteins identified according to (A) tissue types (bone n=3 ; liver n=5 ; nodes n=6 ; skin n=10) or (B) to the sampling method (B), i.e. surgery (n=52), surgical biopsy (n=9) or fine needle core biopsy (n=15). Line in the middle of the box is the median, the upper and lower sides of the box represent the 75th and 25th percentiles respectively, and the outside lines are the extremes.
